# Contribution of hypoxia-inducible factor 1alpha to pathogenesis of sarcomeric hypertrophic cardiomyopathy

**DOI:** 10.1038/s41598-025-85187-9

**Published:** 2025-01-16

**Authors:** Sarala Raj Murthi, Andreas Petry, Bachuki Shashikadze, Jan B. Stöckl, Manuel Schmid, Gianluca Santamaria, Karin Klingel, Damir Kračun, Xinpei Chen, Sabine Bauer, Joachim P. Schmitt, Florian Flenkenthaler, Josh Gorham, Christopher N. Toepfer, David Potěšil, Pavel Hruška, Zbyněk Zdráhal, Zsuzsanna Mayer, Mathieu Klop, Luisa Lehmann, Yishi Qin, Laura Papanakli, Nadine Spielmann, Alessandra Moretti, Thomas Fröhlich, Peter Ewert, Stefan Holdenrieder, Jonathan G. Seidman, Christine E. Seidman, Agnes Görlach, Cordula M. Wolf

**Affiliations:** 1 Department of Congenital Heart Defects and Pediatric Cardiology, German Heart Center Munich, TUM University Hospital, School of Medicine & Health, Technical University of Munich, Munich, Germany; 2 Experimental and Molecular Pediatric Cardiology, Department of Congenital Heart Defects and Pediatric Cardiology, German Heart Center Munich, TUM University Hospital, School of Medicine & Health, Technical University of Munich, Munich, Germany; 3 Laboratory for Functional Genome Analysis LAFUGA Gene Center, LMU Munich, Munich, Germany; 4 Department of Genetics, Harvard Medical School, Boston, USA; 5 Cardiovascular Medicine, Radcliffe Department of Medicine, University of Oxford, Oxford, UK; 6 Wellcome Centre for Human Genetics, University of Oxford, Oxford, UK; 7 First Department of Medicine and Regenerative Medicine in Cardiovascular Diseases, Klinikum rechts der Isar, School of Medicine & Health, Technical University of Munich, Munich, Germany; 8 Department of Experimental and Clinical Medicine, “Magna Graecia” University of Catanzaro, Catanzaro, Italy; 9 Cardiopathology, Institute for Pathology and Neuropathology, University Hospital of Tübingen, Tübingen, Germany; 10 University Hospital Balgrist, University of Zurich and Institute for Biomechanics, ETH Zurich, Zurich, Switzerland; 11 Institute of Pharmacology, University Hospital Düsseldorf and Cardiovascular Research Institute Düsseldorf (CARID), Heinrich-Heine-University, Düsseldorf, Germany; 12 Mendel Centre for Plant Genomics and Proteomics, Central European Institute of Technology, Masaryk University, Brno, Czech Republic; 13 Institute for Laboratory Medicine, German Heart Center Munich, TUM University Hospital, School of Medicine & Health, Technical University of Munich, Munich, Germany; 14 Experimental Cardiology, Department of Cardiology, German Heart Center Munich, TUM University Hospital, School of Medicine & Health, Technical University of Munich, Munich, Germany; 15 Institute of Experimental Genetics and German Mouse Clinic, Helmholtz Center Munich, German Research Center for Environmental Health, Neuherberg, Germany; 16 School of Medicine and Health, Technical University of Munich, Munich, Germany; 17 DZHK (German Centre for Cardiovascular Research), Partner Site Munich Heart Alliance, Munich, Germany

**Keywords:** Hypertrophic cardiomyopathy, Hypoxia, HIF1A, Hypertrophy, Myocardial fibrosis, Biochemistry, Biological techniques, Genetics, Molecular biology, Biomarkers, Cardiology, Medical research

## Abstract

Hypertrophic cardiomyopathy (HCM) caused by autosomal-dominant mutations in genes coding for structural sarcomeric proteins, is the most common inherited heart disease. HCM is associated with myocardial hypertrophy, fibrosis and ventricular dysfunction. Hypoxia-inducible transcription factor-1α (Hif-1α) is the central master regulators of cellular hypoxia response and associated with HCM. Yet its exact role remains to be elucidated. Therefore, the effect of a cardiomyocyte-specific Hif-1a knockout (cHif1aKO) was studied in an established α-MHC^719/+^ HCM mouse model that exhibits the classical features of human HCM. The results show that Hif-1α protein and HIF targets were upregulated in left ventricular tissue of α-MHC^719/+^ mice. Cardiomyocyte-specific abolishment of Hif-1a blunted the disease phenotype, as evidenced by decreased left ventricular wall thickness, reduced myocardial fibrosis, disordered SRX/DRX state and ROS production. cHif1aKO induced normalization of pro-hypertrophic and pro-fibrotic left ventricular remodeling signaling evidenced on whole transcriptome and proteomics analysis in α-MHC^719/+^ mice. Proteomics of serum samples from patients with early onset HCM revealed significant modulation of HIF. These results demonstrate that HIF signaling is involved in mouse and human HCM pathogenesis. Cardiomyocyte-specific knockout of Hif-1a attenuates disease phenotype in the mouse model. Targeting Hif-1α might serve as a therapeutic option to mitigate HCM disease progression.

## Introduction

Hypertrophic cardiomyopathy (HCM) has a prevalence of 1: 500^1^ and is the most common cause of sudden cardiac death in young athletes^2,3^. The clinical presentation of the disease comprises progressive cardiac hypertrophy, diastolic and systolic dysfunction, and an increased risk of life-threatening arrhythmias. Histologically, cardiomyocyte hypertrophy, myocardial interstitial fibrosis and myofiber disarray are pathognomonic HCM features^4^. HCM is inherited as an autosomal dominant trait with over 1400 mutations in more than 11 genes encoding structural proteins of the sarcomere^4^. Since the description of the first genetic mutation in recent years^5^, HCM research on the molecular and mechanistic basis using animal models^6^ has contributed largely to the understanding of the disease. As such, abnormal biophysical contractility of myocytes^7^, activation of fibrosis-inducing transcription factors^8^, disturbed calcium handling^9^, altered cellular metabolism^10^, and increased disordered relaxed state (DRX) of the filamentous myosin^11^ have been recognized in HCM disease pathogenesis.

Possible therapeutic measures for prevention include avoidance of competitive sports and the implantable cardioverter defibrillators in patients at high risk for malignant arrhythmias. In advanced stages, symptomatic therapies for heart failure or heart transplantation are needed^12,13^.

Myosin inhibition previously shown to be effective in mice^2^ are now approved by the FDA as the first in class treatment for patients with obstructive HCM after a series of successful clinical trials^3,4^.

Due to high genetic variability and different penetrance levels, the clinical manifestations of HCM is very diverse with various presentations of HCM during childhood^14^ to asymptomatic mutation carriers in the elderly^15^. A clear genotype–phenotype correlation has not yet been identified^16^. The great variability of the manifestation of the clinical picture of HCM in patients and in mice models with the same gene mutation^17–19^ suggests that, in addition to the genetic predisposition, various somatic factors are responsible for the development of fibrotic changes, cardiac hypertrophy, cardiomyocyte dysfunction and the risk of arrhythmia.

Activation of inflammatory signaling pathways^20–22^, local hypoxia^23,24^, and oxidative stress^25–27^ were shown in pathologic myocardial remodeling secondary to myocardial ischemia in coronary heart disease, chronic heart failure and atrial fibrillation. Hypoxia-induced transcription factor Hif-1α regulates the cell adaptation to local hypoxia by activating down-stream signaling pathways^28^. It also plays an important role in the regulation of the immune system and inflammation^29^.

Here we hypothesize that Hif-1α plays a crucial role in the pathogenesis of HCM.

This hypothesis was based on several lines of evidence: First, a study on children and adolescents with HCM showed that upregulation of the Hif-1α correlated with increased cardiac hypertrophy and diastolic dysfunction^30^. Second, Hif-1α was shown to be involved in the pathophysiological development of right ventricular hypertrophy^31,32^. Third, inadequate microperfusion in HCM may result in local hypoxia^33,34^ as a potent signal to activate Hif-1α.

Here, we used a well-established HCM mouse model carrying the human Arg719Trp point mutation in the murine myosin heavy chain 6 gene (*Myh6*)^8^ and crossbred it with a previously described cardiac-specific Hif1a knockout mouse line^35^ to decipher the role of Hif-1α in HCM disease pathogenesis. Moreover, we performed serum proteomic analysis in patients affected by early onset of HCM to confirm the contribution of Hif-1α signaling in human HCM.

## Results

### Hif-1α is upregulated in the left ventricle of α-MHC^719/+^ mice displaying HCM

**Fig. 1:**
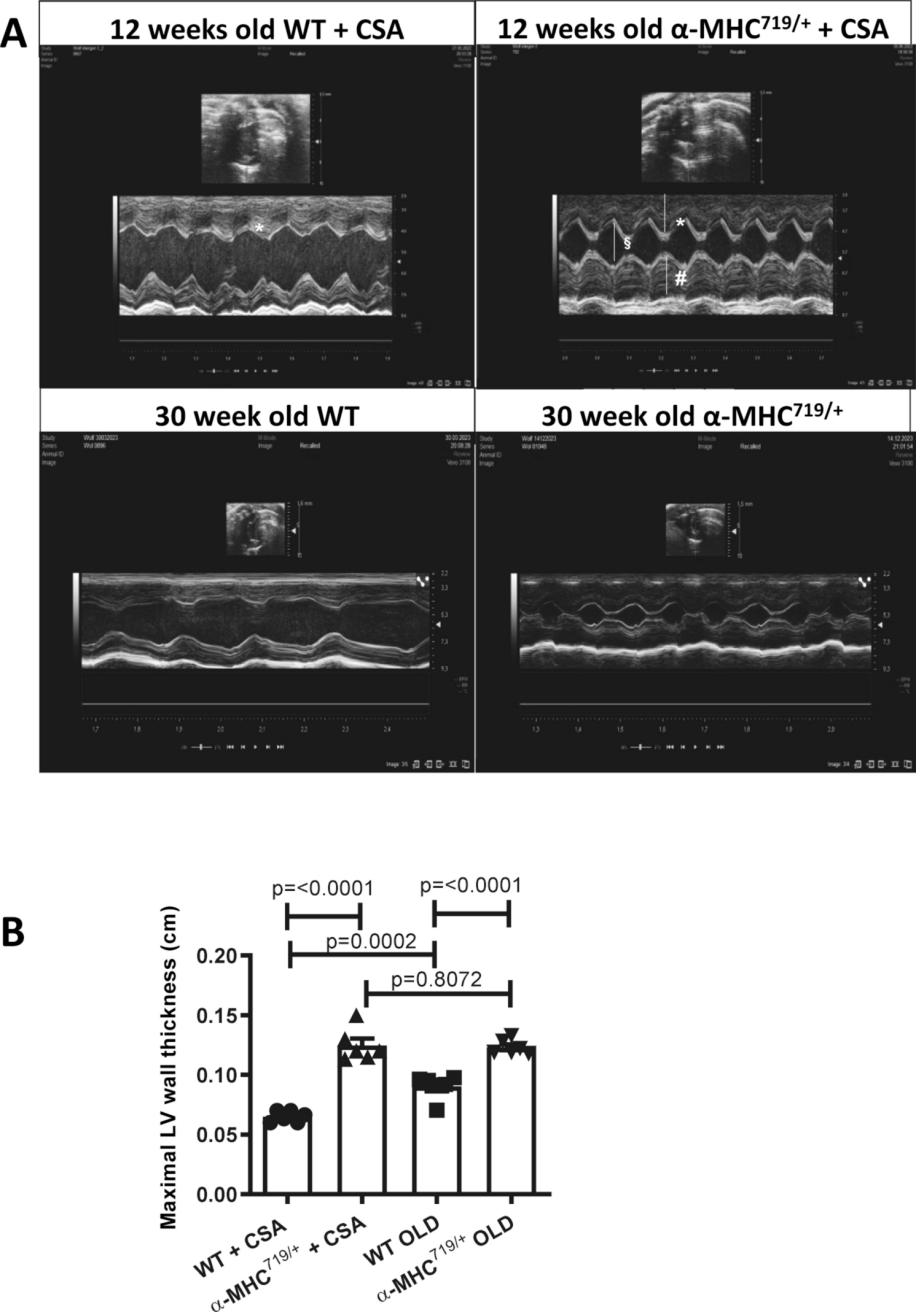
α-MHC^719/+^ mice develop left ventricular hypertrophy over time. (**A**,**B**) Exemplary transthoracic echocardiographic imaging (**A**) and echocardiographic myocardial wall thickness measurements (**B**) of 12 weeks old wildtype (12 weeks old WT + CSA), α-MHC^719/+^ mice (12 weeks old α-MHC^719/+^  + CSA) treated with Cyclosporine, 30 weeks old wildtype mice (30 weeks old WT) and 30 weeks old α-MHC^719/+^ mice (30 weeks old α-MHC^719/+^) without Cyclosporine treatment (n = 6 each group). 12 weeks old α-MHC^719/+^  + CSA and 30 weeks old α-MHC^719/+^ mice show increase of the end-diastolic left ventricular septal (*) and posterior wall thickness (#), and reduced end-diastolic LV diameter (§) when compared to the 12 weeks old WT + CSA and 30 weeks old WT mice on parasternal short axis view. The phenotype arises in the 30 weeks old α-MHC^719/+^ mice naturally and Cyclosporine (CSA) treatment enhances the phenotype in the young 12 weeks old α-MHC^719/+^. Data are presented as mean ± SEM with significances indicated by p values. No significant difference between 12 weeks WT mice treated with CSA and 30 weeks old WT mice without CSA treatment was detected.

**Fig. 2:**
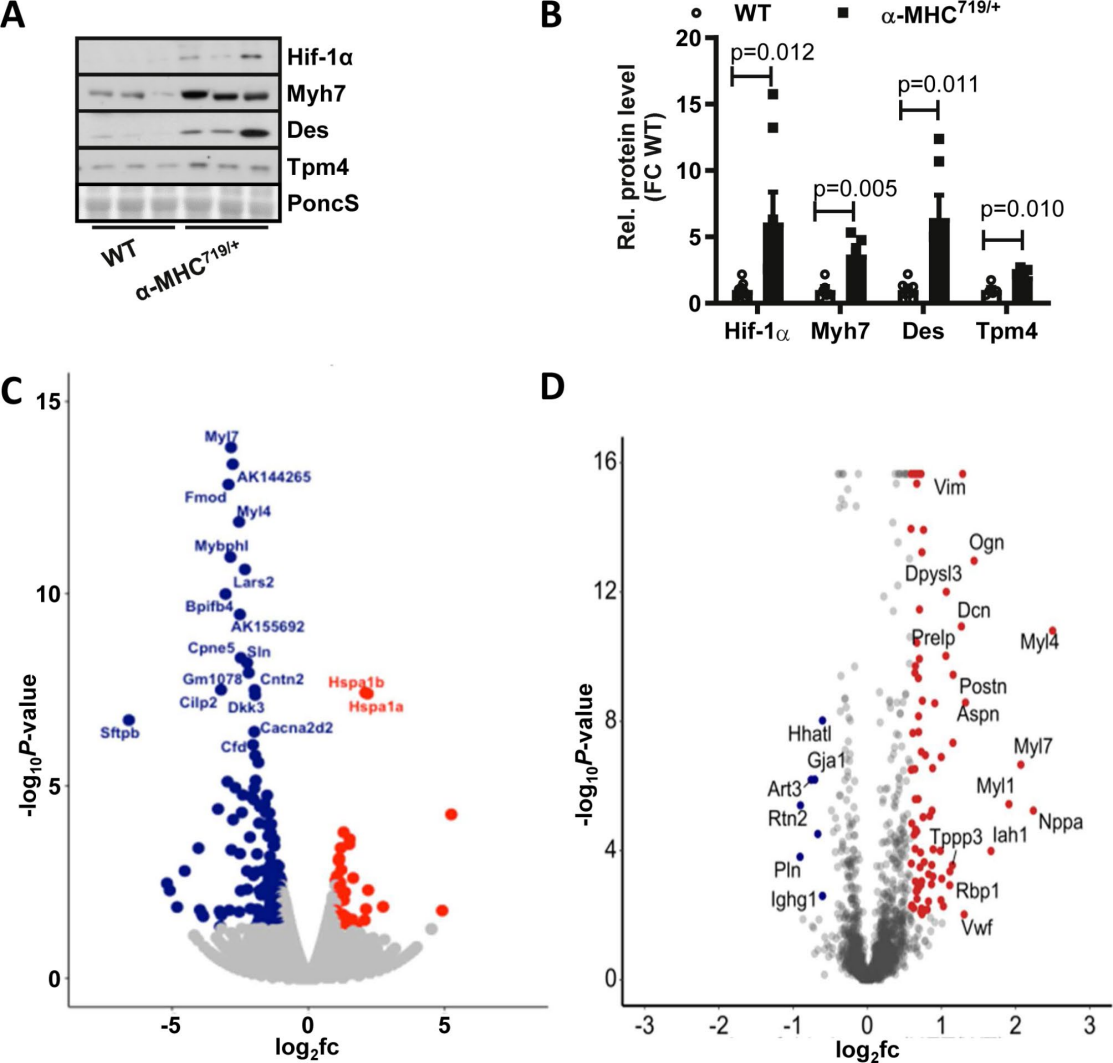
Hif-1α is upregulated in left ventricular tissue of α-MHC^719/+^ mice displaying the pathognomic features of hypertrophic cardiomyopathy. (**A**,**B**) Proteins were isolated from LV tissue from both wild type (WT) and α-MHC^719/+^ mice. Western blot (**A**) and quantification (**B**) of proteins extracted from LV tissue performed for Hif-1α, Myh7, Desmin (Des) and Tpm4 with Actin as loading control. Compared to WT increased relative fold change (FC) of Hif-1α, Myh7, Des and Tpm4 in α-MHC^719/+^ mice (n = 3 each group). Data are presented as mean ± SEM with significances indicated by p values. (**C**,**D**) Differentially expressed genes on LV transcriptome (**C**) or proteome (**D**) analysis of α-MHC^719/+^ vs. WT. Names indicate top ten regulated genes or proteins. Original western blot in (**A**) presented in (Supplemental Fig. 8).

The previously described mouse α-MHC^719/+^ model carrying the common human HCM mutation Arg719Trp (RW) in the mouse *Myh6* gene (α-MHC)^8^ was used in this study. These mice develop HCM by the age of about 20 weeks. Administration of cyclosporine (CSA) accelerates the emergence of HCM so that myocardial hypertrophy and fibrosis are detected at the age of 12 weeks (Supplemental Fig. 1). In contrast, CSA does not cause HCM in wild type (WT) mice^36^. As a proof of concept, that the HCM phenotype arises without CSA; we compared the echo data of the aging 30 weeks old α-MHC^719/+^ mice with the young 12 weeks old α-MHC^719/+^ CSA treated mice. The aging mice echo data (Fig. 1) manifest that the phenotype arises in the 30 weeks old α-MHC^719/+^mice naturally and cyclosporine (CSA) treatment enhances the phenotype in the young 12 weeks old α-MHC^719/+^mice. In both cases this was accompanied with increased Hif-1α protein levels in left ventricular tissue (Fig. 2A,B and Supplemental Fig. 2), however no Hif-1α proteins level and no significant difference in the echo data between 12 weeks old WT mice treated with CSA and 30 weeks old WT mice without CSA treatment was detected, thus mice with induced HCM via CSA compared to CSA treated WT were used for the follow-up analyses. Beside the increase Hif-1α protein level after induction of HCM via CSA and compared to WT, enhanced levels of proteins classically upregulated in HCM such as myosin heavy chain 7 (Myh7) and desmin (Fig. 2A,B). In addition RNA expression of αMHC was reduced in α-MHC^719/+^ mice (Supplemental Fig. 3). To identify known and novel genes and proteins affected by increased Hif-1α protein expression, transcriptomics (Fig. 2C, Supplemental Table 5 and Supplemental Fig. 4) and proteomics (Fig. 2D and Supplemental Table 6) were performed. In total 152 genes and 380 proteins were higher and 419 genes and 177 proteins lower expressed in HCM mice compared to wildtype mice. Omics data were analyzed to identify HIF target genes dysregulated in α-MHC^719/+^ mice compared to WT mice. In the omics analysis 14 in α-MHC^719/+^ mice upregulated genes and 31 higher abundant proteins were identified as potential Hif-1α targets (Supplemental Tables 7 and 8).

### Generation of a cardiomyocyte-specific Hif-1a knockout α-MHC^719/+^ mouse line

**Fig. 3:**
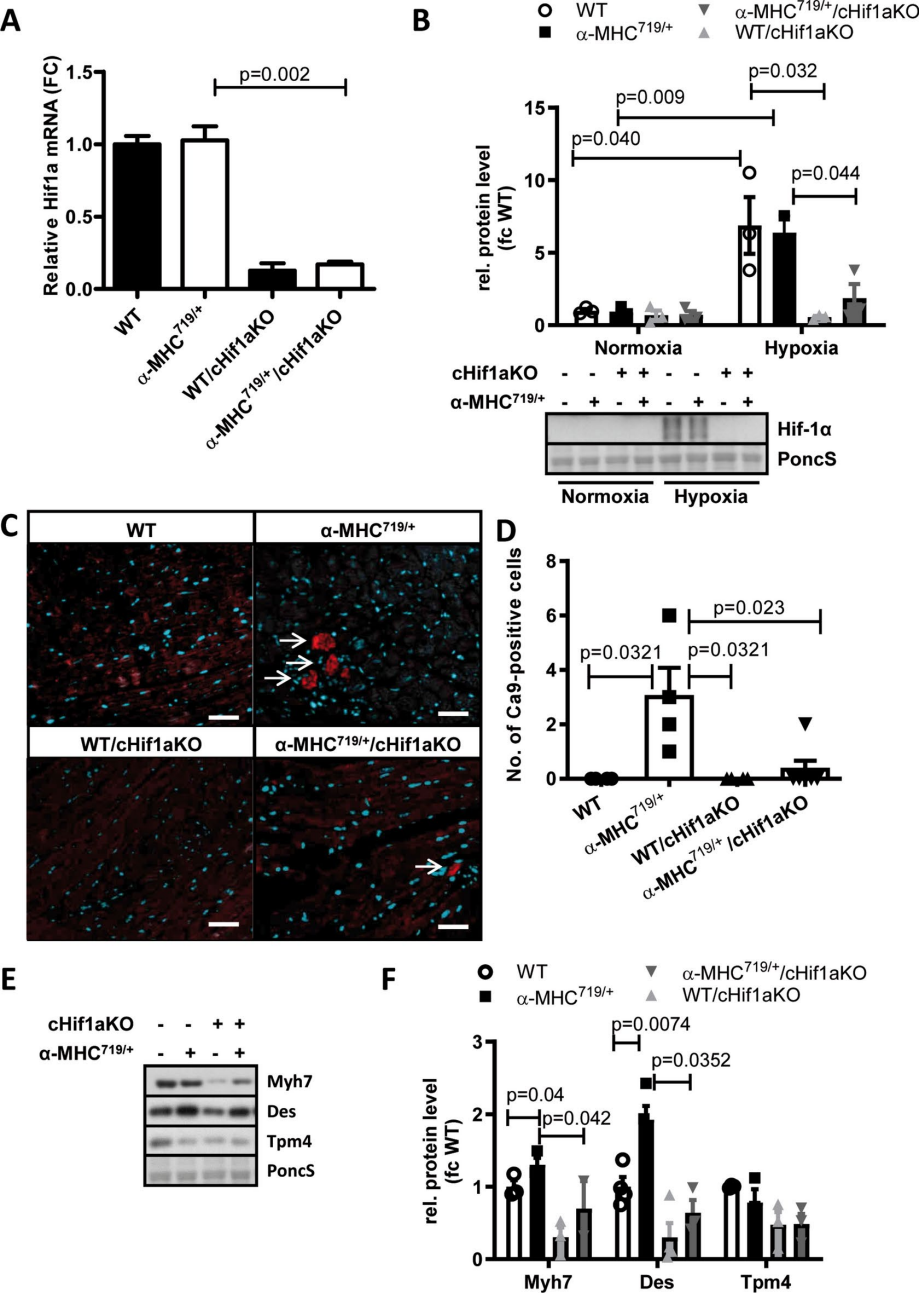
Cardiomyocyte specific Hif-1α knockout decreases HCM related protein expression. (**A**) Hif1a gene expression on RT-PCR was decreased in α-MHC^719/+^/cHif1aKO mice compared to α-MHC^719/+^. (**B**) Western blot from protein extracted of freshly isolated adult murine cardiomyocytes from WT, α-MHC^719/+^, WT/cHif1aKO, and α-MHC^719/+^/cHif1aKO (n = 3 in each group). Increased expression of Hif-1α on Western blot from protein extracted of freshly isolated adult murine cardiomyocytes after incubation under hypoxia conditions (1% oxygen) for 16 h in WT and α-MHC^719/+^ mice, but not in WT/cHif1aKO and α-MHC^719/+^/cHif1aKO mice (n = 3 in each group). Ponceau S (PoncS) served as loading control. (**C**,**D**) FFPE heart sections were stained with an antibody against CAIX. Nuclei were visualized with DAPI. (**C**) Representative staining is shown. (**D**) Average number of CAIX positive signal/ cells was measured in four high power fields per heart section of WT (n = 3), α-MHC^719/+^ (n = 5), WT/cHif1aKO (n = 4) and α-MHC^719/+^/cHif1aKO (n = 6) mice. Scatter plots with mean ± SEM with significances indicated by p values for all plots. Scale bar = 50 µm. (**E**,**F**) Western blot (**E**) and quantification (**F**) of classical HCM proteins Myh7. Desmin (Des) and Tpm4 from freshly isolated adult murine cardiomyocytes of WT, α-MHC^719/+^, WT/cHif1aKO and α-MHC^719/+^/cHif1aKO mice (n = 3 in each group). Ponceau S (PoncS) served as loading control. *fc* fold change. Data are presented as mean ± SEM with significances indicated by p values. Original western blot in (B) and (E) presented in (Supplemental Figs. 9, 10).

To investigate the role of Hif-1α in HCM disease pathogenesis, we crossbred a cardiomyocyte-specific Hif1a knockout mice line as previously described^35^ with α-MHC^719/+^ mice to generate α-MHC^719/+^/cHif1aKO mice. These crossbred mice were viable, fertile and showed no obvious phenotype or abnormal litter distribution compared to α-MHC^719/+^ mice (Supplemental Table 9). There was a decrease in Hif1a RNA expression in α-MHC^719/+^/cHif1aKO mice (Fig. 3A) and isolated cardiomyocytes of WT/cHif1aKO and α-MHC^719/+^/cHif1aKO mice showed no Hif-1 α protein induction compared to WT or α-MHC^719/+^ mice after hypoxia incubation at 1% oxygen for 16 h (Fig. 3B). As we do not see a change in Hif1a RNA levels in the mice, but only an increase on protein level this indicate a stabilization process due to hypoxia areas. To test for potential hypoxic regions we stained heart slides for Ca9, as in cancer well established surrogate marker for hypoxic areas^37^. Indeed Ca9-positive cells were detected in α-MHC^719/+^ mice (Fig. 3C,D) whereas almost no Ca9-positve cells could be found in WT/cHif1aKO or α-MHC^719/+^/cHif1aKO mice. Even under normoxic conditions cardiomyocytes from α-MHC^719/+^ showed upregulated classical HCM proteins like Myh7 or desmin and no change of these proteins were detected in cells from α-MHC^719/+^/cHif1aKO mice (Fig. 3E,F).

### Cardiomyocyte-specific Hif1a knockout in the α-MHC^719/+^ HCM mouse model attenuates disease phenotypes

**Fig. 4:**
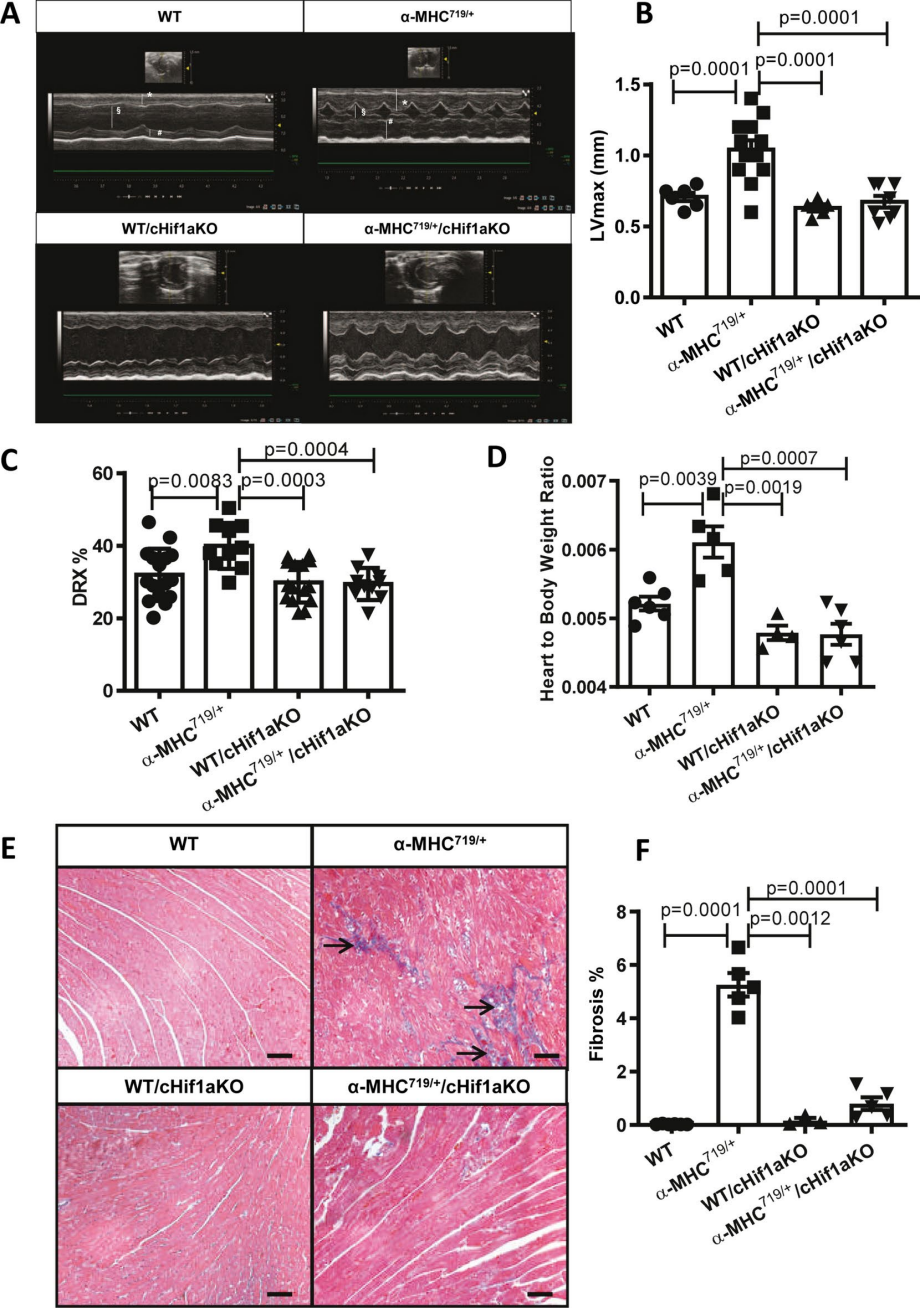
Cardiomyocyte-specific Hif-1α knockout in the α-MHC^719/+^ HCM mouse model attenuates disease phenotype. (**A**,**B**) Exemplary transthoracic echocardiographic imaging (**A**) and echocardiographic myocardial wall thickness measurements (**B**) of WT (n = 6), α-MHC^719/+^ (n = 13), WT/cHif1aKO (n = 5), and α-MHC^719/+^/cHif1aKO (n = 8) mice. Marked increase of the end-diastolic left ventricular septal (*) and posterior wall (#) thickness, and reduced end-diastolic LV diameter (§) in α-MHC^719/+^ compared to the WT mice on parasternal long axis view, which is ameliorated in α-MHC^719/+^/cHif1aKO mice. (**C**) Initial rapid decay amplitude corresponding to disordered relaxed state (DRX) heads conformations in LV myocardial tissue is abnormal in α-MHC^719/+^ (n = 5) but not in α-MHC^719/+^/cHif1aKO (n = 6) compared to WT (n = 6) and WT/cHif1aKO (n = 4) mice. (**D**) Cardiomyocyte-specific Hif1α knockout in the α-MHC^719/+^ HCM mouse model attenuates disease phenotype: Increased heart weight–body weight ratios in α-MHC^719/+^ (n = 5) but not in α-MHC^719/+^/cHif1aKO (n = 6) compared to WT (n = 6) and WT/cHif1aKO (n = 4) mice. (**E**,**F**) Myocardial fibrosis: Exemplary Masson trichrome staining of the myocardium in WT, α-MHC^719/+^, WT/cHif1aKO, and α-MHC^719/+^/cHif1aKO mice is shown. The magnification bar represents 100 μm (**E**). The area of fibrosis, stained in blue, was measured morphometrically in WT (n = 6), α-MHC^719/+^ (n = 5), WT/cHif1aKO (n = 4), and α-MHC^719/+^/cHif1aKO (n = 6) mice as a percentage of the entire myocardium (**F**). Myocardial fibrosis (blue), as indicated by arrows in E, markedly increased in α-MHC^719/+^ mice compared to WT. When compared to α-MHC^719/+^ mice, the myocardial fibrosis in α-MHC^719/+^/cHif1aKO mice were found to be less pronounced. *fc* fold change. Scatter plots show mean ± SEM, with significances indicated by p-values for all plots.

Next, we investigated the effect of the cardiomyocyte-specific Hif1a knockout in the HCM model of α-MHC^719/+^ mice. α-MHC^719/+^ mice displayed all pathognomonic features of HCM, like myocardial hypertrophy in transthoracic echocardiography (Fig. 4A,B, Supplement Fig. 6 and Supplement Table 1) and increased disordered relaxed state (DRX) of the myosin filaments (Fig. 4C) as previously described. Furthermore, higher heart to body weight ratio (Fig. 4D) and increased myocardial fibrosis on histopathology (Fig. 4E,F) was detected. In contrary, cardiomyocyte-specific Hif1a knock out mice did not show any left ventricular myocardial thickening in echocardiography, in the myosin head relaxation state and in the heart to body weight ratio (Fig. 4A–D). Compared to α-MHC^719/+^ mice, there was reduced cardiac fibrosis (Fig. 4E,F). Thus, cardiomyocyte-specific knockout of Hif-1α rescued the disease phenotypes in α-MHC^719/+^ mice.

### Cardiomyocyte specific Hif-1a knockout attenuates levels of HCM related genes and proteins

**Fig. 5:**
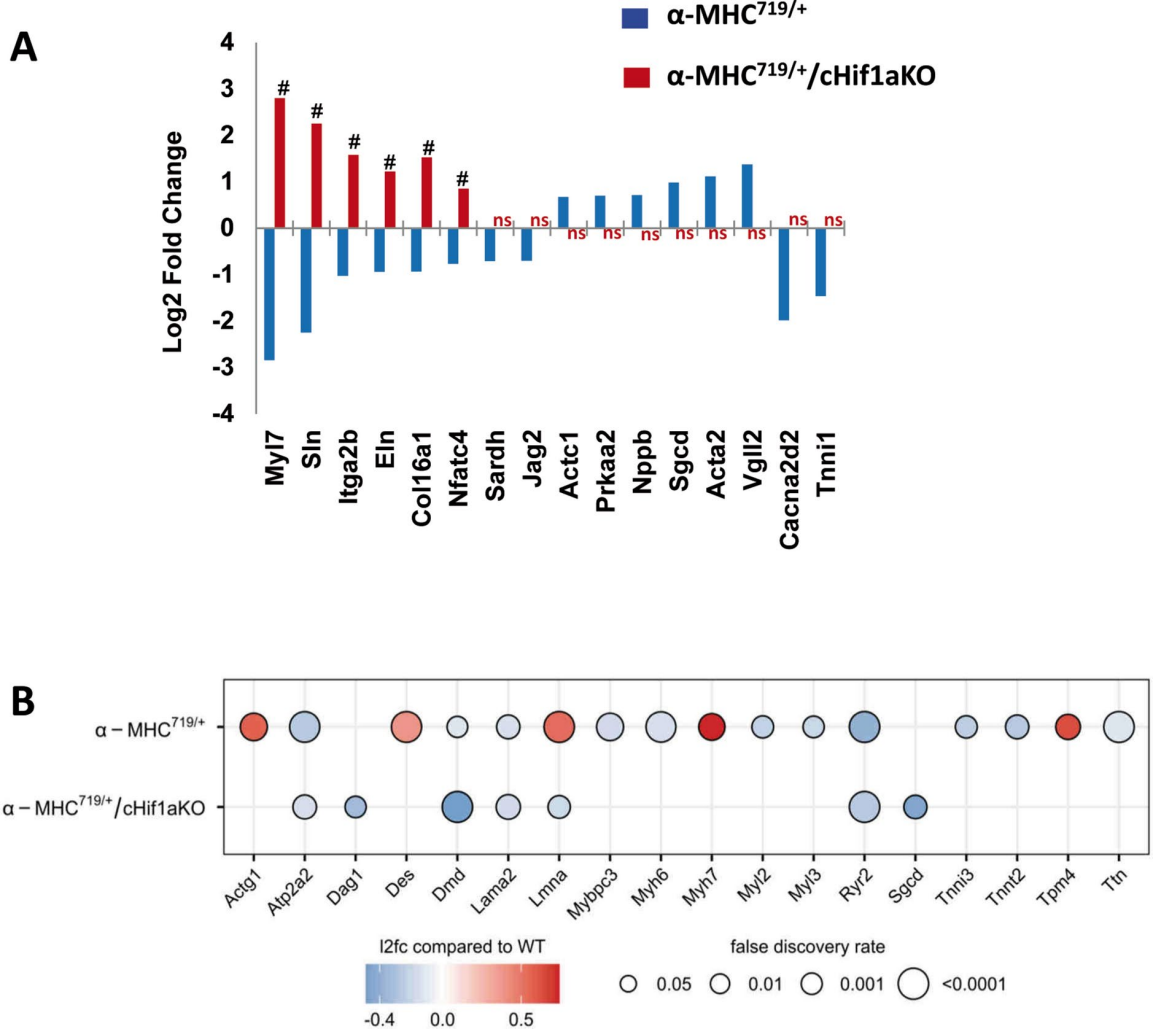
Normalization of proteins and signaling pathways involved in pathological myocardial remodeling on proteomic analysis of α-MHC^719/+^ mice lacking Hif-1α in cardiomyocytes. (**A**) Shown is a subset of genes known to be involved in pathologic myocardial remodeling and significantly dysregulated in α-MHC^719/+^ mice that show counter regulation or normalization towards WT levels in α-MHC^719/+^/cHif1aKO mice, represented as the log2 fold change relative to WT. # denotes q-value < 0.05; ns denotes non-significantly regulated. (**B**) Known pro-hypertrophic, pro-fibrotic and pro-inflammatory proteins dysregulated in α-MHC^719/+^ compared to WT mice are unchanged in α-MHC^719/+^/cHif1aKO compared to WT mice.

Several dysregulated HCM related gene and protein sets were revealed by transcriptome (Fig. 2C) and proteome analysis (Fig. 2D). However, in α-MHC^719/+^/cHif1aKO mice, they were counter regulated or unchanged (Fig. 5). Interestingly the LV transcriptome showed a concrete subset of 17 genes dysregulated in α-MHC^719/+^ mice of which the majority (16 genes) was either not significantly changed or reciprocally regulated in LV from α-MHC^719/+^/cHif1aKO mice (Table 1A and Fig. 5A). Proteomic analysis supported these findings (Table 1B and Fig. 5B). In α-MHC^719/+^/cHif1aKO mice more than 96% of 61 proteins dysregulated in α-MHC^719/+^ mice were unchanged or reciprocally regulated. The positive dysregulated proteins included known HCM proteins like gamma Actin (gene Actg1), Desmin (gene Des), Prelamin A/C (gene Lmna), Myosin heavy chain-7 (gene Myh7) and Tropomyosin alpha-4 chain (gene Tpm4), respectively (Fig. 5B). Indeed, in α-MHC^719/+^ mice Tpm4 and SOD3 were upregulated on protein level in left ventricular tissue (Fig. 2A,B and Supplemental Fig. 5).

**Table 1:** Reciprocal regulated Hif-1α targets upregulated in LV of α-MHC^719/+^ and downregulated in α-MHC^719/+^/cHif1aKO mice.

Gene symbol	α-MHC^719/+^ vs WT		α-MHC^719/+^/cHif1aKO vs α-MHC^719/+^	
	l2fc	p-value	l2fc	p-value
A - regulated on RNA level				
Ubxn10	1.205	1.52E-03	−1.014	0.01
Cenpf	1.016	7.66E-03		
Tfrc	0.989	3.85E-03		
Ovol1	0.968	3.32E-02		
Egln3	0.873	1.05E-02		
Arntl	0.872	3.45E-02		
Emc2	0.823	1.52E-02	−0.826	0.02
Pde3a	0.810	2.06E-02		
Ccl9	0.806	4.58E-02		
Klk1b22	0.803	3.98E-02	−1.272	0.00
Pggt1b	0.772	4.00E-02	−0.795	0.04
Rab21	0.750	2.77E-02		
Uba3	0.735	3.14E-02		
Prkaa2	0.697	3.81E-02		
B - regulated on protein level				
Vim	1.287	0.00E + 00	−1.122	0.00E + 00
Tppp3	1.148	2.84E-04		
Ltbp4	0.826	3.72E-03	−0.898	2.28E-02
Dpysl2	0.725	0.00E + 00	−0.754	2.22E-16
Akr1a1	0.689	4.61E-10	−0.564	2.05E-05
Rab10	0.581	9.37E-03	−0.574	4.50E-03
Sec31a	0.500	9.03E-03	−0.571	8.77E-03
Ruvbl2	0.485	1.63E-02		
Itih1	0.433	9.77E-03		
Tln1	0.432	0.00E + 00	−0.481	0.00E + 00
Calu	0.399	1.33E-02	−0.603	5.85E-06
Cast	0.388	1.74E-02		
Serpinh1	0.379	3.46E-04	−0.495	1.70E-06
Gnb2	0.373	3.91E-05	−0.239	1.37E-02
Cp	0.365	2.27E-06	−0.356	2.81E-05
Hnrnpa3	0.338	1.85E-02	−0.467	4.24E-04
Cand1	0.337	2.77E-02	−0.462	1.74E-04
Arpc2	0.336	4.56E-02	−0.502	2.65E-03
Rac1;Rac3;Rac2	0.335	3.38E-03	−0.447	1.44E-04
Mif	0.329	1.01E-02	−0.633	2.78E-06
Stip1	0.317	2.89E-05	−0.361	1.06E-05
Psma7	0.315	6.11E-03	−0.271	7.52E-06
Hk1	0.302	2.23E-09	−0.310	1.64E-09
Wdr1	0.300	1.40E-05	−0.396	3.50E-07
Mapre2	0.297	1.43E-02	−0.330	1.05E-02
Pabpc1	0.288	4.55E-02		
Uba1	0.235	5.37E-05	−0.271	7.52E-06
Csrp3	0.203	3.73E-02		
Gbe1	0.200	3.88E-02	−0.400	1.30E-03
Gapdh	0.195	6.80E-04	−0.143	6.38E-03
Sptbn1	0.129	3.02E-04	−0.200	4.19E-08

*n.s.* not significant.

l2fc = log2 fold change.

### Hif-1α contributes to disease pathogenesis of HCM via oxidative stress, inflammation and angiogenesis

**Fig. 6:**
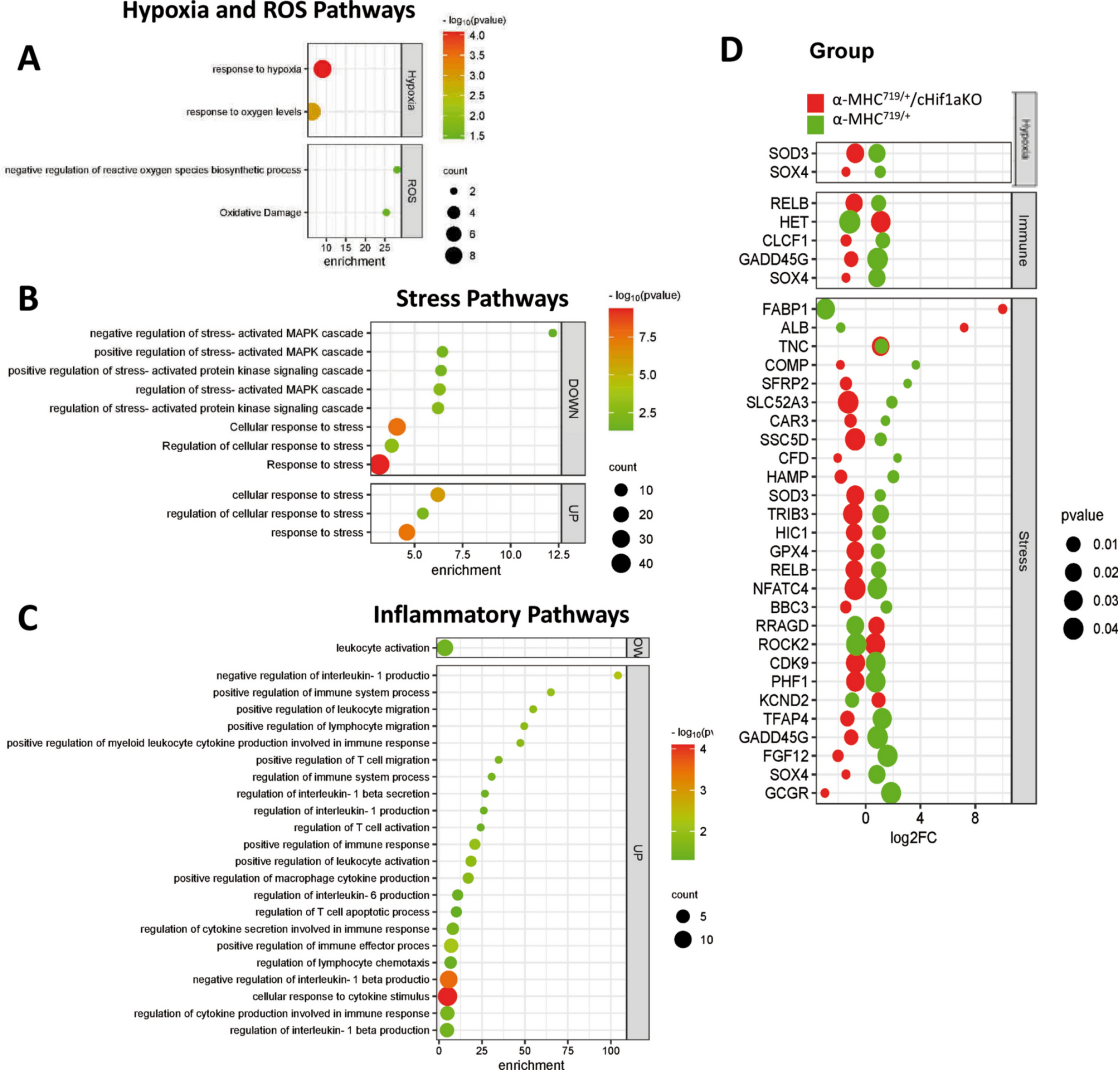
Normalization of potential mechanistic pathways involved in pathological myocardial remodeling on transcriptome analysis of α-MHC^719/+^ mice lacking Hif-1α in cardiomyocytes. (**A**–**C**) Enrichment bubble graph for hypoxia/ROS (**A**), stress (**B**) or inflammation (**C**) related pathways dysregulated in α-MHC^719/+^ mice. (**D**) Gene expression pattern of key genes involved in hypoxia (Hx), immune and stress related pathways dysregulated in in α-MHC^719/+^ (green) compared to WT mice are unchanged in α-MHC^719/+^/cHif1aKO (red) compared to WT mice.

**Fig. 7:**
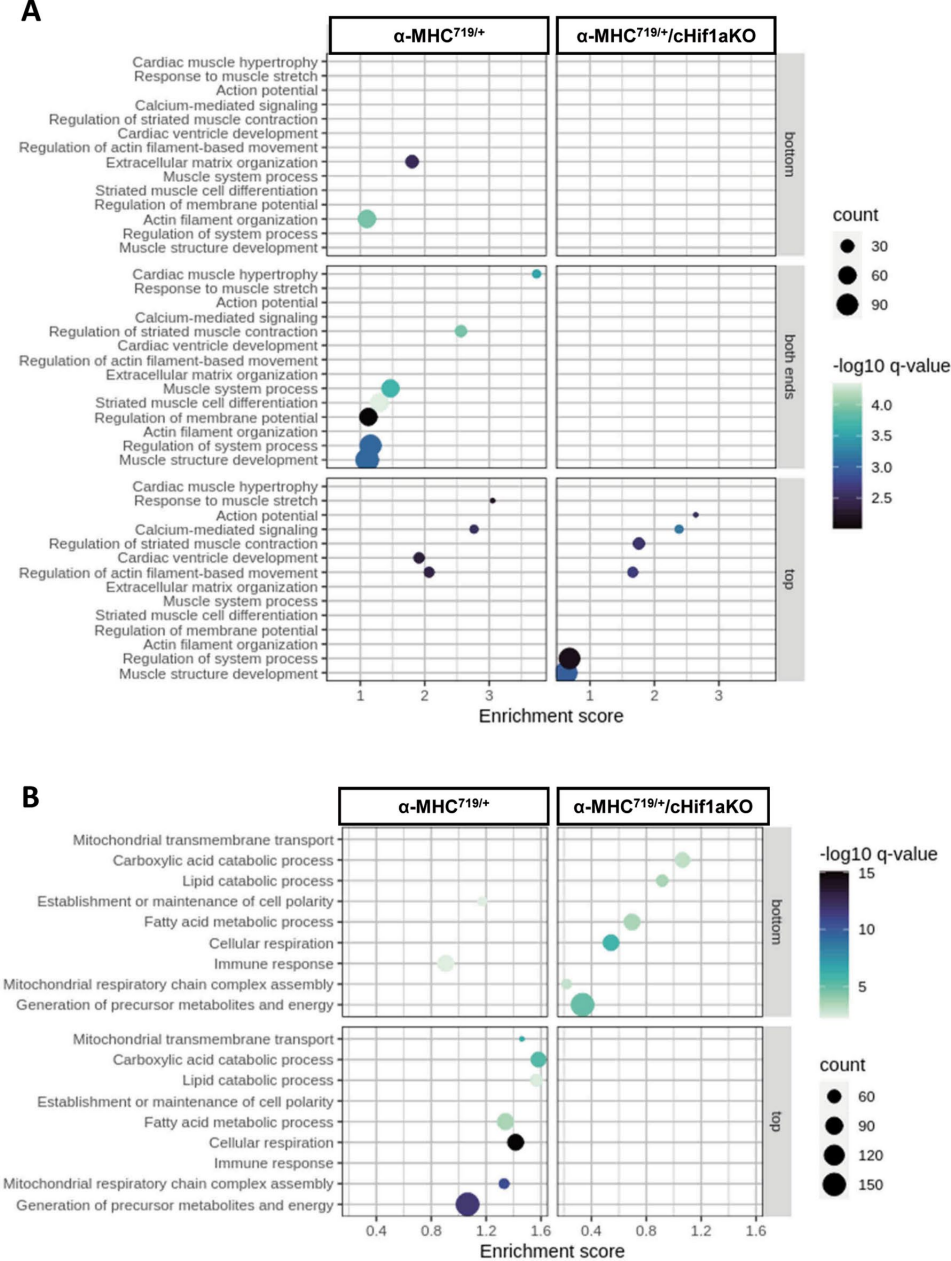
Pathways differential affected in the proteome of α-MHC^719/+^ mice and α-MHC^719/+^ mice lacking Hif-1α in cardiomyocytes. Enrichment bubble plot for cardiac related pathways (**A**) or potential mechanistic related pathways (**B**) in the hearts of α-MHC^719/+^ mice or α-MHC^719/+^/cHif1aKO mice.

To further analyze the mechanism how Hif-1α affects the pathogenesis of HCM differential pathway analysis was performed. Pathways of hypoxia, general and oxidative stress, as well as inflammation were found to be dysregulated in α-MHC^719/+^ mice (Fig. 6A–C and Supplemental Table 10). Genes involved in these processes were reversed regulated in α-MHC^719/+^/ cHif1aKO mice (Fig. 6D). In the proteome analysis, many cardiac and mechanistically related pathways were enriched among the altered proteins in α-MHC^719/+^ mice, but not in α-MHC^719/+^/ cHif1aKO mice (Fig. 7A,B).

**Fig. 8:**
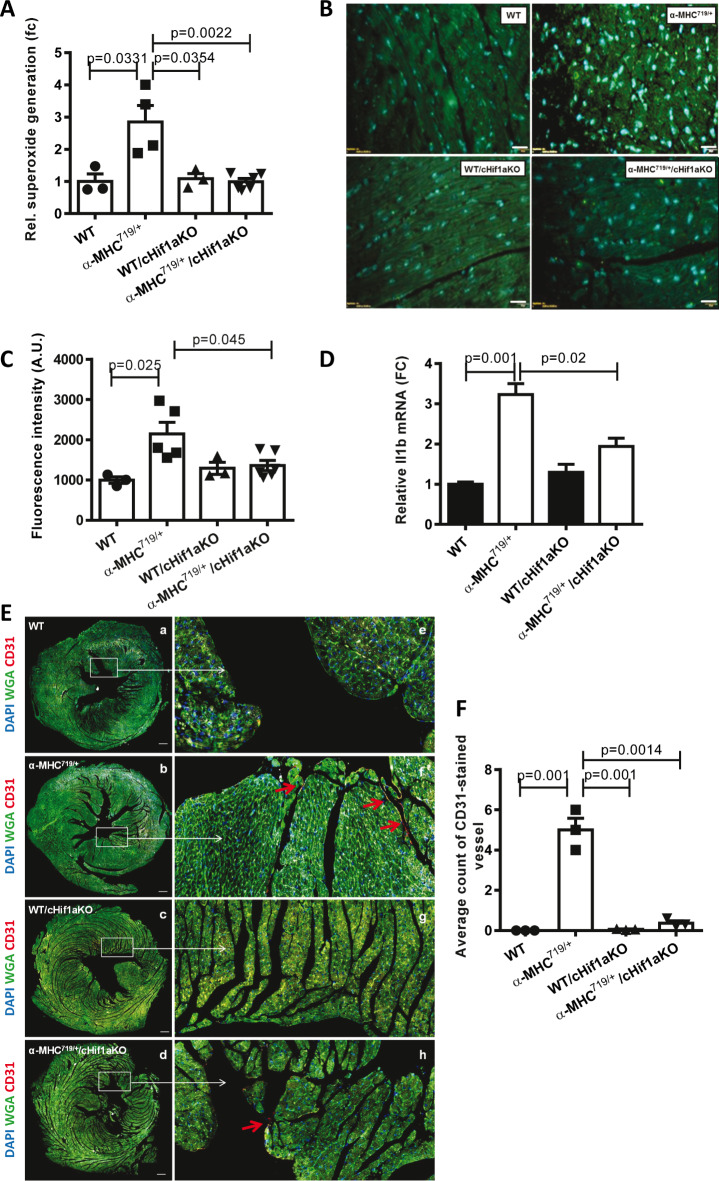
α-MHC^719/+^ mice showed oxidative stress, inflammation and dysregulated angiogenesis reversed in α-MHC^719/+^ mice lacking Hif-1α in cardiomyocytes. (**A**) Superoxide generation rate was measured by electron paramagnetic resonance using 1-Hydroxy-3-methoxycarbonyl-2, 2, 5, 5-tetramethylpyrrolidine in macerated LV tissue from α-MHC^719/+^ (n = 5), α-MHC^719/+^/cHif1aKO (n = 6), WT (n = 3) and WT/cHif1aKO mice (n = 4). (**B**,**C**) FFPE heart sections were stained with an antibody against 8-Hydroxydeoxyguanosine (8OHdG). Nuclei were visualized with DAPI. (**B**) Representative staining is shown. (**C**) Fluorescence intensity of 60 nuclei was measured in four high power fields per heart section (n = 3–6). (**D**) Il1b gene expression on RT-PCR is decreased in α-MHC^719/+^/cHif1aKO mice compared to α-MHC^719/+^. (**E**,**F**) Representative cardiac sections of HCM and cardiomyocyte-specific Hif-1α knock out mice line exhibiting cardiomyocytes in green, CD31 – stained vessels visualized in red by antibody staining. (**a**,**e**) WT, (**b**,**f**) α-MHC^719/+^, (**c**,**g**) WT/cHif1aKO and (**d**,**h**) α-MHC^719/+^/cHif1aKO mice line (n = 3–4 each group). Scale bar: 100 µm (**a**–**d**). Zoom in on CD31 stained Endothelial cells 50 µm (**e**–**h**). Scatter plots show mean ± SEM, with significances indicated by p-values for all plots.

Elevated level of oxidative stress in α-MHC^719/+^ mice but not in α-MHC^719/+^/cHif1aKO mice was detected via electron paramagnetic resonance (EPR) and 8-Hydroxydeoxyguanosine* (*8OHdG) staining (Fig. 8A–C). Also the inflammatory marker Il1b was enhanced in α-MHC^719/+^ and reduced in α-MHC^719/+^/cHif1aKO (Fig. 8D). Dysregulated angiogenesis as shown by CD31 staining was observed in α-MHC^719/+^mice, but not in α-MHC^719/+^/cHif1aKO mice (Fig. 8E,F). These data indicate that oxidative stress subsequently an inflammatory response as well as a dysregulated vascularization could be the mechanisms by which Hif-1α contributes to disease pathogenesis of HCM.

### Proteomic evaluation of Hif-1α targets in human serum samples from patients with early onset HCM

To translate findings from the HCM mouse model to the clinic, a proteomic analysis was performed with serum samples from 16 patients suffering from early onset HCM compared to age- and gender-matched 15 healthy controls (Table 2). In HCM patients, we identified the Hif-1α targets insulin like growth factor 2 (IGF2), Latent-transforming growth factor beta-binding protein 1 (LTBP1), Reversion-inducing-cysteine-rich protein with kazal motifs (RECK) as significantly upregulated whereas WD repeat-containing protein 1** (**WDR1) was downregulated (p < 0.05, Table 2) indicating a potential involvement of Hif-1α even in patients with HCM.

**Table 2:** Hif-1α targets found in HCM patients secretome.

Gene symbol	Uniprot ID	l2fc	p-value
IGF2	P01344	2.86	0.045
LTBP1	Q14766	2.14	0.032
RECK	O95980	2.10	0.012
WDR1	O75083	–2.32	0.025

l2fc = log_2_ fold change.

## Discussion

The data of this study provide evidence that HIF signaling is involved in human and mouse HCM, as shown on α-MHC^719/+^ mouse LV tissue and human serum analysis, respectively. Furthermore, cardiac specific Hif-1α knockout is able to reverse pathological myocardial remodeling in the α-MHC^719/+^ mouse model as evidenced by decreased ventricular hypertrophy, myocardial fibrosis, diastolic dysfunction, oxidative stress, and cardiomyopathic RNA and protein profiles. α-MHC^719/+^ mice showed distinguished gene and protein expression patterns which are normalized in the α-MHC^719/+^/cHif1aKO mice. These transcriptome and proteome profiles affect HCM related pathways including the positive dysregulation of HCM genes such as Myh7 and Des which could be identified to be dysregulated already in not phenotypic α-MHC^719/+^ mice but normalized in α-MHC^719/+^/cHif1aKO mice. In addition, mechanistic pathways related to oxidative stress, hypoxia with altered SOD3 expression were identified to be dysregulated in α-MHC^719/+^ mice. Also inflammation and angiogenesis related pathways were changed in α-MHC^719/+^ mice but normalized in α-MHC^719/+^/cHif1aKO mice. Further studies which were beyond the scope of this study will be needed to further explore the involvement of the identified pathways and their proteins and role of Hif-1α.

The observation of HIF involvement in HCM hypothesis is in line with other studies in patients with HCM^26,38^ and animal models^39^. A study in children and adolescents with HCM particularly showed an association between Hif-1α activating SNPs and increased myocardial hypertrophy and diastolic dysfunction^40^.

Hif-1α is a transcription factor induced by cellular response to hypoxia^41^ and a regulator of glucose metabolism, cell survival, oxidative stress, proliferation, angiogenesis, energy metabolism, and erythropoiesis^41,42^. Increased Hif-1α levels detected in left ventricular tissue of HCM mice (both cyclosporine treated and not treated) in the current study clearly indicates that Hif-1α is activated. The presence of Ca9 positive areas and the increased angiogenesis in the left ventricle is a clear indicator of Hif-1α activity. To our knowledge involvement of Hif-1α in the development of HCM has not yet been reported in this established HCM mouse model or in human serum samples.

Hif-1α plays an important role in the cardio protection in different cardiac diseases, e.g. ischemia heart disease or pressure overload heart failure^23^, pathologies which can be consequences of HCM. Hif-1α has been shown to play an important role in the protection of cardiomyocytes against apoptosis in a model of myocardiac infarction, because Hif-1α regulates glycolysis via AKT and PFKFB2 thus having an anti-apoptotic effect on cardiomyocytes^43^. In fibroblasts Hif-1α is known to control the formation of fibrosis after ischemia, as fibroblast specific knockdown leads to increased mitochondria derived ROS and increased fibroblasts proliferation and subsequently fibrosis^44^. In a pathophysiological setting, an increased Hif-1α expression seems to be beneficial, as in a murine model of diabetes the cardiac specific overexpression of Hif-1α protects against diabetic induced cardiomyocyte hypertrophy and fibrosis^45^. Cardiomyocyte specific Hif-1α overexpression during acute myocardiac infarction could prevent cardiomyocyte apoptosis via upregulating of HO-1 and downregulating of the pro-apoptotic BNIP1^46^ further supporting an anti-apoptotic role of Hif-1α. On the other hand, a cardiac overexpression of Hif-1α under a non-pathological condition mediates beneficial effects on the heart on the short term, but chronic overexpression leads to cardiomyopathy in the aging mice. A possible explanation is given by the finding that Hif-1α overexpression leads to increased apoptosis via p53 activation in isolated rat cardiomyocytes^47^. In addition, patients with dilated cardiomyopathy showed increased cardiac Hif-1α levels^48^ and endothelial decreased PHD2 expression in patients with different types of cardiomyopathy^49^. Heterozygote Hif-1α-KO mice with a global reduced Hif-1α level showed a lower risk for cardiac rupture and higher survival rates in a myocardium infarct model, although no difference in the infarct size itself was observed^47^. These findings are consistent with our observation that cardiac specific Hif-1α KO is protective against the development of HCM underlining the important role of Hif-1α in the development of different types of cardiomyopathy. However, also different findings are reported as a cardiac specific Hif-1α knockout leads to increased cardiac damage in an ischemia/reperfusion injury mouse model^35^. In addition, in a normoxic mouse model, cardiac specific knock out of Hif-1α leads already to an altered vascularization, energy availability, calcium flux, and contractility^50^. An involvement of Hif-1α in these processes was underlined with our proteomics analysis as pathways involved in fiber organization and contractility were altered in HCM mice, but not affected in the cardiac Hif-1α-KO mice. Hif-1α protein was not found in the proteome analysis, probably due to its instability under normoxic conditions during the processing of the sample. The role of Hif-1α in the development of cardiomyopathies seems to be cell type specific and HIF isotype specific, because endothelium expressed Hif-1α seems to have a protective role in the development of cardiomyopathies. Endothelium specific Hif-1α knockout aggravates cardiac hypertrophy and increase the LV/body mass ratio^24,49^, whereas endothelium specific Hif-2α knockout was protective. However, our data provide evidence that the cardiomyocyte specific Hif-1α knock out is protective against the development of the phenotype of HCM. This finding is supported by other studies which detected also an upregulated Hif-1α in HCM^51^. Interestingly, in this study Hif-2α was downregulated and restoring Hif-2α expression which was associated with a downregulation of Hif-1α showed a protective effect. Although involvement of Hif-1α was not investigated mechanistically, this observation is in line with our finding that downregulation of Hif-1α is beneficial in cardiomyopathies. Indeed, evidence for altered Hif-1α downstream target IGF2 protein expression in serum samples of patients with early onset severe disease phenotype was found indicating that secreted Hif-1α targets may be a marker for the early detection of HCM.

As a future aspect, the role of Hif-1α may be further explored using iPS-cells derived cardiomyocytes (iPS-CMCs) using iPS cells of HCM patients. Similar studies were already performed using iPS-CMCs from patients with Noonan syndrome with a PTPN11^N308S/+^ mutation confirming cell cycle defects putative driving the phenotype of the Noonan syndrome^52^. iPS cells derived from HCM patients carrying mutations in the TNNI3 gene are already existing^53^ and can be used to further investigating the role of Hif-1α using Hif-1α knock down approaches in future studies.

## Methods and material

### Chemicals and general

All chemicals were from Sigma-Aldrich unless stated differently. Electronic laboratory notebook was not used.

### Mouse models

#### Hypertrophic cardiomyopathy mouse model

All animal procedures were performed in accordance with the Directive 2010/63/EU of the European Parliament and approved (Project Number: 55.2–1-54-2532-242-2015) by the local legislation on protection of animals (Government of Upper Bavaria, Munich, Germany), and the study is reported in accordance with the ARRIVE guidelines.

For the experiments, the previously described R719W-129SvEv mice (designated as α-MHC^719/+^) were used. This mouse line contain an Arg719Trp (R719W) mutation in the murine α cardiac MHC gene. The mice were generated in-house by Prof. Schmitt at the University of Düsseldorf (Germany) using the 129SvEv mouse line from the Harvard University (USA) by homologous recombination with a targeting construct containing an EcoR1 fragment derived from the murine α cardiac MHC gene with the human HCM mutation Arg719Trp as described^8^. This mutation leads to the development of typical pathognomonic features of hypertrophic cardiomyopathy in old age^8^. For experiments heterozygous α-MHC^719/+^ mice were used and compared with age- and sex-matched wild types of the same background. Several studies have shown that young male alpha MHC mice showed more evidence of disease than their female counterparts^6,54^, hence we used only male heterozygous α-MHC^719/+^ mice and male Wild type mice similar to the previous study^8^. Based on sample size used in the previous studies^8,55^, sample size calculation and taking the 3R principle for animal experiments (refinement, reduction, replacement) into considerations, the final number of animals were 6 wildtype and 5 α-MHC^719/+^ mice.

#### Generation of α-MHC^719/+^/cHIF1aKO mice

A cardiomyocyte-specific Hif1a knock out line (cHif1aKO) was generated by crossing a LoxP flanked Hif1a mouse line (Jackson Lab, Bar Harbor, Maine, USA; stock no. 011038) with mice expressing the Cre-recombinase driven by the α-myosin heavy chain promoter (Jackson Lab stock no. 007561).

The cHif1aKO mice were bred with HCM mice to generate α-MHC^719/+^/cHif1aKO mice. Only male Cre positive α-MHC^719/+^/cHif1aKO and Cre negative α-MHC^719/+^/cHif1aKO mice were used for experiments. Based on the previous study^8^ and sample size calculation, the initial number of animals was 6 cHif1aKO and 6 α-MHC^719/+^/ cHif1aKO mice and the final number of animals 4 cHif1aKO and 6 α-MHC^719/+^/ cHif1aKO mice.

#### Administration of cyclosporin A

Acceleration of phenotype was already described ^8^. In short, hypertrophic remodeling was accelerated in α-MHC^719/+^ and α-MHC^719/+^/cHif1aKO mice (age 6–12 weeks) by subcutaneous injection of cyclosporin (CsA) (15 mg/kg body weight in PBS, twice daily) for 6 weeks. For all CsA studies, control mice were age-matched, male CsA treated WT and age-matched, male CsA treated WT/cHif1aKO mice.

#### Euthanasia

At the end of the experiment, the test animals were painlessly euthanized by cervical dislocation under isoflurane anesthesia. This took place at the German Heart Center in Munich as approved by the local legislation on protection of animals (Government of Upper Bavaria, Munich, Germany).

### Echocardiography

Transthoracic echocardiography was performed using a SONOS 4500 echocardiograph (Hewlett Packard) by using a 6 to 15 MHz linear-array probe and the Vevo lab 2100 Imaging system (Visualsonics) as described previously^56^ on sedated mice. The images were taken in 2D (left parasternal long and short axis) and M-Mode (left parasternal short axis). Measurements of left parasternal long and short axes and M-mode (left parasternal short axis) images were obtained at a heart rate of 500–550 bpm as described previously. LV end-diastolic diameter (LVEDD) and LV end-systolic diameter (LVESD), and wall thickness were measured from M-mode tracings, and the average of three consecutive cardiac cycles is reported. The LV fractional shortening percentage was calculated as ((LVEDD-LVESD)/LVEDD) × 100. Heart rate (bpm), left ventricular end-diastolic myocardial thickness, left ventricle end-diastolic diameter, left ventricle end-systolic diameter and ejection fraction %, were calculated in accordance with the American Society of Echocardiography guidelines as previously reported^57^.

### Heart weight to body weight ratio

The animals were placed individually in an induction chamber, and anesthesia was induced with 3% isoflurane (Harvard Apparatus Isoflurane Anesthetic Vaporizers) in 100% oxygen with a delivery rate of 5 l/min until loss of righting reflex. Upon reaching surgical plane, hearts were removed after performing midsection thoracotomy and rinsed in 4 °C phosphate buffer saline (PBS: 140 mM NaCl, 3 mM KCl, 6.5 mM Na_2_HPO_4_, 1.5 mM KH_2_PO_4_, pH 7.4). Ventricles were separated from atria and blood vessels, blotted dry and the ventricular weight was measured. Heart weight to body weight ratio (HW/BW) was then calculated by dividing the weight of the ventricles by the weight of the whole animal.

### RNA extraction, reverse transcription, and quantitative real-time PCR (RT-qPCR)

RNA was isolated from mouse tissue using peqGOLD Total RNA Kit (Peqlab, Erlangen, Germany) according to the manufacturer’s instruction. First-strand cDNA was synthesized using 500 ng of total RNA which was reversed-transcribed using a reverse transcriptase Kit (ThermoFisher Scientific, Karlsruhe, Germany). Real-time PCR analysis was performed using the Perfecta SYBR Green fast Mix (VWR, Darmstadt, Germany) in a 6000 (Corbett Life Science, Australia). Quantification was performed using ∆CT calculation and randomization test^58^. The following primers were used:

**Table:** 

Gene	Forward primer	Reverse primer
Hif1a	5'-GCAGTTAAGAGCACTAGTTG-3'	5'-GGAGCTATCTCTCTAGACC-3'
Actb	5'-CTAAGGCCAACCGTGAAAAG-3'	5'-ACAGCCTGGATGGCTACG-3'
Il1b	5'-TGCCACCTTTTGACAGTGATG-3'	5'-TGATGTGCTGCTGCGAGATT-3'
αMhc	5'-TGAAAACGGAAAGACGGTGA-3'	5'-TCCTTGAGGTTGTACAGCACA-3'

### Mant-ATP assay

To calculate the proportion of myosin heads in DRX and super relaxed state (SRX) can be calculated by using the rate of ATP cycling in relaxed muscle, which can be measured by the decay of a fluorescent, non-hydrolysable ATP (Mant-ATP) from cell fibers^59^. Mant-ATP assays were performed on snap frozen mice myectomy samples prepared as previously described^60^ to calculate the DRX to super relaxed state (SRX) ratio^61^. Mant-ATP assays were performed on snap frozen mice myectomy samples prepared as previously described. Briefly, tissue samples were thawed and permeabilized in permeabilization buffer consisting of 100 mM NaCl, 8 mM MgCl_2_, 5 mM EGTA, 5 mM K_2_HPO_4_, 5 mM KH_2_PO_4_, 3 mM NaN_3_, 5 mM ATP, 1 mM dithiothreitol (DTT), 20 mM 2,3-butanedione monoxime (BDM), and 0.1% Triton X100 (pH 7.0). Once samples were permeabilized, they were placed in a glycerinating solution consisting of 120 mM K acetate, 5 mM Mg acetate, 2.5 mM K2HPO4, 2.5 mM KH2PO4, 50 mM MOPS, 5 mM ATP, 20 mM BDM, 2 mM DTT, and 50% (v/v) glycerol (pH 6.8) to allow storage (-20 °C for 2 days) or immediate use. Before fluorescence acquisition, sections of tissue were pinned in a flow chamber constructed out of a microscope slide and coverslip and each chamber was flushed with an ATP buffer consisting of 120 mM CH_3_CO_2_K, 5 mM Mg(CH₃COO)₂, 2.5 mM K_2_HPO_4_, 2.5 mM KH_2_PO_4_, 4 mM ATP, 50 mM MOPS, and 2 mM DTT (pH 6.8) to remove glycerol. This buffer was replaced with rigor buffer consisting of 120 mM CH_3_CO_2_K, 5 mM Mg(CH₃COO)₂, 2.5 mM K_2_HPO_4_, 2.5 mM KH_2_PO_4_, 50 mM MOPS, and 2 mM DTT (pH 6.8). For fluorescence acquisition, a Nikon TE2000-E was used with a Nikon 20x/0.45 NA objective using a Hamamatsu C9100 electron multiplying charge-coupled device. Frames were acquired every 10 s with a 20-ms acquisition and exposure time using a 4’, 6-diamidino-2-phenylindole filter set. Initial fluorescence acquisition was simultaneous with the addition of rigor buffer containing 250 μm Mant-ATP to visualize wash in of fluorescent Mant-ATP. At the end of a 15-min acquisition, ATP buffer (rigor buffer and 4 mM ATP) was added to the chamber with simultaneous acquisition of the Mant-ATP chase. After experimental procedures, three regions of each chamber were sampled for fluorescence decay analyses in ImageJ (National Institutes of Health, Bethesda, MD) as previously described^60^.

### Isolation and cultivation of adult murine cardiomyocytes

Adult murine cardiomyocytes were isolated as described^62^. In total, cardiomyocytes from 4 animals from each genotype were isolated. Briefly, murine hearts were washed and perfused with EDTA buffer (130 mM NaCl, 5 mM KCl, 0.5 mM NaH_2_PO_4_, 10 mM HEPES, 10 mM glucose, 10 mM 2, 3-butanedione 2-monoxime, 10 mM taurine, and 5 mM EDTA), followed by perfusion buffer (130 mM NaCl, 5 mM KCl, 0.5 mM NaH_2_PO_4_, 10 mM HEPES, 10 mM glucose, 10 mM 2,3-butanedione 2-monoxime, 10 mM taurine, and 1 mM MgCl_2_). Hearts were digested with collagenase digestion mix (0.5 mg/ml collagenase 2, 0.5 mg/ml collagenase 4, and 0.05 mg/ml protease XIV). After digestion, perfusion buffer containing 5% FCS was added to the suspension to stop further enzymatic reactions. The suspension containing myocytes was passed through a 100-μm pore-size strainer in order to remove undigested tissue debris. After sequential gravity settlement, myocytes were cultivated in DMEM medium (PAN Biotech) supplemented with 10% fetal calf serum (PAA), 100 U/ml penicillin (Gibco), 100 μg/ml streptomycin (Gibco) and 2, 3-butanedione monoxime to prevent myocyte contractions. Cells were incubated at 37 °C under humidified atmosphere either at ambient oxygen concentration and 5% CO_2_ or at 1% oxygen and 5% CO_2_ concentration in a hypoxic chamber (InVivo2, I&L Biosystems, Königswinter, Germany).

### Immunoblot analysis

Total proteins, isolated from murine LV myocardial tissue were isolated using 1 × Cell lysis buffer which was generated by diluting 10 × Cell Lysis Buffer (#9803, Cell Signaling Technologies, Frankfurt, Germany) in ddH_2_O supplemented with 1 × complete Mini Protease-Inhibitor Cocktail (Roche, Penzberg, Germany). Proteins were separated by 8–12% SDS polyacrylamide gel electrophoresis using Mini-Protean 3 system (BioRad, Feldkirchen, Germany), transferred to nitro-cellulose membranes (Cytiva, Freiburg, Germany) and incubated with the following primary antibodies: Hif-1α (#36,169, Cell Signaling Technologies), Desmin (#5332, Cell Signaling Technologies), MYH7 (SAB2106550-100U, Sigma Aldrich, Heidelberg), TPM4 (#PA5-92,152, ThermoFisher Scientific, Darmstadt, Germany), SOD3(#S4946, Merck-Millipore, Darmstadt, Germany). Western blot were quantified using ImageJ (version 1.50i)^63^. Normalization was performed by total protein normalization using Ponceau S staining as recommended in the literature^64^. Uncropped western blot images included in the supplemental information file (Supplemental Figs. 8, 9, 10, 11, 12). Membranes were cut in accordance to the predicted protein size prior to antibody incubation (Supplemental Figs. 13). Two-tailored student’s test was used for statistical evaluation. Data are presented as a mean ± standard error.

### Immunohistochemistry

For immunostaining, deparaffined 2 µm FFPE sections of murine hearts were used. After heat-induced antigen retrieval (pH 9.0 for 20 min), sections were blocked in 3% BSA in PBS. Sections were incubated with primary antibody against Ca9 (#NB100-417, Novus/Bio-Techne, Wiesbaden, Germany) or 8-OHdG (#NB600-1508, Novus/Bio-Techne, Wiesbaden, Germany) or CD31 (#77,699, Cell Signaling) overnight at 4 °C. After washing, secondary goat anti-mouse antibody conjugated with Alexa Fluor 488 or Alexa Fluor 555 (ThermoFisher Scientific) together with wheat-germ agglutinin for membrane staining (Biomol, Hamburg, Germany) was applied for 1 h at room temperature in a dark chamber. Sections were counterstained with Hoechst 33,342 (ThermoFisher Scientific) for 1 min and mounted with anti-fade mounting medium (DAKO, Hamburg, Germany). Pictures were taken using a fluorescence microscope (OLYMPUS, Hamburg, Germany) and analyzed with cellSens Dimension imaging software in version 2.1 (OLYMPUS). Alternatively, slides were scanned with a fluorescence slide scanner (Axioscan 7, Zeiss, Oberkochen, Germany) using the ZEN software in version 2.3 (Zeiss). Numbers of Ca9 positive cells were counted for at least 4 hearts per group. Positive CD31 signal expression density in the vascular endothelium cells in each hearts for at least 4 hearts per group were counted. For quantification of 8-OHdG staining, fluorescence intensity was measured in at least 30 nuclei per heart for at least 4 hearts per group.

### Measurement of superoxide anion generation by EPR

Superoxide anion detection via EPR was performed as described^65^. Briefly, LV tissues were washed once in PBS and macerated in Krebs–HEPES buffer (NaCl 99 mM, KCl 4.69 mM, NaHCO_3_ 25 mM, KH_2_PO_4_ 1.03 mM, d-glucose 5.6 mM, Na-HEPES 20 mM, CaCl_2_ 2.5 mM, MgSO_4_ 1.2 mM) supplemented with 5 μM diethyldithiocarbamate (DETC) and 25 μM desferroxamine (DES). After adding 100 μM superoxide-specific spin probe 1-hydroxy-methoxycarbonyl-2,2,5,5-tetramethyl-pyrrolidine hydrochloride (Noxygen, Elzach, Germany), tissue suspension was measured in an EPR spectrometer (Bruker, Ettlingen, Germany) with an attached temperature controller (Noxygen). EPR settings for CMH spin label were center field 3460 G and auto tuning setting for sweep width, frequency, and microwave power and modulation amplitude. Spectra were recorded over 7 min.

### RNA sequencing

Strand-specific, polyA-enriched RNA sequencing was performed as previously described^66^. Briefly, RNA was isolated from whole-cell lysates using the AllPrep RNA Kit (Qiagen) and RNA integrity number (RIN) was determined with the Agilent 2100 BioAnalyzer (RNA 6000 Nano Kit, Agilent) and a RIN of 8 or higher was set as cutoff. For library preparation, one μg of RNA was poly (A) selected, fragmented, and reverse transcribed with the Elute, Prime, and Fragment Mix (Illumina). A-tailing, adaptor ligation, and library enrichment were performed as described in the TruSeq Stranded mRNA Sample Prep Guide (Illumina). RNA libraries were assessed for quality and quantity with the Agilent 2100 BioAnalyzer and the Quant-iT PicoGreen dsDNA Assay Kit (Life Technologies). RNA libraries were sequenced as 100 bp paired-end runs on an Illumina HiSeq4000 platform. The STAR aligner (version 2.4.2a)^67^ with modified parameter settings (–twopassMode = Basic) was used for split-read alignment against the mouse genome assembly mm39 (GRCm39) and UCSC known gene annotation. To quantify the number of reads mapping to annotated genes we used HTseq-count (version 0.6.0)^68^. FPKM (fragments per kilobase of transcript per million fragments mapped) values were calculated using custom scripts. Differential expression analysis was performed using the R Bioconductor package DESeq2^69^. For pathway and gene set enrichment analysis we used the R Bioconductor packages GAGE^70^ plus pathview^71^ and GOseq^72^, respectively. All gene set files for this analysis were obtained from GSEA algorithm implemented in R software^73^. Alternatively, the GeneTrail 3 web tool was used in ORA setting^74^. Enrichment score (ES) and False discovery rate (FDR) value were applied to sort pathways enriched. Furthermore, to assess statistical significance, we randomized our data set by permuting gene sets 1000 times and considered only gene sets with a p-value ≤ 0.05.

The RNAseq data in this publication have been deposited in NCBI’s Gene Expression Omnibus^75^ with the accession number GSE266649.

### Bioinformatic databases

For identification of HCM related genes, a gene set derived from the Molecular Signatures Database v7.5.1^76^, datasets “KEGG_HYPERTROPHIC_CARDIOMYOPATHY_HCM” and “HP_HYPERTROPHIC_CARDIOMYOPATHY “, and genes derived from a manually literature search was generated (Supplemental Table 1) and used to identify HCM related genes in the murine transcriptome and proteome.

For Hif-1α target retrieval, the following databases were used: TRANSFAC^77,78^, TTRUST^79^, MSigDB^76^. In additional the TF2DNA Meta database^80^, which include own computational data and data from different sources of experimental and computational data. For TF2DNA^80^ the following sources were used with a connectivity pvalue of 0.0001: Berger 2006 (Experimental), Chen 2011 (Experimental), Gerstein 2012 (Experimental), Jolma 2013 (Experimental), Kulakovskiy 2013 (Experimental), Mathelier 2014 (Experimental), Matys 2006 (Experimental), Scharer 2009 (Experimental), TF2DNA (Computational), Weihrauch 2014 (Experimental).

### HIF target list assembly

To identify genes directly regulated by the Hif-1α transcription factor, a combined dataset was generated from different available public sources for described targets for human Hif-1α. The list was generated from the TRANSFAC database^77,78^, the TTRUST database^79^, the “SEMENZA_HIF1_TARGETS” gene set from the MSigDB^76,81^ and the meta database TF2DNA^80^. After removing duplicates, the list (Supplemental Table 2) was converted into murine orthologues (Supplemental Table 3) and used for further analysis.

### Venn analysis

Venn analysis was performed using the public available Venn analysis tool developed by the Center for Plant Systems Biology, University of Gent, Belgium (https://bioinformatics.psb.ugent.be/webtools/Venn/).

### Quantification of myocardial fibrosis

Hearts were quickly excised from isoflurane-euthanized mice, washed in PBS, fixed overnight in 4% paraformaldehyde, and embedded in paraffin as described previously^19^. Embedded hearts were serially sectioned every 5 μm from apex to base in a transverse plane. Adjacent sections were stained with Masson’s trichrome (Sigma-Aldrich) to assess collagen deposition resulting from fibrosis. Experienced observer in a subset of 5 α-MHC^719/+^, 6 WT, 4 WT/cHif1aKO and 6 α-MHC^719/+^/cHif1aKO independently scored fibrosis and the quantification of myocardial fibrosis was conducted in a blinded fashion.

### Mouse proteomics analysis

Tissue was first pulverized (Pulverizer CP02, Covaris) and then lysed using sonication (Sonopuls HD3200, Bandelin) in 8 M Urea/ 0.5 M ammonium bicarbonate. Samples were first digested with Lys-C (1:100; enzyme:protein) for 4 h at 37° C, then diluted to a concentration of 1 M Urea/50 mM ammonium bicarbonate and after addition of trypsin (1:50; enzyme:protein), digested overnight at 37° C. Samples were analyzed on a Ultimate 3000 RSLC chromatograph coupled to a Q Exactive HF-X mass spectrometer (both Thermo Fisher Scientific). Per sample, 1.5 µg peptides were loaded onto a trap column (PepMap C18; 2 cm; 100 µm; Thermo Fisher Scientific) and separated (PepMap C18 analytical column; 75 µm; 50 cm; Thermo Fisher Scientific) using a two-step gradient: First, a ramp from 3% B to 25% B for 160 min, followed by a 10 min ramp to 40% B (A: 0.1% formic acid in water; B: 0.1% formic acid in acetonitrile). The mass spectrometer was run in data dependent mode and per survey scan a maximum of 15 product spectra were acquired. Raw data was then searched with MaxQuant (1.6.7.0)^82^ against all Swiss-Prot mouse entries and the built-in contaminant database. Match between runs and label free quantification were turned on. The mass spectrometry proteomics data have been deposited to the ProteomeXchange Consortium (http://proteomecentral.proteomexchange.org) via the PRIDE partner repository^83^ with the dataset identifier PXD038911. All statistical analyses and data visualization were performed using R Statistical Software (R Core Team 2021). Proteins having at least two peptides detected in at least two replicates of each condition were tested for differential abundance using the MS-EmpiRe algorithm^84^ as described in^85^. Proteins with a Benjamini–Hochberg (BH)-corrected p-value ≤ 0.05 and fold-change ≥ 1.5 were regarded as significant. The STRING pre-ranked gene set enrichment analysis^86^ was used to reveal biological processes associated with differentially abundant proteins. Signed (based on fold-change) and log-transformed p-values were used as ranking metrics and false discovery rate was set to 0.05. To minimize redundancy of the significantly enriched biological processes, the REVIGO tool^87^ was used.

### Human serum sample preparation

The authors assert that all procedures contributing to this work comply with the ethical standards of the relevant national guidelines on human experimentation and with the Helsinki Declaration of 1975, as revised in 2008, and have been approved by the Technical University of Munich Ethics Committee (Project number 332/15, approved on July 29^th^ 2015). Patient information and consent to the storage of anonymized data, scientific evaluation and anonymized publication was obtained in writing from all patients after written and oral information. Informed consent was also obtained from all patients and/or their legal guardians. Peripheral blood was collected during routine clinical phlebotomy.

In total, blood samples from 16 patients with early onset of HCM and age- and gender-matched patients without structural heart disease and heart failure were included in the study. Blood was collected in 9 ml serum collection tubes (S-Monovette, Sarstedt AG & Co) each, allowed to clot for 30 min and subsequently centrifuged at 3,400 × g for 10 min at room temperature (RT). Within 10 min of separation, resulting serum was aliquoted and stored at -80 °C.

### Human serum proteomics analysis

To overcome the high dynamic range of blood protein and achieve deeper coverage of serum proteins the samples were depleted using Thermo Scientific High Select Top14 Abundant Protein Depletion Mini Spin Columns. The resulting proteins were subsequently digested by trypsin during filter-aided sample preparation (FASP) onto Microcon 30 kDa cut-off filters (Merck Millipore). Samples were analyzed using RSLCnano system combined with an Orbitrap Q Exactive HF-X with Digital PicoView 550 nanospray ion source. The data were acquired using BoxCar acquisition method over 90 min LC gradient. Spectral library of serum proteins for subsequent bioinformatic analysis was acquired using standard data-dependent acquisition over the same 90 min LC gradient. The library was created using twice depleted pooled serum sample fractioned into 50 fractions.

The raw data were analyzed using MaxQuant software (v. 1.6.14.0) and MS/MS data were searched against the UniProtKB Human FASTA database and cRAP contaminant database. Match between run algorithms was used across the whole dataset to improve the identification rate among BoxCar data using spectral library.

The MaxQuant data were subsequently analyzed using KNIME analytical platform using publicly available tools developed for proteomics analysis on GitHub (https://github.com/OmicsWorkflows). All potential contaminants, PGs with measured intensity in less than 6 samples of either group, and PGs without any identification on two and more peptide in one or the other group were filtered out. The filtered dataset log2 transformed intensity values were normalized using loess normalization and missing values were imputed using imp4p analysis R package. Imputed data were used for the comparative analysis of the study groups using limma test.

### Statistics

Number of animals used was based on previous published results^8,55^. As the specific research question was to investigate whether a cardiomyocyte-specific Hif-1α knockout has a beneficial effect on the pathology of the development of the HCM phenotype observed in the α-MHC^719^^/+^ mice, sample size was confirmed by sample size calculation. For this the power.t.test of R (R version 4.4.0) was used with a power of 0.8, significance level of 0.05, assumed delta of 0.5 and standard deviation of 0.2. These settings were based on previous experiments^8^ under the assumption that only two groups (α-MHC^719/+^/cHif1aKO vs. α-MHC^719/+^) were compared. Prior to plotting and statistical analysis, an outlier calculation using the “Grubb´s test” to identify outliers for each experimental group was performed.

The statistical analyses were carried out by SPSS software (IBM, Armonk, New York, U.S.A, and Version 29.0.1.0). First, specified values were checked for normal distribution and variance of variance (F-test from Levene). Accordingly, the data were expressed in mean and standard deviation or median and quartile. For analysis of western blot, immunostaining, Mant-ATP assay, superoxide generation and histology, means were tested with the T-test (parametric data) and medians with the Mann–Whitney test (Wilcoxon test, non-parametric data). For evaluation of the echocardiography the non-parametric Kruskall-Wallis test was used. The dependence between two variables was tested by chi-square. For parametric data a correlation according to Pearson was used, for ordinal or not normally distributed data the correlation according to Spearman or in case of four-field plaque the Fisher’s Exact Test. Graphs were created using GraphPad Prism Version 6 with combined bar and scatter plot except for quantitative PCR data. Quantitative PCR was calculated using the Relative Expression Software Tool^88,89^ based on the ∆∆CT calculation and using a Pair Wise Fixed Reallocation Randomisation Test^89^. Due to this calculation method no individual fc values are possible. A p-value < 0.05 was considered statistically significant. For all statistics a post-hoc power-analysis was performed to confirm a sufficient statistical power.

## Conclusion

In summary, the present study provides evidence for Hif-1α as a major contributor to HCM pathology via oxidative stress, inflammation, and angiogenesis. Inhibition of Hif-1α signaling ameliorates pathological myocardial remodeling in an established HCM mouse model. Targeting Hif-1α signaling may serve as new therapeutic option to mitigate sarcomeric HCM.

## Supplementary Information

Supplementary Information.

## Data Availability

The human mass spectrometry proteomics data have been deposited to the ProteomeXchange Consortium (http://proteomecentral.proteomexchange.org) via the PRIDE partner repository^82^ with the dataset identifier PXD038911. The murine RNA sequencing data will be available at the Geo Omnibus repository under the accession number GSE266649. All other datasets generated during the study are available from the corresponding author on reasonable request.

## Abbreviations

*8-OHG*: 8-Hydroxyguanosine
*Actg1*: Gamma actin
AKT: AKT serine/threonine kinase
α-MHC: Alpha myosin heavy Chain
ATP: Adenosine tri phosphate
Bp: Basepair
BDM: 2,3-Butanedione 2-monoxime
BH: Benjamini–Hochberg
BNIP1: BCL2 interacting protein 1
BW: Body weight
cHif1ako: Cardiomyocyte-specific Hif-1a knockout
Ca9: Carbo anhydrase 9
cDNA: Complementary deoxyribonucleic acid
CMA: 1-Hydroxy-methoxycarbonyl-2,2,5,5-tetramethyl-pyrrolidine hydrochloride
CSA: Cyclosporin
DCM: Dilated cardiomyopathy
DES: Desferroxamine
DETC: Diethyldithiocarbamate
DRX: Disordered relaxed state
DMEM: Dulbeccos modified eagle medium
DNA: Deoxyribonucleic acid
DTT: Dithiothreitol
EDTA: Ethylenediaminetetraacetic acid
EPR: Electron paramagnetic resonance
ES: Enrichment Score
FCS: Fetal calf serum
FDA: Food and drug administration
FDR: False discovery rate
FFPE: Formalin fixed paraffin embedded
FPKM: Fragments per kilobase of transcript per million fragments mapped
G: Gauge
HCM: Hypertrophic cardiomyopathy
HEPES: 4-(2-Hydroxyethyl)-1-piperazineethanesulfonic acid
HIF1A: Hypoxia inducible factor 1A (gene)
Hif-1α: Hypoxia inducible factor 1α (protein)
Hif-2α: Hypoxia inducible factor 2a
HO-1: Heme oxygenase 1
HW: Heart weight
IGF2: Insulin like growth factor 2
KO: Knock out
Lmna: Prelamin A/C
LTBP1: Latent-transforming growth factor beta-binding protein 1
LV: Left ventricle
LVEDD: LV end-diastolic diameter
LVESD: LV end-systolic diameter
MI: Myocardial infarction
MOPS: 3-(*N*-Morpholino)propanesulfonic acid
Myh6: Myosin heavy chain 6
Myh7: Myosin heavy chain 7
P53: Tumor protein p53
PBS: Phosphate buffered saline
PCR: Polymerase chain reaction
PHD2: Prolyl hydroxylase 2
PFKFB: 6-Phosphofructo-2-kinase/fructose-2,6-biphosphatase 2
RECK: Reversion-inducing-cysteine-rich protein with kazal motifs
RIN: RNA integrity number
RNA: Ribonucleic acid
ROS: Reactive oxygen species
RT: Room temperature
RT-qPCR: Reverse transcription quantitative polymerase chain reaction
RV: Right ventricle
SNP: Single nucleotide polymorphism
SRX: Super relaxed state
Tpm4: Tropomyosin alpha-4 chain
WT: Wildtype
