# Supplementary material for: Contribution of hypoxia-inducible factor 1alpha to pathogenesis of sarcomeric hypertrophic cardiomyopathy
Source: Sci Rep. 2025 Jan 16;15:2132. doi: 10.1038/s41598-025-85187-9 (PMC11739497; doi:10.1038/s41598-025-85187-9)
Supplement: Supplementary file 1 — Supplementary Information. [file 41598_2025_85187_MOESM1_ESM.docx]

# SUPPLEMENTARY INFORMATION

**Title: Contribution of hypoxia-inducible factor 1alpha to pathogenesis of sarcomeric hypertrophic cardiomyopathy**

# Supplemental Materials and Methods

**Cell Culture**

Human microvascular endothelial cells (HMEC1; ATCC CRL-3243) were grown in MCDB131 medium (PAN-Biotech, Aidenbach, Germany) supplemented with 2mM L-glutamine (PAN-Biotech), 10 ng/mL EGF (Merck Millipore, Darmstadt, Germany), 1 g/mL hydrocortisone, 10% fetal calf serum (PAN-Biotech), and 100 U/mL penicillin/100 lg/mL streptomycin (PAN Biotech) and maintained at 37°C under an atmosphere of 5% CO_2_. For hypoxic experiments cells were incubated for 4 hours at 1% oxygen in a BakerRuskinn InVivO2 hypoxic work station (I&L Biosystems, Königswinter, Germany).

**Transfection of siRNA**

Transfections with small interfering RNA (siRNA) against Hif1α were performed by using Lipofectamine 3000 (Life Technologies GmbH, Darmstadt, Germany) following the manufacture´s instruction. The following following siRNAs were used: against human HIF1A with the sequence: 5´-UCA AGU UGC UGG UCA UCA GdTdT-3´ targeting position 1543–1561 bp of human HIF1A mRNA - NCBI accession No. NM_001530 (Eurogentec, Köln,Germany) and the AllStars Negative control siRNA (Qiagen) as negative control.

# Supplemental Figures

## Supplemental Figure 1: Study Design


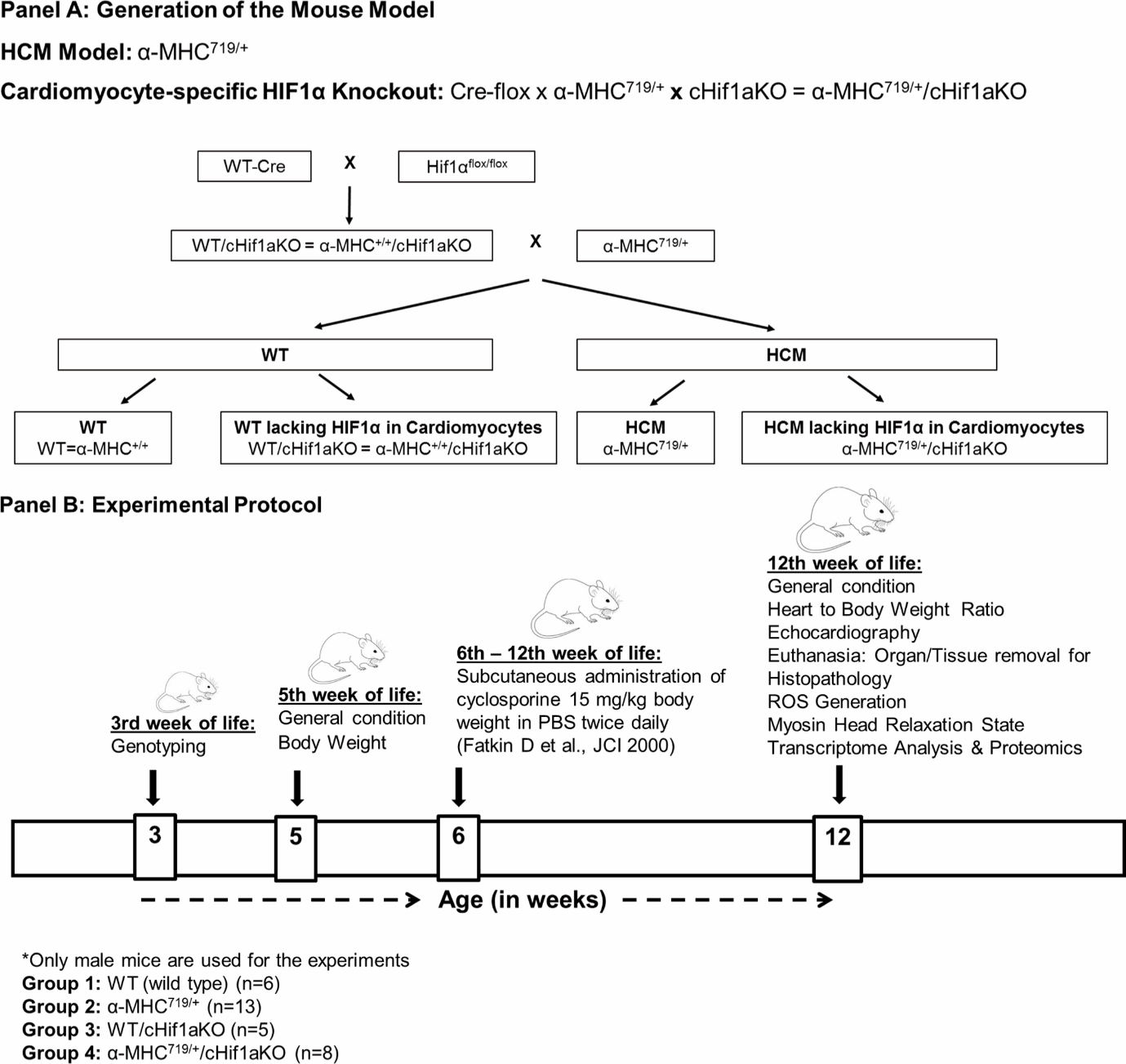


## Supplemental Figure 2: 30 weeks old α-MHC^719/+^ mice showed increased Hif-1α protein levels compared to age-matched wildtype mice. Original western blot are presented in Supplemental Figure 11

**30w WT**

**PoncS**


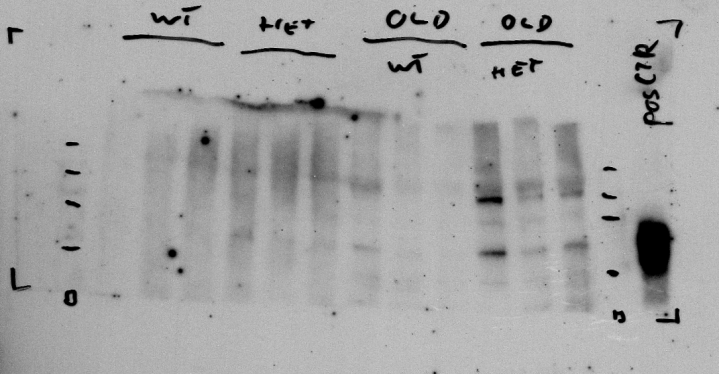

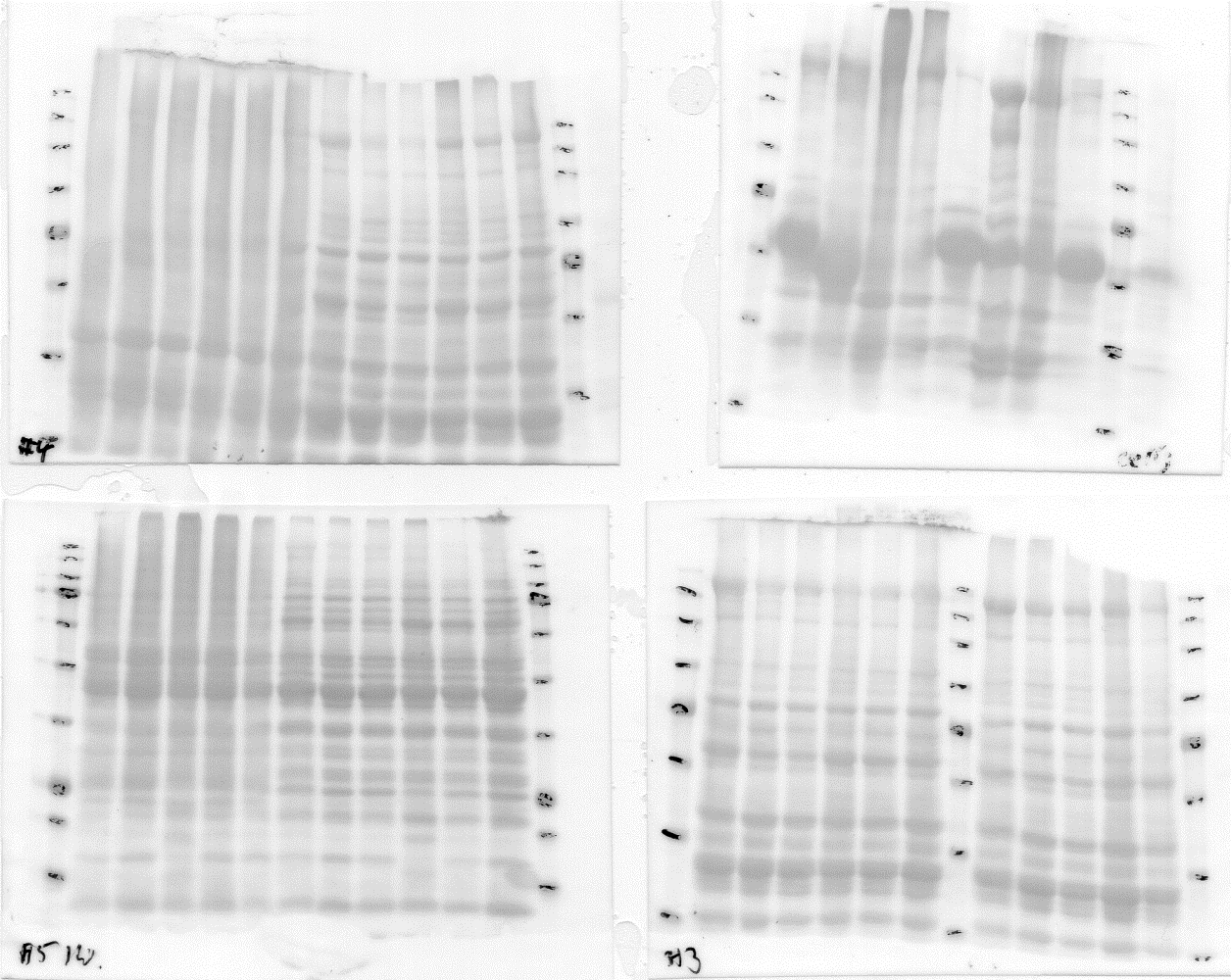


**Hif-1α**

**30w α-MHC719/+**

## Supplemental Figure 3: α-MHC^719/+^ mice showed reduced αMHC expression with a

## tendency of recovery in α-MHC^719/+^ mice lacking Hif1a in cardiomyocytes


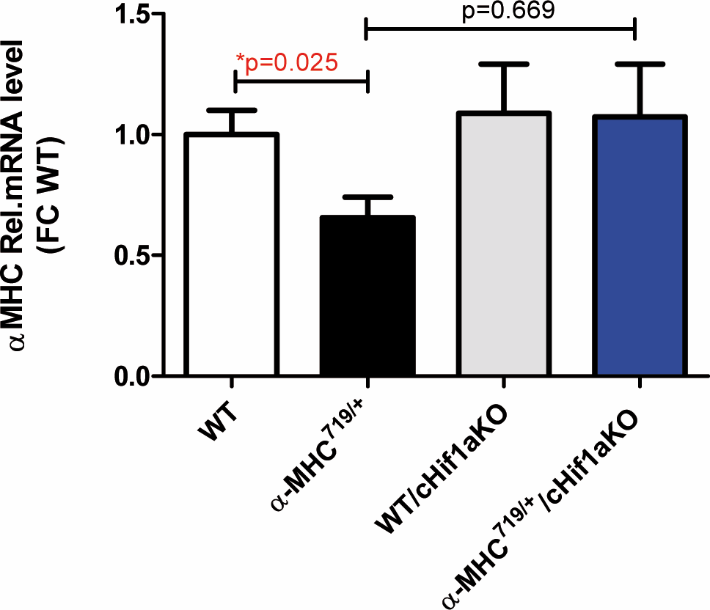


## Supplemental Figure 4: Transcriptome Heat Map


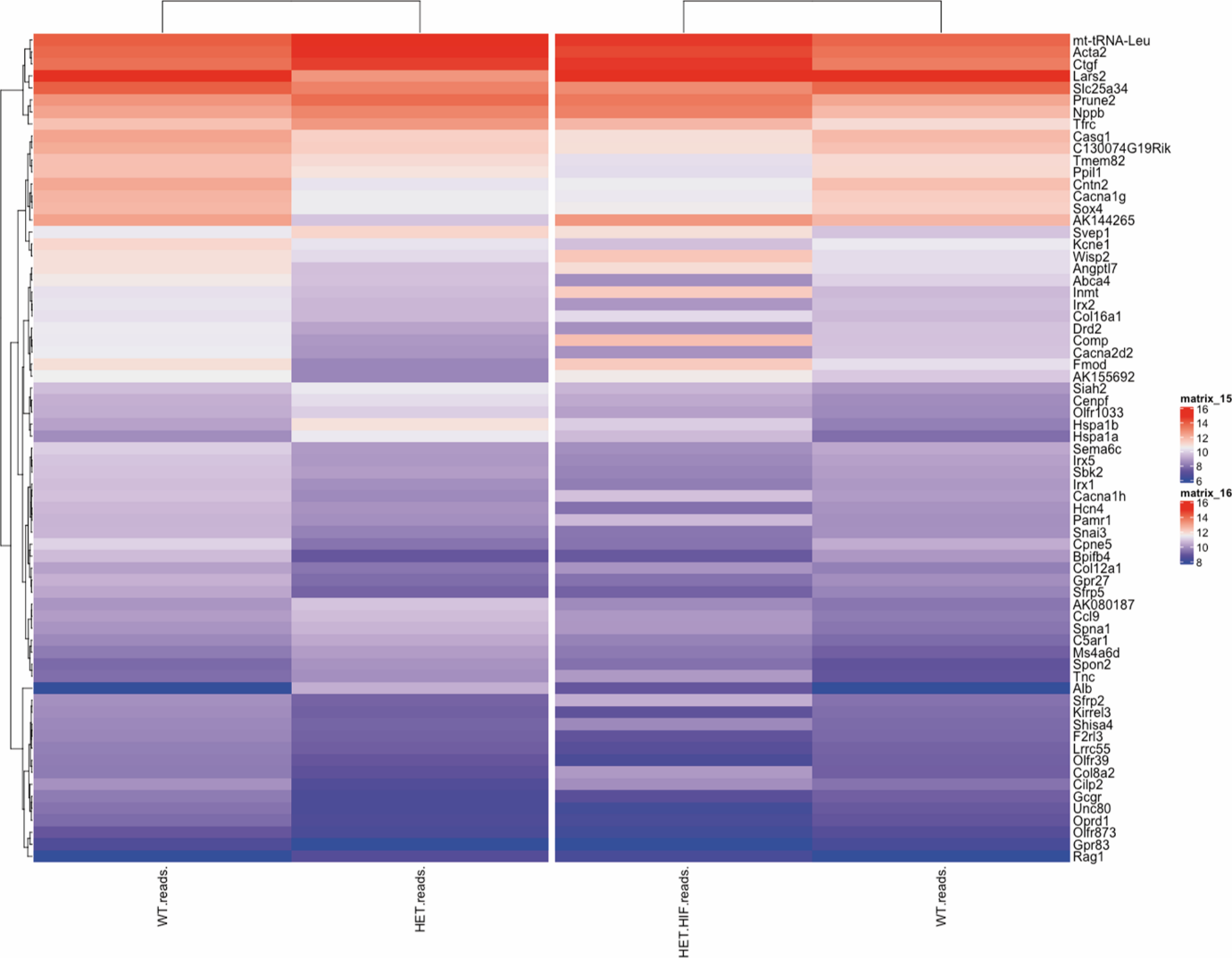


## Supplemental Figure 5: SOD3 is upregulated in left ventricular tissue of α-MHC^719/+^ mice. Original western blots are presented in Supplemental Figure 12


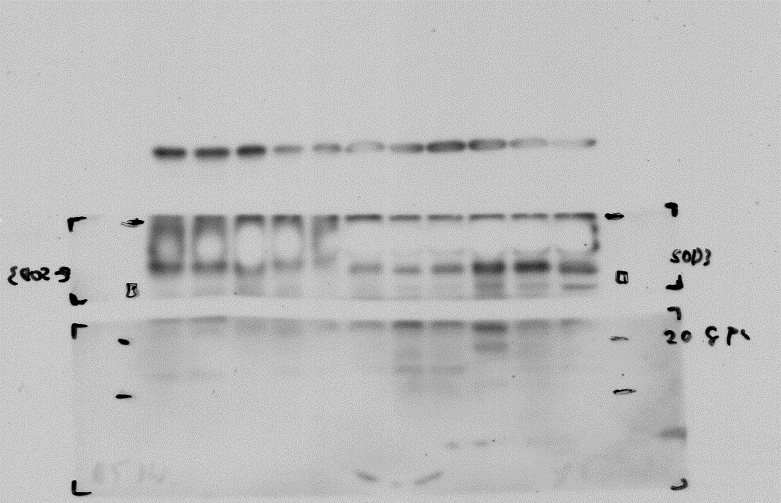

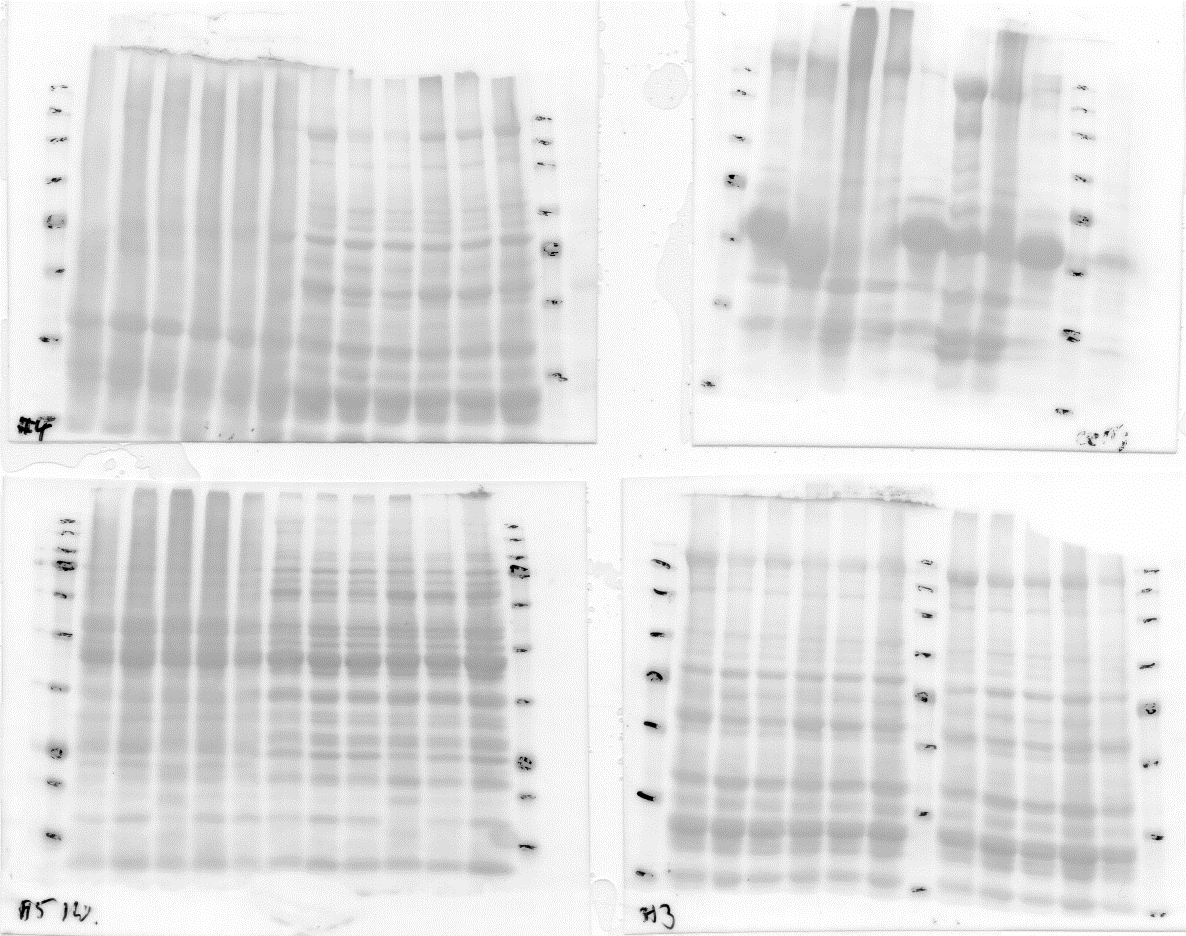


**WT**

**PoncS**

**SOD3**

**α-MHC^719/+^**

## Supplemental Figure 6: Echocardiographic parameters of 12 weeks old WT, α-MHC^719/+^, WT/cHif1 αKO and α-MHC^719/+^/cHif1 αKO. Compared to WT, LVEDD (panel A), and LVESD (panel B) were lesser in α-MHC^719/+^ mice. However, there was no appreciable difference in the Heart rate (panel C) and Ejection Fraction (panel D) between the WT and α-MHC^719/+^ mice

**A**

**D**

**C**

**B**

## Supplemental Figure 7: Human microvascular endothelial cells were transfected with either a control siRNA (siCtr) or siRNA against HIF-1α followed by 4 hours hypoxia at 1% oxygen. Western blot was performed for Hif-1α. Ponceau S staining served as loading control


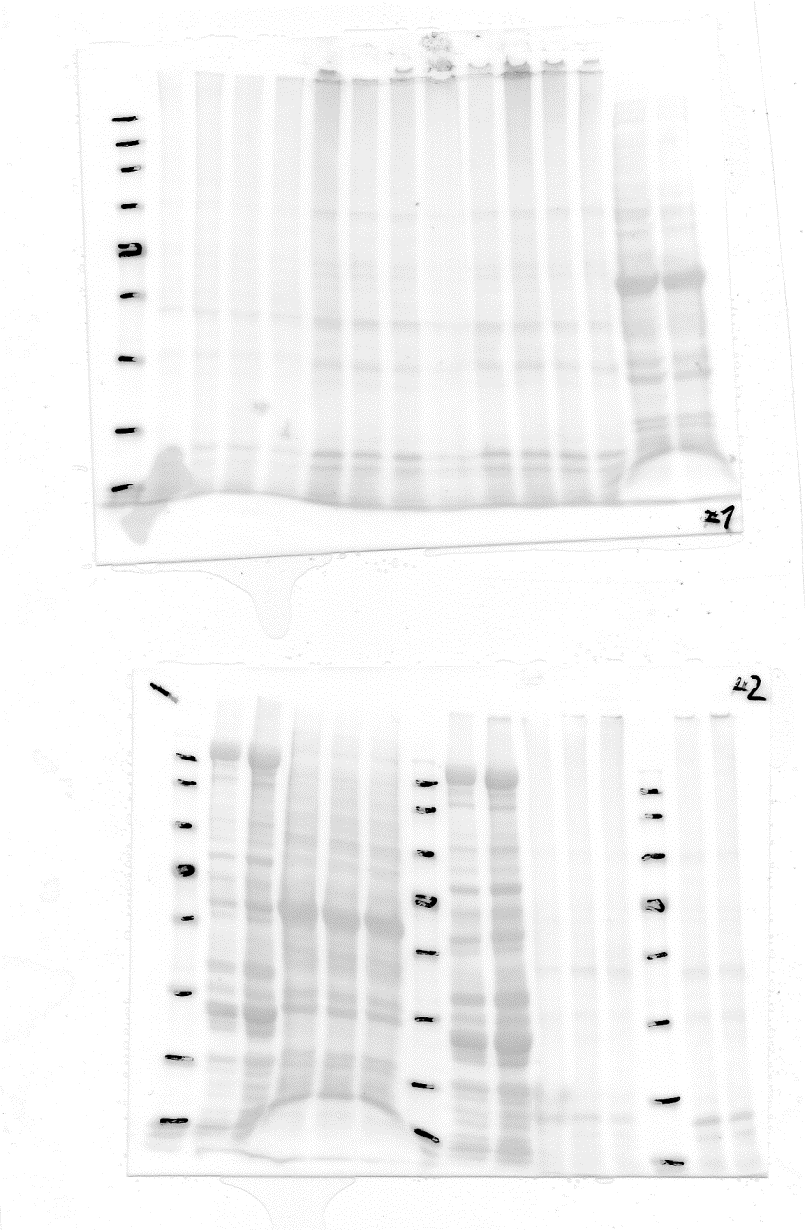

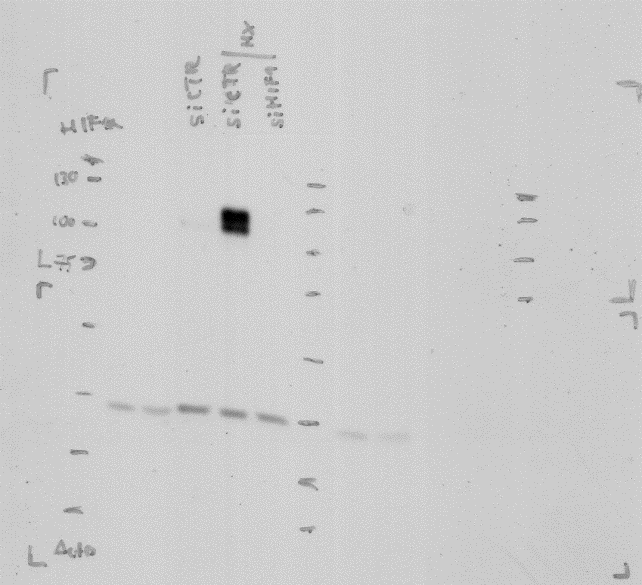


**PoncS**

**Hif-1α**

**Hx**

**siHif1A**

**siCtr**

**siCtr**

## Supplemental Figure 8: Original Western blots used as representative images for Figure 2A. Boxes indicate area of interest. Additional crossed out bands indicate other proteins detected in the same western blot experiment irrelevant for the Figure


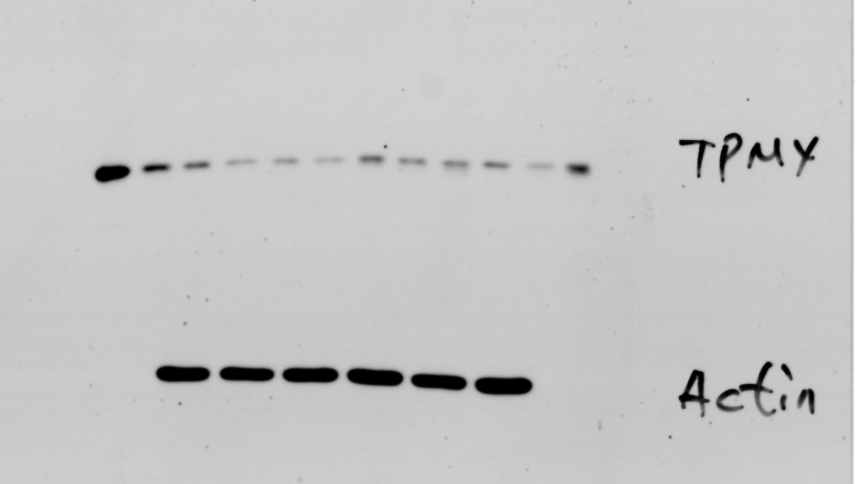

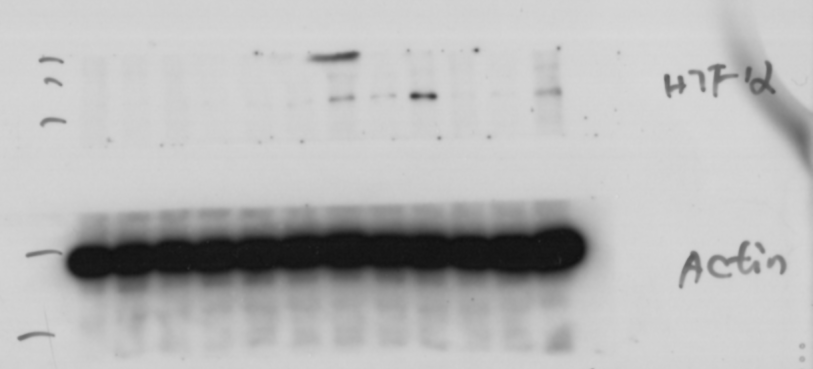


**Hif-1α**

**Tpm4**


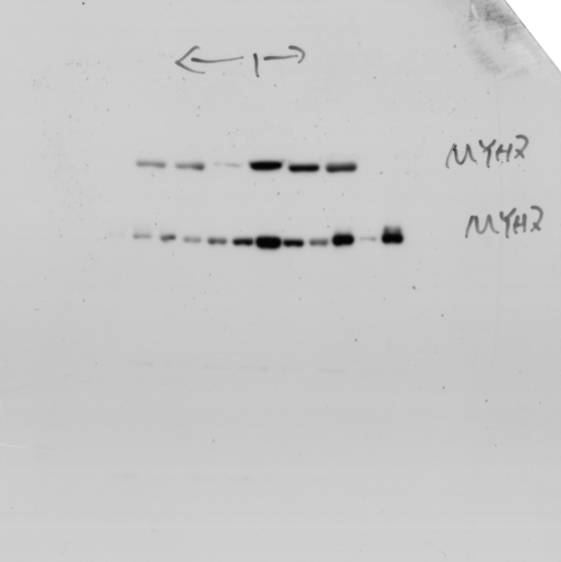


**Myh7**


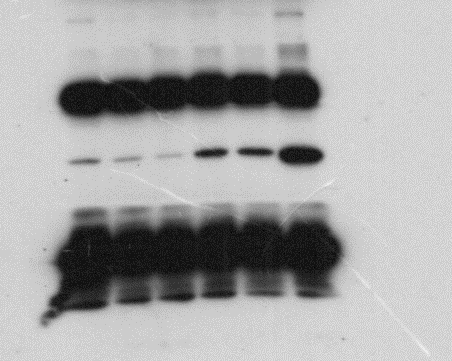


**Des**


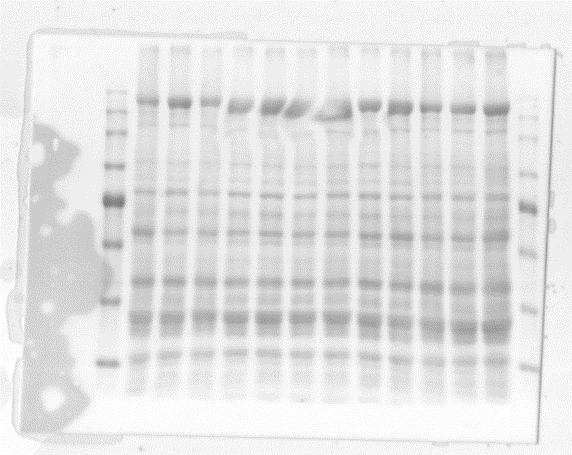


**Ponceau S**

**100kd**

**135kd**

**180kd**

**180kd**

**245kd**

**63kd**

**45kd**

**35kd**

**25kd**

## Supplemental Figure 9: Original Western blots used as representative images for Figure 3B. Boxes indicate area of interest. Additional crossed out bands indicate other proteins detected in the same western blot experiment irrelevant for the Figure


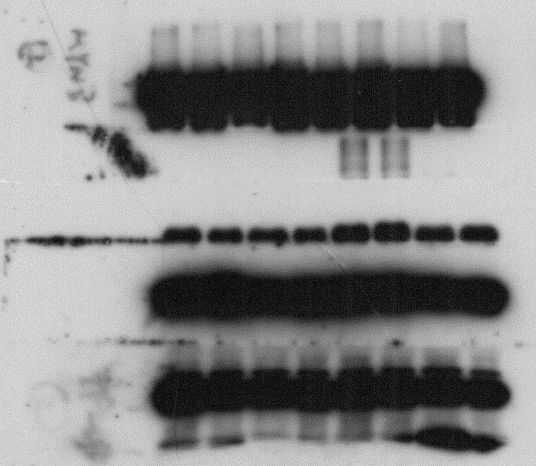

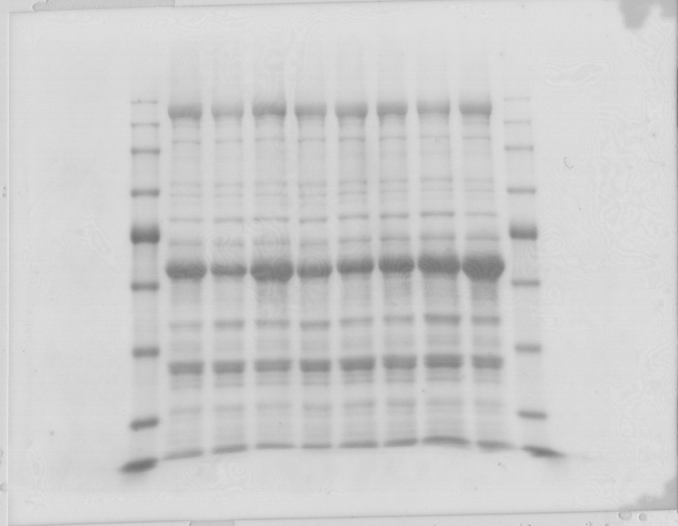


**Hif-1α**

**Ponceau S**

**100kd**

**135kd**

## Supplemental Figure 10: Original Western blots used as representative images for Figure 3E. Boxes indicate area of interest. Additional crossed out bands indicate other proteins detected in the same western blot experiment irrelevant for the Figure


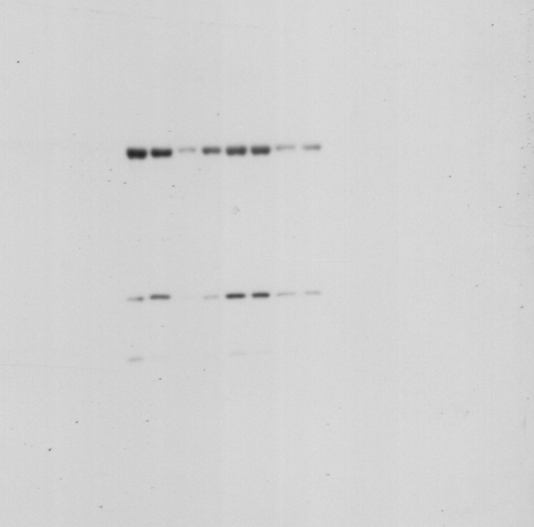

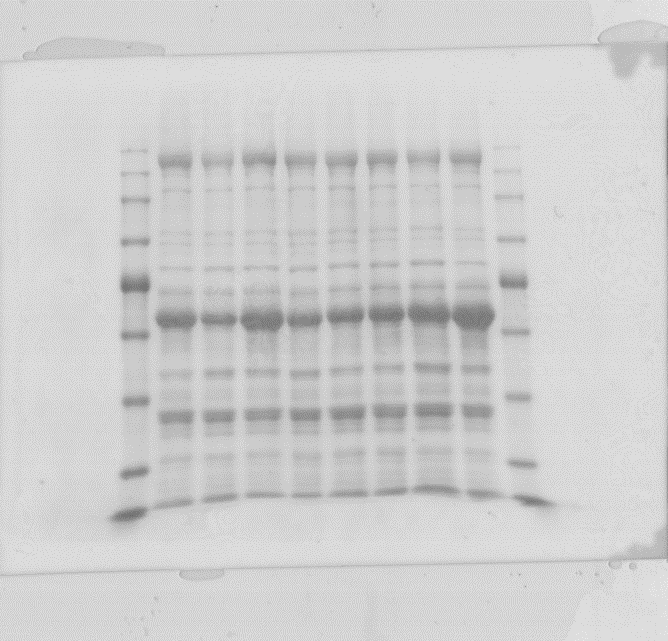

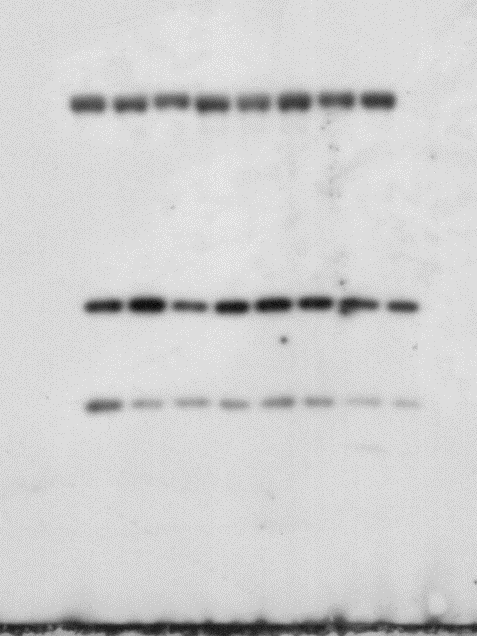


**Tpm4**

**Des**

**Ponceau S**

**180kd**

**245kd**

**63kd**

**45kd**

**35kd**

**25kd**

**Myh7**

## Supplemental Figure 11: Original Western blots used of Hif-1α used for Supplemental Figure 2. Blue boxes indicate area of interest. Additional crossed out bands indicate other proteins detected in the same western blot experiment irrelevant for the Figure


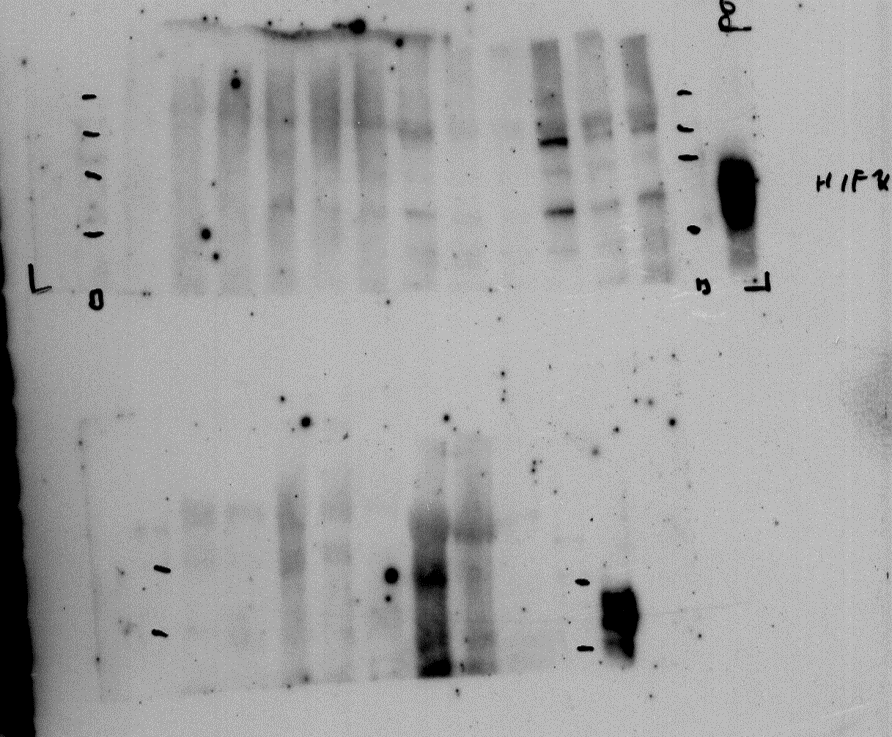

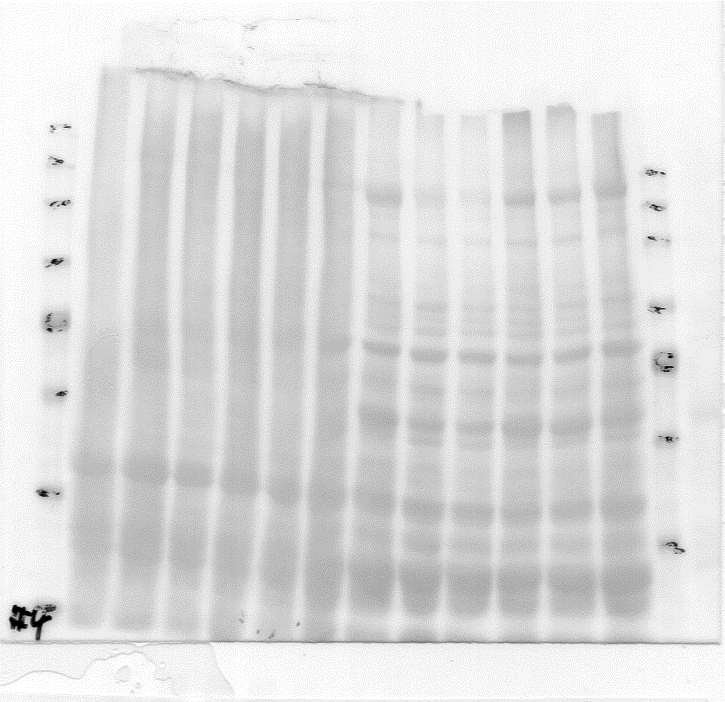


**Ponceau S**

**Hif1a**

**100kd**

**135kd**

**180kd**

**245kd**

## Supplemental Figure 12: Original Western blots used of SOD3 used for Supplemental Figure 5. Blue boxes indicate area of interest. Additional crossed out bands indicate other proteins detected in the same western blot experiment irrelevant for the Figure


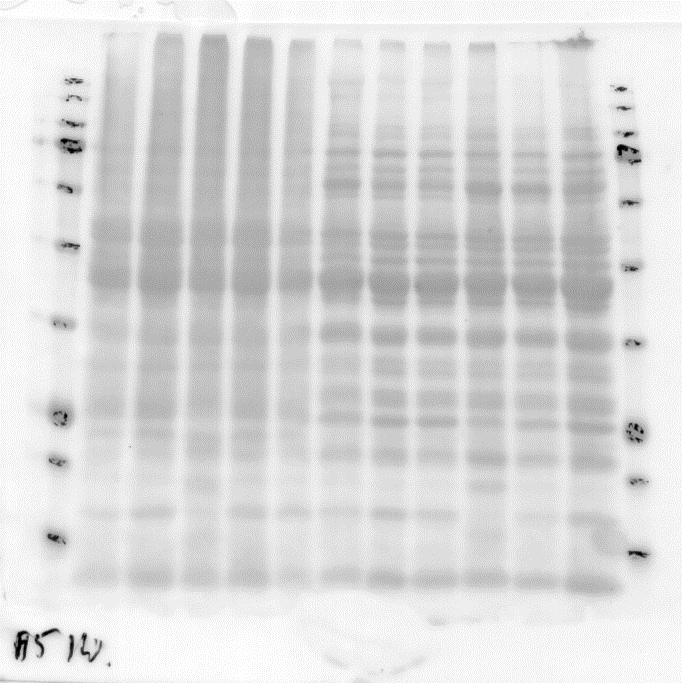

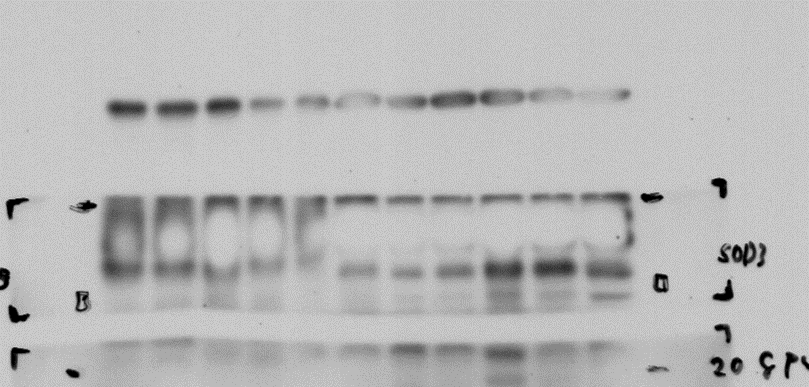


**Ponceau S**

**SOD3**

**35kd**

**25kd**

## Supplemental Figure 13: Representative example of cutting the WB membrane to detect Myh7, Hif-1α, Desmin (Des) and Tpm4


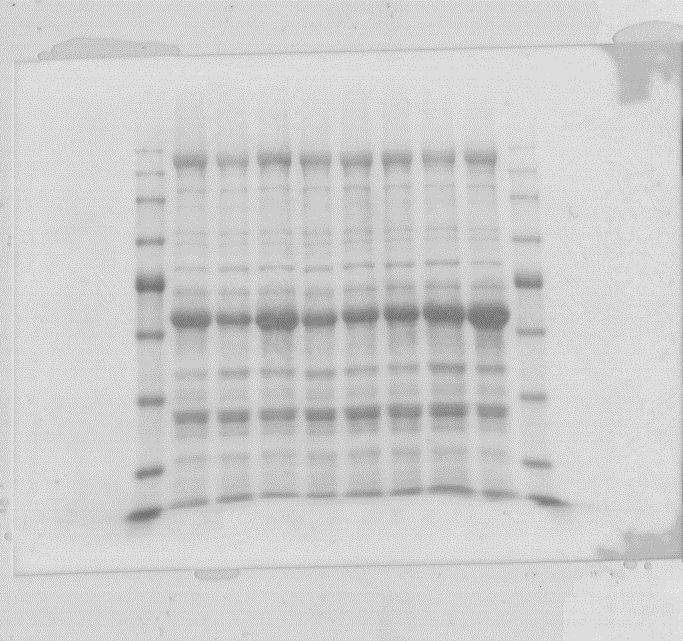


**Ponceau S**

75kd

100kd

135kd

180kd

235kd

63kd

45kd

35kd

**Tpm4**

**Des**

**Myh7**

**Hif1a**

25kd

# Supplemental Tables

## Supplemental Table 1: Echocardiographic parameters of 12 weeks old WT, α-MHC^719/+^, WT/cHif1 αKO and α-MHC^719/+^/cHif1 αKO bpm, all treated with cyclosporine; bpm: beats per minute; p-value: Kruskal-Wallis-H for multiple variables; data are expressed as mean ±standard deviation [median; min-max]

| **Echocardiographic parameter** | **WT**  **N=6** | **α-MHC^719/+^**  **N=13** | **WT/cHif1 αKO**  **N=5** | **α-MHC^719/+^/cHif1 αKO**  **N=8** | **Kruskal-Wallis**  **p-value** | **WT**  vs  **α-MHC^719/+^**  **p-value** | **α-MHC^719/+^**  vs  **α-MHC^719/+^/cHif1 αKO**  **p-value** |
| --- | --- | --- | --- | --- | --- | --- | --- |
| **Heart Rate (bpm)** | 607±84  [627;450-676] | 586±80  [552;496-750] | 633±18  [633;615-662] | 599±106  [613;359-730] | 0.424 | 1.000 | 1.000 |
| **Maximal left ventricular end-diastolic myocardial thickness (mm)** | 0.71±0.07  [0.7;0.6-0.8] | 1.07±0.23  [1.1;0.6-1.5] | 0.63±0.06  [0.7;0.6-0.7] | 0.67±1.12  [0.7;0.5-0.8] | <0.001 | <0.001 | <0.001 |
| **Left ventricular end-diastolic diameter (mm)** | 2.6±0.4  [2.6;2.3-3.1] | 2.5±0.6  [2.3;1.9-3.7] | 3.2±0.3  [3.2;2.8-3.7] | 3.1±0.5  [3.1;2.3-4.0] | 0.019 | 1.000 | 0.148 |
| **Left ventricular end-systolic diameter (mm)** | 1.4±0.3  [1.4;1.0-1.8] | 1.1±0.4  [1.1;0.6-2.1] | 2.1±0.5  [2.1;1.5-2.7] | 1.7±0.6  [1.6;1.0-3.1] | 0.005 | 1.000 | 0.056 |
| **Ejection fraction (%)** | 65±17  [64;42-87] | 77±19  [86;43-96] | 44±7  [43;36-53] | 52±8  [52;42-68] | 0.003 | 0.581 | 0.004 |

## Supplemental Table 2: HCM Geneset

| **Murine HCM geneset derived from the Molecular Signatures Database v7.5.1**[**^1^**](#_ENREF_1) **(datasets M35367 and M8728) and manual literature search** | | | | | | |
| --- | --- | --- | --- | --- | --- | --- |
| Abcc9 | Il1a | Slc33a1 | Itga6 | Cox7b | Mlxipl | Pet100 |
| Ace | Il1b | Sln | Itga7 | Cox14 | Mrap | Pmm2 |
| Ace3 | Il33 | Slpi | Itga8 | Cox15 | Mras | Ppa2 |
| Acta1 | Il6 | Smad2 | Itga9 | Cpt1a | Mrpl3 | Ppp1cb |
| Acta2 | Jag2 | Spp1 | Itga10 | Cryab | Mrpl44 | Ppp1r21 |
| Actb | Jak1 | Stat1 | Itga11 | Dld | Mrps14 | Ptpn11 |
| Actg2 | Jkamp | Syngap1 | Itgav | Dlk1 | Mrps22 | Pygl |
| Adam8 | Jkampl | Tgfb1 | Itgb1 | Dnajc30 | mt-Atp6 | Pygm |
| Ahsg | Ldlrad3 | Tgfb2 | Itgb3 | Echs1 | mt-Co1 | Qrsl1 |
| Akt1 | Lif | Tgfb3 | Itgb4 | Eif4h | mt-Co3 | Rad51 |
| Akt1s1 | Ltbp2 | Thbs1 | Itgb5 | Elac2 | mt-Nd1 | Rad51c |
| Akt2 | Ltbp3 | Thbs4 | Itgb6 | Ercc4 | mt-Nd2 | Raf1 |
| Akt3 | Map2k3 | Timp1 | Itgb7 | Fah | mt-Nd3 | Rfc2 |
| Alyref | Map2k7 | Tnc | Itgb8 | Fanca | mt-Nd4 | Rfwd3 |
| Alyref2 | Mapk1 | Tnf | Lama2 | Fancb | mt-Nd5 | Rit1 |
| Arg2 | Mapk3 | Tnfaip1 | Lmna | Fancc | mt-Nd6 | Rnaseh2a |
| Atp2a2 | Mapk8 | Tnfaip2 | Mybpc3 | Fancd2 | mt-Te | Rnaseh2b |
| B4galnt1 | Med23 | Tnfaip3 | Myl2 | Fance | mt-Tf | Rnaseh2c |
| Bicdl1 | Mef2a | Tnfaip6 | Myl3 | Fancf | mt-Th | Rtl1 |
| Birc5 | Mfap2 | Tnfaip8 | Prkab2 | Fancg | mt-Tk | Samhd1 |
| Calr | Mmp2 | Tnfaip8l1 | Prkag1 | Fanci | mt-Tl1 | Sardh |
| Camk2a | Mmp9 | Tnfrsf11b | Prkag2 | Fancl | mt-Tn | Sco2 |
| Ccn2 | mt-Co2 | Tnfrsf14 | Prkag3 | Fancm | mt-Tq | Sdha |
| Cdk1 | Myc | Tnnc1 | Ryr2 | Fastkd2 | mt-Ts1 | Sdhaf1 |
| Cdk2 | Myef2 | Tnni1 | Sgca | Fbxl4 | mt-Ts2 | Sdhb |
| Cic | Myef2l | Tnni2 | Sgcb | Fhl1 | mt-Tv | Sdhd |
| Cilp | Myh6 | Tnni3 | Sgcd | Fhod3 | mt-Tw | Sgsh |
| Clcn7 | Myh7 | Tnni3k | Sgcg | Fkbp6 | Mtfmt | Shmt2 |
| Col11a1 | Myl7 | Tnnt2 | Slc8a1 | Flnc | Mto1 | Shoc2 |
| Col16a1 | Nanos3 | Tnnt3 | Tpm1 | Fos | Mylk2 | Slc2a10 |
| Col1a1 | Nfat5 | Top2a | Tpm2 | Foxred1 | Myoz2 | Slc19a3 |
| Col1a2 | Nfatc4 | Trdn | Tpm3 | Fto | Mypn | Slc22a5 |
| Col3a1 | Nfkb1 | Trim59 | Tpm4 | Fxn | Naga | Slc25a3 |
| Col5a2 | Nfkb2 | Ttc9 | Ttn | Gaa | Naglu | Slc25a4 |
| Col8a1 | Nfkbie | Usf2 | Aars2 | Gla | Ncf1 | Slc30a10 |
| Crlf1 | Nmt1 | Vcam1 | Abcc8 | Glb1 | Ndufa1 | Slx4 |
| Ctf1 | Nos1 | Vegfa | Abhd11 | Gnptab | Ndufa2 | Smc1a |
| Cxxc1 | Nos3 | Vegfb | Acad9 | Gns | Ndufa4 | Sos1 |
| Cyba | Nox4 | Vgll2 | Acadvl | Gpr101 | Ndufa6 | Star |
| Dusp4 | Nppa | Vsig4 | Adar | Gtf2i | Ndufa9 | Stx1a |
| Egf | Nppb | Xirp2 | Agk | Gtf2ird1 | Ndufa10 | Suclg1 |
| Egr3 | Nppc | Zbtb46 | Agpat2 | Gtf2ird2 | Ndufa11 | Sufu |
| Eln | Npr1 | Actc1 | Aip | Gtpbp3 | Ndufa12 | Surf1 |
| F10 | Nr1h2 | Actg1 | Anks6 | Hadh | Ndufa13 | Syne1 |
| Fn1 | Nr1h3 | Cacna1c | Atad3a | Hadha | Ndufaf1 | Syne2 |
| Foxs1 | Nrg1 | Cacna1d | Atp5e | Hccs | Ndufaf2 | Taco1 |
| Fxyd6 | Pdgfa | Cacna1f | Atp5md | Hgsnat | Ndufaf3 | Tafazzin |
| Gata4 | Pik3ca | Cacna1s | Atp6v1a | Hras | Ndufaf4 | Tango2 |
| Gata6 | Pik3cb | Cacna2d1 | Atpaf2 | Hsd17b10 | Ndufaf5 | Tapt1 |
| Gdf15 | Pik3cd | Cacna2d2 | Bag3 | Ifih1 | Ndufaf6 | Tbl2 |
| Gdf6 | Pik3cg | Cacna2d3 | Baz1b | Il12b | Ndufaf8 | Timmdc1 |
| Glipr2 | Pik3r1 | Cacna2d4 | Bcl7b | Insr | Ndufb3 | Tkfc |
| Gm4302 | Pln | Cacnb1 | Bcs1l | Kcnj8 | Ndufb8 | Tmem43 |
| Gm4307 | Postn | Cacnb2 | Bola3 | Kcnj11 | Ndufb9 | Tmem70 |
| Gm4312 | Ppara | Cacnb3 | Braf | Kif20a | Ndufb10 | Tmem126a |
| Gm4340 | Pparg | Cacnb4 | Brca1 | Klf1 | Ndufb11 | Tmem126b |
| Gm8909 | Ppargc1a | Cacng1 | Brca2 | Kras | Ndufs1 | Tmem270 |
| Gsk3b | Ppargc1b | Cacng2 | Brip1 | Lamp2 | Ndufs2 | Tpi1 |
| H2-D1 | Ppp3ca | Cacng3 | Bscl2 | Lat2 | Ndufs3 | Trex1 |
| H2-K1 | Prkaa1 | Cacng4 | Bud23 | Lias | Ndufs4 | Tsfm |
| H2-Q1 | Prkaa2 | Cacng5 | Cav1 | Limk1 | Ndufs6 | Ttpa |
| H2-Q10 | Prkab1 | Cacng6 | Cav3 | Lipt1 | Ndufs7 | Txnrd2 |
| H2-Q2 | Ptgs2 | Cacng7 | Cavin1 | Lrpprc | Ndufs8 | Ube2t |
| H2-Q8 | Rasa1 | Cacng8 | Cldn3 | Lztr1 | Ndufv1 | Ucp2 |
| H2-T3 | Rasa2 | Dag1 | Cldn4 | Mad2l2 | Ndufv2 | Uqcrfs1 |
| Hand2 | S100a4 | Des | Clip2 | Map2k1 | Nek8 | Vcl |
| Hepacam | Sdc1 | Dmd | Cln3 | Map2k2 | Nf1 | Vps33a |
| Hif1a | Sec61b | Emd | Coa5 | Mc2r | Nfs1 | Vps37d |
| Hopx | Serpina3a | Itga1 | Coa6 | Meg3 | Nnt | Xrcc2 |
| Icam1 | Serpina3f | Itga2 | Coa8 | Men1 | Nras | Yars2 |
| Icam5 | Serpina3i | Itga2b | Cog7 | Mettl27 | Nubpl |  |
| Igf1 | Sgpp1 | Itga3 | Coq2 | Micos13 | Opa1 |  |
| Igf2 | Sirt3 | Itga4 | Coq4 | Mipep | Palb2 |  |
| Il17d | Skp2 | Itga5 | Cox6b1 | Mlx | Pdha1 |  |

## Supplemental Table 3 – Human HIF-1α target list

| **Combined human HIF-1α target list derived from TRANSFAC**[**^2^**](#_ENREF_2)**^,^**[**^3^**](#_ENREF_3)**. TTRUST**[**^4^**](#_ENREF_4)**.MsigDB**[**^1^**](#_ENREF_1)**^,^**[**^5^**](#_ENREF_5) **and TF2DNA**[**^6^**](#_ENREF_6) | | | | | | |
| --- | --- | --- | --- | --- | --- | --- |
| AARSD1 | CHMP2A | FTO | LRRC36 | PGGT1B | SEZ6L2 | TRIM71 |
| AATF | CHMP4B | FUOM | LRRC57 | PGK1 | SF1 | TRIP6 |
| ABCA2 | CHRM2 | FXYD5 | LRRK2 | PGM3 | SFT2D3 | TRIT1 |
| ABCB1 | CHRNA3 | FZD7 | LRRTM1 | PHACTR3 | SFXN4 | TRMT6 |
| ABCB6 | CHTF18 | G0S2 | LTBP1 | PHF12 | SGK1 | TRPC4AP |
| ABCC4 | CHURC1-FNTB | G6PC3 | LTBP4 | PHLPP1 | SH3BGR | TRPM5 |
| ABCF1 | CIRH1A | GABRD | LY6H | PHPT1 | SH3YL1 | TRPM7 |
| ABHD17C | CITED2 | GADD45B | LY75 | PHYHD1 | SHCBP1 | TSGA10IP |
| ABI2 | CKLF-CMTM1 | GAGE1 | LYPD5 | PHYHIPL | SHE | TSKU |
| ACE | CLIP3 | GAGE2B | MAB21L3 | PHYKPL | SHISA6 | TSPAN4 |
| ACE2 | CLSTN3 | GAGE2C | MAFF | PIGA | SHROOM4 | TSSK3 |
| ACKR1 | CLUH | GAGE2D | MAGED1 | PIGP | SIK1 | TTC3 |
| ACOXL | CMTR1 | GALK2 | MAMDC4 | PIGW | SIRT1 | TTC39C |
| ACP6 | CNNM4 | GALNT2 | MANBA | PIK3CA | SLC12A4 | TTLL7 |
| ACVR1B | CNPY3 | GAPDH | MAP1LC3B | PIK3IP1 | SLC12A5 | TUBAL3 |
| ACY3 | CNTD2 | GBE1 | MAP3K1 | PIK3R1 | SLC12A7 | TUBB3 |
| ADAL | CNTLN | GCA | MAP3K13 | PIM1 | SLC16A13 | TUBB6 |
| ADAM30 | CNTN2 | GCC2 | MAP3K8 | PITHD1 | SLC16A3 | TUFT1 |
| ADAMTS14 | COIL | GCK | MAP4K1 | PITPNB | SLC22A13 | TWIST1 |
| ADAP1 | COL25A1 | GCM2 | MAP6 | PITPNC1 | SLC22A23 | TWIST2 |
| ADCY3 | COMMD3 | GCSH | MAP7D2 | PITRM1 | SLC25A12 | TWSG1 |
| ADIG | COMMD3-BMI1 | GDA | MAPK15 | PITX2 | SLC25A36 | TXLNA |
| ADK | COMMD4 | GDNF | MAPK4 | PIWIL1 | SLC25A39 | TXLNG |
| ADM | COPZ1 | GFRA2 | MAPK7 | PKM | SLC25A43 | TYROBP |
| ADM2 | COQ9 | GGACT | MAPRE2 | PLA2G6 | SLC25A46 | U2AF2 |
| ADNP | COX4I2 | GHRHR | MARCKS | PLAG1 | SLC26A10 | UBA1 |
| ADO | CP | GID4 | MARCKSL1 | PLAGL1 | SLC29A1 | UBA2 |
| ADPRHL1 | CPEB2 | GK | MATK | PLAU | SLC2A1 | UBA3 |
| ADRA1B | CPLX2 | GLMN | MAX | PLCD3 | SLC2A10 | UBA5 |
| AEN | CPLX3 | GLRX5 | MBP | PLCZ1 | SLC2A3 | UBA6 |
| AGAP1 | CPNE2 | GLS2 | MBTPS2 | PLEKHA6 | SLC35E1 | UBA7 |
| AGO4 | CPNE7 | GMCL1 | MC5R | PLEKHG3 | SLC35E3 | UBE2Z |
| AGPAT1 | CREB3 | GMFB | MCM8 | PLEKHH1 | SLC36A1 | UBR3 |
| AGTR1 | CREB3L1 | GMPPA | ME3 | PLIN5 | SLC36A3 | UBR4 |
| AIF1 | CREM | GMPPB | MEA1 | PMP2 | SLC37A4 | UBXN10 |
| AIFM3 | CRMP1 | GNAI1 | MED20 | PNLDC1 | SLC39A7 | UBXN11 |
| AK3 | CRY2 | GNB2 | MEF2C | PNOC | SLC43A1 | UHRF1 |
| AK4 | CSMD3 | GOLGA4 | MEGF6 | POGLUT1 | SLC43A3 | ULK3 |
| AK9 | CSNK1G1 | GOLT1A | MEIS2 | POLA1 | SLC4A11 | UMODL1 |
| AKAP10 | CSRP3 | GOLT1B | MESDC1 | POLR3E | SLC51B | UNC13D |
| AKR1A1 | CST8 | GPATCH2L | MET | POM121 | SLC5A11 | UNC5A |
| ALAS2 | CSTF1 | GPM6B | METAP1D | POMT1 | SLC5A3 | UPF2 |
| ALDH1A2 | CTDP1 | GPR113 | METTL23 | PORCN | SLC6A1 | UPP1 |
| ALDH1A3 | CTDSP1 | GPS2 | MEX3A | PPAN-P2RY11 | SLC6A12 | UQCC2 |
| ALDOA | CTGF | GPSM1 | MFNG | PPAPDC1B | SLC6A2 | USF2 |
| ALDOC | CTHRC1 | GPSM2 | MFSD10 | PPARA | SLC6A7 | USP13 |
| ALOX5AP | CXCL12 | GRB10 | MFSD3 | PPCS | SLC7A9 | USP3 |
| AMER1 | CXCR4 | GRK6 | MGLL | PPL | SLC8A3 | USP31 |
| AMPD2 | CYB5R4 | GRK7 | MGME1 | PPM1E | SLC9A5 | USP37 |
| ANKRA2 | CYHR1 | GRTP1 | MGMT | PPP1CB | SLCO1A2 | USP4 |
| ANKRD16 | CYP26A1 | GSX2 | MGRN1 | PPP1R3B | SLCO5A1 | USP6NL |
| ANKRD33B | CYP4F22 | GTPBP3 | MICA | PPP1R3C | SLCO6A1 | UTP18 |
| ANO8 | CYSRT1 | GYS1 | MICAL2 | PPP2R3B | SLIT1 | UTS2R |
| AP1M1 | DAG1 | H2BFWT | MID1IP1 | PPP2R5C | SLITRK4 | VAPB |
| AP1S2 | DAGLA | HAAO | MIF | PPRC1 | SLITRK5 | VASP |
| AP3M1 | DAP | HAGHL | MIF4GD | PPT2 | SLK | VEGFA |
| AP5B1 | DCAF4 | HAMP | MINA | PRADC1 | SLMO1 | VEGFB |
| AP5Z1 | DCHS2 | HAPLN1 | MKLN1 | PRDM1 | SLTM | VHL |
| APEX1 | DDIT4 | HBEGF | MMP2 | PRDM13 | SMAD3 | VIM |
| APH1B | DDX4 | HCN1 | MOCS3 | PRDM8 | SMAD9 | VPS16 |
| AQP12A | DEF8 | HDAC3 | MORF4L1 | PRDX4 | SMC1B | VPS36 |
| AR | DEFB127 | HERC1 | MOS | PREP | SMIM11 | VPS53 |
| ARFRP1 | DERL1 | HERPUD1 | MOV10L1 | PRIMA1 | SMIM22 | VPS54 |
| ARHGAP8 | DERL3 | HES4 | MPL | PRKAA2 | SMPDL3B | WAC |
| ARHGDIG | DHCR7 | HHIP | MPP5 | PRKAR2A | SMYD2 | WASF3 |
| ARHGEF7 | DHRS13 | HIAT1 | MPST | PRKRIP1 | SNAP29 | WDR1 |
| ARL5B | DHRS7C | HIF1A | MRAS | PRMT1 | SNCB | WDR12 |
| ARMC6 | DIEXF | HIF3A | MROH1 | PROC | SNRPB | WDR36 |
| ARNT | DIS3L | HIGD1A | MROH5 | PROK2 | SNX12 | WEE1 |
| ARNT2 | DIS3L2 | HIRA | MRPL21 | PRR7 | SNX14 | WRNIP1 |
| ARNTL | DLL3 | HIVEP1 | MRPL22 | PRRC1 | SNX5 | XPC |
| ARPC2 | DLL4 | HK1 | MRPL40 | PSD | SOCS1 | XPNPEP1 |
| ARRDC3 | DMRT1 | HK2 | MRPL57 | PSMA1 | SOD2 | XPOT |
| ARX | DMRTC2 | HLA-E | MRPS30 | PSMA7 | SOD3 | XRN2 |
| ASAP3 | DMTN | HMGA1 | MRPS35 | PSMC3IP | SORCS3 | YBX1 |
| ASB3 | DNAH10 | HMGN2 | MRTO4 | PSMD3 | SORD | YEATS2 |
| ASB8 | DNAJB9 | HMGXB4 | MSL3 | PSMD5 | SOS1 | YPEL1 |
| ASCL1 | DNAJC28 | HMOX1 | MTFR1L | PSME3 | SOX14 | ZBTB10 |
| ASPDH | DNAJC5 | HMOX2 | MTFR2 | PSMG3 | SOX2 | ZBTB14 |
| ASS1 | DNLZ | HNRNPA3 | MTHFD1 | PTBP1 | SPATA2 | ZBTB37 |
| ASTN2 | DNM3 | HNRNPH3 | MTIF3 | PTBP3 | SPATA4 | ZBTB47 |
| ASXL1 | DNMBP | HNRNPUL1 | MTMR3 | PTDSS1 | SPDYA | ZDBF2 |
| ATF1 | DOC2A | HOXA1 | MTX1 | PTEN | SPEN | ZFAND4 |
| ATG7 | DOT1L | HOXA2 | MUC1 | PTGDR2 | SPESP1 | ZFAND5 |
| ATL1 | DPH6 | HOXA5 | MUC2 | PTGES | SPG21 | ZFR |
| ATL2 | DPYSL2 | HOXA7 | MUC3A | PTGES3 | SPHK1 | ZIC2 |
| ATP1B3 | DRAM2 | HOXD1 | MXI1 | PTGES3L-AARSD1 | SPOP | ZIM2 |
| ATP2C2 | DSCAM | HPCAL4 | MXRA8 | PTGIS | SPRY1 | ZIM3 |
| ATP5G2 | DSCAML1 | HS3ST3B1 | MYBL1 | PTPN12 | SPTBN1 | ZNF124 |
| ATP7A | DST | HS3ST4 | MYCN | PTPRF | SRC | ZNF131 |
| AXL | DTNA | HSBP1 | MYL6B | PTPRJ | SRF | ZNF185 |
| B4GALNT2 | DUSP7 | HSPA9 | MYLK | PTRF | SRFBP1 | ZNF217 |
| B4GALNT4 | DUSP8 | HTATIP2 | MYNN | PUF60 | SRGAP1 | ZNF250 |
| B9D1 | DUSP9 | HTN1 | MYO19 | PUS7L | SRM | ZNF329 |
| BAHD1 | E2F3 | HTR6 | MYO5A | PYGB | SRP9 | ZNF341 |
| BAI3 | ECI2 | HTRA1 | N6AMT2 | QRICH1 | SRSF1 | ZNF345 |
| BARX2 | EDEM1 | HYAL2 | NADK2 | R3HCC1L | SRSF5 | ZNF358 |
| BAX | EDIL3 | HYAL3 | NAE1 | RAB10 | SRSF6 | ZNF44 |
| BCAR3 | EDN1 | IARS | NANS | RAB21 | SSBP2 | ZNF48 |
| BCAT1 | EDNRB | ICE1 | NAP1L1 | RAB33A | SSH1 | ZNF547 |
| BCL11A | EED | IER2 | NAP1L5 | RAB40AL | SSR1 | ZNF578 |
| BCL11B | EFCAB12 | IFNGR2 | NAPB | RAB40C | SSR4 | ZNF581 |
| BCL2 | EFR3B | IGF1R | NAT10 | RAB43 | SSTR1 | ZNF605 |
| BCL2L11 | EGFL6 | IGF2 | NAT6 | RAB6A | ST6GAL1 | ZNF652 |
| BCL6 | EGFL7 | IGF2BP1 | NAV2 | RABEPK | ST6GALNAC3 | ZNF662 |
| BECN1 | EGLN1 | IGF2BP3 | NBAS | RAC1 | ST7 | ZNF664-FAM101A |
| BEND2 | EGLN3 | IGFBP1 | NBL1 | RAMP2 | STAG3 | ZNF668 |
| BHLHA15 | EGR4 | IGFBP2 | NCAPH2 | RAPGEF5 | STARD13 | ZNF696 |
| BHLHE40 | EHBP1 | IGFBP3 | NCBP2 | RAPGEFL1 | STAT3 | ZNF775 |
| BHLHE41 | EIF1 | IKZF2 | NCOR2 | RARB | STAT5A | ZNF776 |
| BID | EIF2AK3 | IL11RA | NDNL2 | RASA2 | STC1 | ZNF778 |
| BIRC6 | EIF3K | IL1RAPL1 | NDUFAF6 | RASA4 | STEAP3 | ZNF785 |
| BMP2 | EIF4E | IL4R | NDUFS7 | RASA4B | STIP1 | ZNF790 |
| BMP4 | EIF4E3 | IL6ST | NEDD4 | RASGEF1A | STK31 | ZNF829 |
| BMP6 | EIF4G1 | ING3 | NELFCD | RASGEF1B | STMN1 | ZNF836 |
| BNC2 | ELOVL1 | ING5 | NEO1 | RBBP7 | STRADA | ZNF843 |
| BNIP2 | ELOVL6 | INPPL1 | NEU4 | RBBP9 | STX1B | ZNF879 |
| BNIP3 | ELP2 | INSM1 | NFIL3 | RBFOX1 | STYXL1 | ZNHIT6 |
| BRD2 | EMC1 | IP6K1 | NFKB2 | RBM38 | SV2B | ZSCAN12 |
| BRI3BP | EMC2 | IPO13 | NIPAL1 | RBM47 | SYNM | ZZZ3 |
| BRIX1 | EMILIN1 | IQSEC3 | NLGN3 | RBM7 | SYT15 |  |
| BRSK2 | EML1 | IRF2BP1 | NOC2L | RBMY1F | TACR2 |  |
| BSG | EN1 | IRF2BPL | NOL11 | RCC2 | TAF1D |  |
| BTBD3 | ENDOG | IRF3 | NOL8 | RCN1 | TAF3 |  |
| BUB3 | ENG | IRX2 | NOP56 | RCOR2 | TAF4B |  |
| C10orf12 | ENGASE | IRX4 | NOS2 | RECK | TAL1 |  |
| C10orf53 | ENO1 | ISLR2 | NPAS2 | RECQL | TATDN2 |  |
| C11orf31 | ENO4 | ISM1 | NPHS1 | REEP1 | TBC1D14 |  |
| C11ORF71 | ENPP6 | ITCH | NPL | RELL2 | TBC1D15 |  |
| C12orf49 | ENTPD7 | ITGB2 | NPR3 | RELT | TBC1D20 |  |
| C12ORF66 | EPB41L4B | ITIH1 | NR1D1 | REV1 | TBC1D8B |  |
| C12orf73 | EPHB3 | JADE1 | NR2F2 | REXO4 | TBKBP1 |  |
| C14ORF166 | EPO | JADE2 | NR3C1 | RFX4 | TBL1XR1 |  |
| C14orf80 | EPPIN-WFDC6 | JAKMIP3 | NR4A1 | RFX5 | TCEA1 |  |
| C16orf62 | EQTN | JMJD6 | NRBF2 | RGS16 | TCEANC |  |
| C17ORF102 | ERAS | JPH1 | NREP | RGS3 | TCEB3CL2 |  |
| C17orf58 | ERCC2 | JRKL | NRF1 | RHOT1 | TDG |  |
| C18orf25 | ERF | KCNE1 | NRG3 | RHOXF2 | TDRD5 |  |
| C19orf24 | ERG | KCNE4 | NRGN | RIMKLA | TEF |  |
| C19orf52 | ERLEC1 | KCNG3 | NRP1 | RIMS4 | TELO2 |  |
| C19ORF68 | ERLIN1 | KCNH5 | NSMF | RMND1 | TERT |  |
| C2CD4B | ERO1L | KCNJ15 | NT5DC2 | RNF111 | TET2 |  |
| C2orf47 | ESRRA | KCTD1 | NT5E | RNF165 | TEX14 |  |
| C2orf54 | ETS2 | KCTD15 | NTMT1 | RNF180 | TF |  |
| C3orf58 | EVA1B | KDELR3 | NTN3 | RNF220 | TFAP4 |  |
| C4orf48 | FAF2 | KDM2A | NTRK2 | RNF5 | TFDP3 |  |
| C5orf38 | FAM102A | KDM3A | NUDT1 | ROR2 | TFEB |  |
| C5orf49 | FAM110A | KDM4B | NUDT10 | RORA | TFF3 |  |
| C7orf49 | FAM13A | KHK | NUP98 | RP2 | TFPT |  |
| C7ORF60 | FAM13B | KIAA0226L | NXNL2 | RPGRIP1L | TFRC |  |
| C8orf33 | FAM151B | KIAA0232 | NXPH1 | RPIA | TGFB3 |  |
| CA9 | FAM156A | KIAA0586 | NXPH4 | RPL22 | THAP4 |  |
| CABIN1 | FAM160A1 | KIAA1598 | OAZ2 | RPL34 | THG1L |  |
| CACNA2D2 | FAM162A | KIAA1919 | OCLN | RPLP1 | TIAL1 |  |
| CALN1 | FAM162B | KIF22 | ODC1 | RPP25 | TIAM1 |  |
| CALU | FAM173A | KIF26A | OLFM1 | RPS21 | TICAM1 |  |
| CAND1 | FAM181B | KIF3A | OLIG3 | RQCD1 | TIGD2 |  |
| CAPN1 | FAM184B | KIF9 | ONECUT1 | RREB1 | TIMM10 |  |
| CARF | FAM206A | KLF11 | OR2A42 | RSPO4 | TIMM23B |  |
| CASK | FAM214B | KLF9 | OR2T4 | RSRC1 | TIMP2 |  |
| CASKIN1 | FAM216A | KLHDC3 | OR2T5 | RTN4R | TK1 |  |
| CAST | FAM47E | KLHL35 | OR2T6 | RTN4RL2 | TLE1 |  |
| CBFA2T3 | FAM53A | KLK10 | OR6B2 | RUNDC1 | TLE3 |  |
| CBWD5 | FAM92B | KLK3 | ORAI1 | RUNX1T1 | TLL1 |  |
| CBX8 | FAM98B | KLK7 | OSBP2 | RUSC1 | TLN1 |  |
| CCBE1 | FAM98C | KMT2E | OSBPL10 | RUVBL2 | TLR2 |  |
| CCDC138 | FARS2 | KPNB1 | OSER1 | RWDD2A | TLR6 |  |
| CCDC183 | FASTKD2 | KRBA1 | OVOL1 | RWDD4 | TMED9 |  |
| CCDC77 | FBL | KREMEN1 | OXGR1 | RXRB | TMEM106B |  |
| CCDC78 | FBXL19 | KRR1 | P2RX6 | S100A16 | TMEM117 |  |
| CCDC85B | FBXL2 | KRTAP10-4 | P3H3 | S1PR2 | TMEM123 |  |
| CCL23 | FBXO15 | KRTAP12-3 | P4HA1 | SAA2-SAA4 | TMEM132E |  |
| CCND1 | FBXO17 | KRTAP9-8 | P4HA2 | SAE1 | TMEM171 |  |
| CCND2 | FBXO18 | L1CAM | PABPC1 | SAMD1 | TMEM176B |  |
| CCNJ | FBXO27 | LCOR | PACS1 | SAMD11 | TMEM179 |  |
| CCR7 | FBXO5 | LDHA | PAK1IP1 | SARDH | TMEM186 |  |
| CD302 | FCGBP | LEP | PANX1 | SART1 | TMEM192 |  |
| CD63 | FCGRT | LEPREL1 | PARVA | SC5D | TMEM235 |  |
| CD74 | FCHSD2 | LHPP | PATZ1 | SCAMP2 | TMEM244 |  |
| CDC42EP2 | FFAR4 | LHX2 | PAXIP1 | SCAP | TMEM35 |  |
| CDH1 | FGF10 | LHX5 | PCDH19 | SCD | TMEM40 |  |
| CDK11A | FGF11 | LIG1 | PDE3A | SCPEP1 | TMEM41A |  |
| CDK17 | FGF17 | LILRA3 | PDE7A | SCYL1 | TMEM57 |  |
| CDKL1 | FGF2 | LIMA1 | PDE8A | SDC1 | TMEM63B |  |
| CDKN1A | FGF6 | LINGO1 | PDGFB | SDHB | TMTC1 |  |
| CDKN2D | FGF9 | LLGL1 | PDK2 | SDK2 | TNNI3 |  |
| CDSN | FGFR3 | LLGL2 | PDK3 | SEC22C | TOP3A |  |
| CECR1 | FGR | LMBRD1 | PDP1 | SEC31A | TP53AIP1 |  |
| CELF2 | FHL2 | LMX1A | PDP2 | SECISBP2 | TPBGL |  |
| CELSR3 | FHOD1 | LNP1 | PDXK | SEH1L | TPD52L2 |  |
| CENPF | FIP1L1 | LOC100129697 | PDXP | SELT | TPI1 |  |
| CEP104 | FKBP11 | LOC102288414 | PER1 | SEMA3E | TPM2 |  |
| CEP19 | FKBP3 | LOC400863 | PES1 | SEMA4B | TPPP3 |  |
| CEP89 | FLT1 | LOC554223 | PF4V1 | SEMA5A | TPSD1 |  |
| CERCAM | FMOD | LOC730183 | PFKFB3 | SEP15 | TRAPPC2L |  |
| CERK | FOSB | LOC79999 | PFKFB4 | SEPT10 | TRAPPC5 |  |
| CGREF1 | FOXK1 | LOX | PFKL | SEPT2 | TREH |  |
| CHAD | FOXO4 | LRP2BP | PFKM | SERPINE1 | TRIM3 |  |
| CHAMP1 | FRG1 | LRP8 | PFN1 | SERPINH1 | TRIM32 |  |
| CHFR | FSD1 | LRRC10 | PGAP2 | SETD2 | TRIM33 |  |

## Supplemental Table 4 – Mouse orthologs converted from supplemental table 2

| **Mouse orthologs for genes from table 2** | | | | | | |
| --- | --- | --- | --- | --- | --- | --- |
| Aarsd1 | Cmtr1 | Gca | Gm3250 | Orai1 | Rpl22 | Telo2 |
| Aatf | Cnnm4 | Gcc2 | Gm19402 | Osbp2 | Rpl34 | Tert |
| Abca2 | Cnpy3 | Gck | Krtap12-1 | Osbpl10 | Rplp1 | Tet2 |
| Abcb1a | Cntln | Gcm2 | Gm10142 | Oser1 | Rpp25 | Tex14 |
| Abcb6 | Cntn2 | Gcsh | Gm10100 | Ovol1 | Rps21 | Trf |
| Abcc4 | Coil | Gda | L1cam | Oxgr1 | Rreb1 | Tfap4 |
| Abcf1 | Col25a1 | Gdnf | Lcor | P2rx6 | Rspo4 | Tfdp1 |
| Abhd17c | Commd3 | Gfra2 | Ldha | P3h3 | Rsrc1 | Tfeb |
| Abi2 | Bmi1 | Ggact | Lep | P4ha1 | Rtn4r | Tff3 |
| Ace | Commd4 | Ghrhr | Lhpp | P4ha2 | Rtn4rl2 | Tfpt |
| Ace2 | Copz1 | Gid4 | Lhx2 | Pabpc1 | Rundc1 | Tfrc |
| Ackr1 | Coq9 | Gk | Lhx5 | Pacs1 | Runx1t1 | Tgfb3 |
| Acoxl | Cox4i2 | Glmn | Lig1 | Pak1ip1 | Rusc1 | Thap4 |
| Acp6 | Cp | Glrx5 | Pirb | Panx1 | Ruvbl2 | Thg1l |
| Acvr1b | Cpeb2 | Gls2 | Pira2 | Parva | Rwdd2a | Tial1 |
| Acy3 | Cplx2 | Gmcl1 | Lima1 | Patz1 | Rwdd4a | Tiam1 |
| Adal | Cplx3 | Gmfb | Lingo1 | Paxip1 | Rxrb | Ticam1 |
| Adam30 | Cpne2 | Gmppa | Llgl1 | Pcdh19 | S1pr2 | Tigd2 |
| Adamts14 | Cpne7 | Gmppb | Llgl2 | Pde3a | S100a16 | Timm10 |
| Adap1 | Creb3 | Gnai1 | Lmbrd1 | Pde7a | Saa4 | Timm23 |
| Adcy3 | Creb3l1 | Gnb2 | Lmx1a | Pde8a | Sae1 | Timp2 |
| Adig | Crem | Golga4 | Lnp1 | Pdgfb | Samd1 | Tk1 |
| Adk | Crmp1 | Golt1a | Lox | Pdk2 | Samd11 | Tle1 |
| Adm | Cry2 | Golt1b | Lrp2bp | Pdk3 | Sardh | Tle3 |
| Adm2 | Csmd3 | Gpatch2l | Lrp8 | Pdp1 | Sart1 | Tll1 |
| Adnp | Csnk1g1 | Gpm6b | Lrrc10 | Pdp2 | Sc5d | Tln1 |
| Ado | Csrp3 | Gps2 | Lrrc36 | Pdxk | Scamp2 | Tlr2 |
| Adprhl1 | Cst8 | Gpsm1 | Lrrc57 | Pdxp | Scap | Tlr6 |
| Adra1b | Cstf1 | Gpsm2 | Lrrk2 | Per1 | Scd3 | Tmed9 |
| Aen | Ctdp1 | Grb10 | Lrrtm1 | Pes1 | Scd4 | Tmem40 |
| Agap1 | Ctdsp1 | Grk6 | Ltbp1 | Pf4 | Scd1 | Tmem41a |
| Ago4 | Cthrc1 | Grtp1 | Ltbp4 | Pfkfb3 | Scd2 | Tmem63b |
| Agpat1 | Cxcl12 | G0s2 | Ly6h | Pfkfb4 | Scpep1 | Tmem106b |
| Agtr1b | Cxcr4 | Gsx2 | Ly75 | Pfkl | Scyl1 | Tmem117 |
| Aif1 | Cyb5r4 | Gtpbp3 | Lypd5 | Pfkm | Sdc1 | Tmem123 |
| Aifm3 | Cyhr1 | Gys1 | Mab21l3 | Pfn1 | Sdhb | Tmem132e |
| Ak3 | Cyp4f39 | Haao | Maff | Pgap2 | Sdk2 | Tmem171 |
| Ak4 | Cyp26a1 | Haghl | Maged1 | Pggt1b | Sec22c | Tmem176b |
| Ak9 | Cysrt1 | Hamp | Mamdc4 | Pgk1 | Sec31a | Tmem179 |
| Akap10 | Dag1 | Hapln1 | Manba | Pgm3 | Secisbp2 | Tmem186 |
| Akr1a1 | Dagla | Hbegf | Map1lc3b | Phactr3 | Seh1l | Tmem192 |
| Alas2 | Dap | Hcn1 | Map3k1 | Phf12 | Sema3e | Tmem235 |
| Aldh1a2 | Dcaf4 | Hdac3 | Map3k8 | Phlpp1 | Sema4b | Tmtc1 |
| Aldh1a3 | Dchs2 | Herc1 | Map3k13 | Phpt1 | Sema5a | Tnni3 |
| Aldoa | Ddit4 | Herpud1 | Map4k1 | Phyhd1 | Serpine1 | Top3a |
| Aldoc | Ddx4 | Hhip | Map6 | Phyhipl | Serpinh1 | Tpbgl |
| Alox5ap | Def8 | Hif1a | Map7d2 | Phykpl | Setd2 | Tpd52l2 |
| Amer1 | Derl1 | Hif3a | Mapk4 | Piga | Sez6l2 | Tpi1 |
| Ampd2 | Derl3 | Higd1a | Mapk7 | Pigp | Sf1 | Tpm2 |
| Ankra2 | Dhcr7 | Hira | Mapk15 | Pigw | Sft2d3 | Tppp3 |
| Ankrd16 | Dhrs7c | Hivep1 | Mapre2 | Pik3ca | Sfxn4 | Tpsab1 |
| Ankrd33b | Dhrs13 | Hk1 | Marcks | Pik3ip1 | Sgk1 | Trappc2l |
| Ano8 | Dis3l | Hk2 | Marcksl1 | Pik3r1 | Sh3bgr | Trappc5 |
| Ap1m1 | Dis3l2 | H2-D1 | Matk | Pim1 | Sh3yl1 | Treh |
| Ap1s2 | Dll3 | H2-K1 | Max | Pithd1 | Shcbp1 | Trim3 |
| Ap3m1 | Dll4 | H2-M1 | Mbp | Pitpnb | She | Trim32 |
| Ap5b1 | Dmrt1 | H2-M2 | Mbtps2 | Pitpnc1 | Shisa6 | Trim33 |
| Ap5z1 | Dmrtc2 | H2-M3 | Mc5r | Pitrm1 | Shroom4 | Trim71 |
| Apex1 | Dmtn | H2-M5 | Mcm8 | Pitx2 | Sik1 | Trip6 |
| Aph1b | Dnah10 | H2-Q1 | Me3 | Piwil1 | Sirt1 | Trit1 |
| Aqp12 | Dnajb9 | H2-Q10 | Mea1 | Pkm | Slc2a1 | Trmt6 |
| Ar | Dnajc5 | H2-Q2 | Med20 | Pla2g6 | Slc2a3 | Trpc4ap |
| Arfrp1 | Dnajc28 | H2-Q4 | Mef2c | Plag1 | Slc2a10 | Trpm5 |
| Arhgap8 | Dnlz | H2-Q6 | Megf6 | Plagl1 | Slc4a11 | Trpm7 |
| Arhgdig | Dnm3 | H2-Q7 | Meis2 | Plau | Slc5a3 | Tsga10ip |
| Arhgef7 | Dnmbp | H2-Q8 | Met | Plcd3 | Slc5a11 | Tsku |
| Arl5b | Doc2a | H2-T22 | Metap1d | Plcz1 | Slc6a1 | Tspan4 |
| Armc6 | Dot1l | H2-T23 | Mettl23 | Plekha6 | Slc6a2 | Tssk3 |
| Arnt | Dph6 | H2-T3 | Mex3a | Plekhg3 | Slc6a7 | Ttc3 |
| Arnt2 | Dpysl2 | H2-M10.1 | Mfng | Plekhh1 | Slc6a12 | Ttc39c |
| Arntl | Dram2 | H2-M10.3 | Mfsd3 | Plin5 | Slc7a9 | Ttll7 |
| Arpc2 | Dscam | H2-M10.2 | Mfsd10 | Pmp2 | Slc8a3 | Tubal3 |
| Arrdc3 | Dscaml1 | H2-M10.5 | Mgll | Pnldc1 | Slc9a5 | Tubb3 |
| Arx | Dst | H2-M10.4 | Mgme1 | Pnoc | Slc12a4 | Tubb6 |
| Asap3 | Dtna | H2-M9 | Mgmt | Poglut1 | Slc12a5 | Tuft1 |
| Asb3 | Dusp7 |  | Mgrn1 | Pola1 | Slc12a7 | Twist1 |
| Asb8 | Dusp8 | Hmga1 | Mill2 | Polr3e | Slc16a3 | Twist2 |
| Ascl1 | Dusp9 | Hmgn2 | Mical2 | Pom121 | Slc16a13 | Twsg1 |
| Aspdh | E2f3 | Hmgxb4 | Mid1ip1 | Pomt1 | Slc22a13 | Txlna |
| Ass1 | Eci2 | Hmox1 | Mif | Porcn | Slc22a23 | Txlng |
| Astn2 | Edem1 | Hmox2 | Mif4gd | Ppan | Slc25a12 | Tyrobp |
| Asxl1 | Edil3 | Hnrnpa3 | Mkln1 | Ppara | Slc25a36 | U2af2 |
| Atf1 | Edn1 | Hnrnph3 | Mmp2 | Ppcs | Slc25a39 | Uba1 |
| Atg7 | Ednrb | Hnrnpul1 | Mocs3 | Ppl | Slc25a43 | Uba2 |
| Atl1 | Eed | Hoxa1 | Morf4l1 | Ppm1e | Slc25a46 | Uba3 |
| Atl2 | Efcab12 | Hoxa2 | Mos | Ppp1cb | Slc26a10 | Uba5 |
| Atp1b3 | Efr3b | Hoxa5 | Mov10l1 | Ppp1r3b | Slc29a1 | Uba6 |
| Atp2c2 | Egfl6 | Hoxa7 | Mpl | Ppp1r3c | Slc35e1 | Uba7 |
| Atp7a | Egfl7 | Hoxd1 | Mpst | Ppp2r3d | Slc35e3 | Ube2z |
| Axl | Egln1 | Hpcal4 | Mras | Ppp2r5c | Slc36a1 | Ubr3 |
| B4galnt2 | Egln3 | Hs3st3b1 | Mroh1 | Pprc1 | Slc36a3 | Ubr4 |
| B4galnt4 | Egr4 | Hs3st4 | Mroh5 | Ppt2 | Slc37a4 | Ubxn10 |
| B9d1 | Ehbp1 | Hsbp1 | Mrpl21 | Pradc1 | Slc39a7 | Ubxn11 |
| Bahd1 | Eif1 | Hspa9 | Mrpl22 | Prdm1 | Slc43a1 | Uhrf1 |
| Barx2 | Eif2ak3 | Htatip2 | Mrpl40 | Prdm8 | Slc43a3 | Ulk3 |
| Bax | Eif3k | Htr6 | Mrpl57 | Prdm13 | Slc51b | Umodl1 |
| Bcar3 | Eif4e | Htra1 | Mrps30 | Prdx4 | Gm6614 | Unc5a |
| Bcat1 | Eif4e3 | Hyal2 | Mrps35 | Prep | Slco1a4 | Unc13d |
| Bcl2 | Eif4g1 | Hyal3 | Mrto4 | Prima1 | Slco1a6 | Upf2 |
| Bcl2l11 | Elovl1 | Ice1 | Msl3 | Prkaa2 | Slco1a1 | Upp1 |
| Bcl6 | Elovl6 | Ier2 | Mtfr1l | Prkar2a | Slco1a5 | Uqcc2 |
| Bcl11a | Elp2 | Ifngr2 | Mtfr2 | Prkrip1 | Gm5724 | Usf2 |
| Bcl11b | Emc1 | Igf1r | Mthfd1 | Prmt1 | Slco5a1 | Usp3 |
| Becn1 | Emc2 | Igf2 | Mtif3 | Proc | Slco6c1 | Usp4 |
| Bhlha15 | Emilin1 | Igf2bp1 | Mtmr3 | Prok2 | Slco6d1 | Usp6nl |
| Bhlhe40 | Eml1 | Igf2bp3 | Mtx1 | Prr7 | Slit1 | Usp13 |
| Bhlhe41 | En1 | Igfbp1 | Muc1 | Prrc1 | Slitrk4 | Usp31 |
| Bid | Endog | Igfbp2 | Muc2 | Psd | Slitrk5 | Usp37 |
| Birc6 | Eng | Igfbp3 | Muc3a | Psma1 | Slk | Utp18 |
| Bmp2 | Engase | Ikzf2 | Mxi1 | Psma7 | Sltm | Uts2r |
| Bmp4 | Eno1 | Il1rapl1 | Mxra8 | Psmc3ip | Smad3 | Vapb |
| Bmp6 | Eno4 | Il4ra | Mybl1 | Psmd3 | Smad9 | Vasp |
| Bnc2 | Enpp6 | Il6st | Mycn | Psmd5 | Smc1b | Vegfa |
| Bnip2 | Entpd7 | Il11ra1 | Myl6b | Psme3 | Smim11 | Vegfb |
| Bnip3 | Epb41l4b | Il11ra2 | Mylk | Psmg3 | Smim22 | Vhl |
| Brd2 | Ephb3 | Gm13305 | Mynn | Ptbp1 | Smpdl3b | Vim |
| Bri3bp | Epo | Ing3 | Myo5a | Ptbp3 | Smyd2 | Vps16 |
| Brix1 | Eppin | Ing5 | Myo19 | Ptdss1 | Snap29 | Vps36 |
| Brsk2 | Wfdc6b | Inppl1 | Nadk2 | Pten | Sncb | Vps53 |
| Bsg | Eqtn | Insm1 | Nae1 | Ptgdr2 | Snrpb | Vps54 |
| Btbd3 | Eras | Ip6k1 | Nans | Ptges | Snx5 | Wac |
| Bub3 | Ercc2 | Ipo13 | Nap1l1 | Ptges3 | Snx12 | Wasf3 |
| C2cd4b | Erf | Iqsec3 | Nap1l5 | Ptgis | Snx14 | Wdr1 |
| Gm1673 | Erg | Irf2bp1 | Napb | Ptpn12 | Socs1 | Wdr12 |
| 1700001L19Rik | Erlec1 | Irf2bpl | Nat10 | Ptprf | Sod2 | Wdr36 |
| 1110038F14Rik | Erlin1 | Irf3 | Nav2 | Ptprj | Sod3 | Wee1 |
| 1700024G13Rik | Esrra | Irx2 | Nbas | Puf60 | Sorcs3 | Wrnip1 |
| Gm5617 | Ets2 | Irx4 | Nbl1 | Pus7l | Sord | Xpc |
| 1190007I07Rik | Eva1b | Islr2 | Ncaph2 | Pygb | Sos1 | Xpnpep1 |
| 1810010H24Rik | Faf2 | Ism1 | Ncbp2 | Qrich1 | Sox2 | Xpot |
| 8030462N17Rik | Fam13a | Itch | Ncor2 | R3hcc1l | Sox14 | Xrn2 |
| Car9 | Fam13b | Itgb2 | Ndufaf6 | Rab6a | Spata2 | Ybx1 |
| Cabin1 | Fam47e | Itih1 | Ndufs7 | Rab10 | Spata4 | Yeats2 |
| Cacna2d2 | Fam53a | Jade1 | Nedd4 | Rab21 | Spdya | Ypel1 |
| Caln1 | Fam98b | Jade2 | Nelfcd | Rab33a | Spen | Zbtb10 |
| Calu | Fam98c | Jakmip3 | Neo1 | Rab40b | Spesp1 | Zbtb14 |
| Cand1 | Fam102a | Jmjd6 | Neu4 | Rab40c | Spg21 | Zbtb37 |
| Capn1 | Fam110a | Jph1 | Nfil3 | Rab43 | Sphk1 | Zfp651 |
| Carf | Fam151b | Jrkl | Nfkb2 | Rabepk | Spop | Zdbf2 |
| Cask | Tmem29 | Kcne1 | Nipal1 | Rac1 | Spry1 | Zfand4 |
| Caskin1 | Fam162a | Kcne4 | Nlgn3 | Ramp2 | Sptbn1 | Zfand5 |
| Cast | Fam162b | Kcng3 | Noc2l | Rapgef5 | Src | Zfr |
| Cbfa2t3 | Fam181b | Kcnh5 | Nol8 | Rapgefl1 | Srf | Zic2 |
| Cbwd1 | Fam184b | Kcnj15 | Nol11 | Rarb | Srfbp1 | Zfp811 |
| Cbx8 | Fam214b | Kctd1 | Nop56 | Rasa2 | Srgap1 | Zfp964 |
| Ccbe1 | Fam216a | Kctd15 | Nos2 | Rasa4 | Srm | Zfp963 |
| Ccdc77 | Fars2 | Kdelr3 | Npas2 | Rasgef1a | Srp9 | Zfp553 |
| Ccdc78 | Fastkd2 | Kdm2a | Nphs1 | Rasgef1b | Srsf1 | Zfp131 |
| Ccdc85b | Fbl | Kdm3a | Npl | Rbbp7 | Srsf5 | Zfp185 |
| Ccdc138 | Fbxl2 | Kdm4b | Npr3 | Rbbp9 | Srsf6 | Zfp217 |
| Ccdc183 | Fbxl19 | Khk | Nr1d1 | Rbfox1 | Ssbp2 | Zfp647 |
| Ccl6 | Fbxo5 | D5Ertd579e | Nr2f2 | Rbm7 | Ssh1 | Zfp329 |
| Ccl9 | Fbxo15 | 2700049A03Rik | Nr3c1 | Rbm38 | Ssr1 | Zfp341 |
| Ccnd1 | Fbxo17 | Kif3a | Nr4a1 | Rbm47 | Ssr4 | Zfp358 |
| Ccnd2 | Fbxo27 | Kif9 | Nrbf2 | Rcc2 | Sstr1 | Zfp605 |
| Ccnj | Fcgbp | Kif22 | Nrep | Rcn1 | St6gal1 | Zfp652 |
| Ccr7 | Fcgrt | Kif26a | Nrf1 | Rcor2 | St6galnac3 | Zfp668 |
| Cd63 | Fchsd2 | Klf9 | Nrg3 | Reck | St7 | Zfp775 |
| Cd74 | Ffar4 | Klf11 | Nrgn | Recql | Stag3 | Zfp26 |
| Cd302 | Fgf2 | Klhdc3 | Nrp1 | Reep1 | Stard13 | Zfp560 |
| Cdc42ep2 | Fgf6 | Klhl35 | Nsmf | Rell2 | Stat3 | Zfp763 |
| Cdh1 | Fgf9 | Klk1b22 | Nt5dc2 | Relt | Stat5a | Zfp790 |
| Cdk11b | Fgf10 | Klk1b9 | Nt5e | Rev1 | Stc1 | Zfp160 |
| Cdk17 | Fgf11 | Klk1b4 | Ntmt1 | Rexo4 | Steap3 | Zfp879 |
| Cdkl1 | Fgf17 | Klk1b3 | Ntn3 | Rfx4 | Stip1 | Znhit6 |
| Cdkn1a | Fgfr3 | Klk1 | Ntrk2 | Rfx5 | Stk31 | Zscan12 |
| Cdkn2d | Fgr | Klk1b27 | Nudt1 | Rgs3 | Stmn1 | Zzz3 |
| Cdsn | Fhl2 | Klk1b26 | Nudt10 | Rgs16 | Strada |  |
| Celf2 | Fhod1 | Klk1b16 | Nudt11 | Rhot1 | Stx1b |  |
| Celsr3 | Fip1l1 | Klk1b8 | Nup98 | Rhox2a | Styxl1 |  |
| Cenpf | Fkbp3 | Klk1b1 | Nxnl2 | Rhox4b | Sv2b |  |
| Cep19 | Fkbp11 | Klk1b5 | Nxph1 | Rhox4c | Synm |  |
| Cep89 | Flt1 | Klk1b24 | Nxph4 | Rhox4e | Syt15 |  |
| Cep104 | Fmod | Klk1b21 | Oaz2 | Rhox4f | Tacr2 |  |
| Cercam | Fosb | Klk1b11 | Ocln | Rhox4g | Taf1d |  |
| Cerk | Foxk1 | Klk7 | Odc1 | Rhox2d | Taf3 |  |
| Cgref1 | Foxo4 | Klk10 | Olfm1 | Rhox3f | Taf4b |  |
| Chad | Frg1 | Kmt2e | Olig3 | Rimkla | Tal1 |  |
| Champ1 | Fsd1 | Kpnb1 | Onecut1 | Rims4 | Tatdn2 |  |
| Chfr | Fto | Krba1 | Olfr47 | Rmnd1 | Tbc1d8b |  |
| Chmp2a | Fuom | Kremen1 | Olfr434 | Rnf5 | Tbc1d14 |  |
| Chmp4b | Fxyd5 | Krr1 | Olfr435 | Rnf111 | Tbc1d15 |  |
| Chrm2 | Fzd7 | Krtap9-1 | Olfr224 | Rnf165 | Tbc1d20 |  |
| Chrna3 | G6pc3 | Krtap9-3 | Olfr328 | Rnf180 | Tbkbp1 |  |
| Chtf18 | Gabrd | Gm11568 | Olfr330 | Rnf220 | Tbl1xr1 |  |
| Fntb | Gadd45b | Krtap9-5 | Olfr331 | Ror2 | Tcea1 |  |
| Cited2 | Galk2 | Gm11559 | Olfr325 | Rora | Tceanc |  |
| Clip3 | Galnt2 | Gm7138 | Olfr720 | Rp2 | Tdg |  |
| Clstn3 | Gapdh | Gm3233 | Olfr1415 | Rpgrip1l | Tdrd5 |  |
| Cluh | Gbe1 | Gm3238 | Olfr1416 | Rpia | Tef |  |

## Supplemental Table 5 – Differential gene expression table MHC719/+ vs wildtype

| Transcriptome analysis, differential regulated genes in the LV of MHC^719/+^ vs wildtype mice | | |
| --- | --- | --- |
| gene symbol | log2FC | pval |
| Mup20 | Inf | 2.11E-17 |
| Mup3 | Inf | 1.65E-08 |
| Mup21 | Inf | 1.20E-05 |
| Serpina1c | Inf | 2.15E-05 |
| Fabp1 | Inf | 1.26E-04 |
| Rag1 | Inf | 6.30E-04 |
| Mup11 | Inf | 2.16E-03 |
| Mup7 | Inf | 2.16E-03 |
| AK016280 | Inf | 1.42E-02 |
| Otc | Inf | 1.76E-02 |
| Ugt2b5 | Inf | 1.76E-02 |
| Azgp1 | Inf | 2.18E-02 |
| Ces3a | Inf | 2.18E-02 |
| Cyp3a11 | Inf | 2.18E-02 |
| Alb | 7.19 | 1.22E-24 |
| Gc | 5.24 | 5.53E-05 |
| Fgb | 4.91 | 1.75E-02 |
| Apoa1 | 2.74 | 1.39E-02 |
| Serpina3k | 2.19 | 5.16E-03 |
| Hspa1a | 2.18 | 3.99E-08 |
| Lrrn4 | 2.12 | 1.60E-02 |
| Hspa1b | 2.09 | 3.75E-08 |
| Zim1 | 2.06 | 3.12E-02 |
| Ttr | 1.75 | 4.51E-02 |
| Upk3b | 1.64 | 2.49E-03 |
| Uprt | 1.61 | 2.98E-02 |
| AK086006 | 1.51 | 2.43E-04 |
| AK159010 | 1.50 | 3.29E-04 |
| C2 | 1.40 | 3.93E-02 |
| Vgll2 | 1.37 | 2.54E-02 |
| Spon2 | 1.34 | 5.82E-03 |
| Lmbrd2 | 1.30 | 9.39E-03 |
| A630019I02Rik | 1.29 | 1.61E-04 |
| Lypd6 | 1.28 | 3.04E-02 |
| AK053225 | 1.25 | 2.19E-02 |
| Dusp5 | 1.24 | 8.97E-03 |
| Ubxn10 | 1.21 | 1.52E-03 |
| mt-tRNA-Leu | 1.19 | 4.12E-04 |
| Wnt5a | 1.17 | 3.31E-03 |
| AK080187 | 1.16 | 4.19E-03 |
| Slc9a2 | 1.14 | 1.03E-02 |
| Lrtm1 | 1.13 | 7.65E-04 |
| Acta2 | 1.12 | 8.80E-04 |
| Aqp4 | 1.11 | 3.77E-02 |
| Tnc | 1.10 | 2.44E-02 |
| Uhmk1 | 1.08 | 6.49E-03 |
| E030010A14Rik | 1.05 | 2.23E-03 |
| Trip11 | 1.04 | 3.37E-03 |
| Cenpf | 1.02 | 7.66E-03 |
| Aqp8 | 1.00 | 1.73E-02 |
| Ctgf | 1.00 | 2.92E-03 |
| AK157531 | 0.99 | 3.86E-02 |
| Tfrc | 0.99 | 3.85E-03 |
| Sgcd | 0.98 | 1.13E-02 |
| Ovol1 | 0.97 | 3.32E-02 |
| Ms4a6d | 0.97 | 3.35E-02 |
| Bmpr2 | 0.96 | 7.31E-03 |
| Siah2 | 0.95 | 1.06E-02 |
| Kcnd2 | 0.95 | 9.54E-03 |
| Mob1b | 0.93 | 2.25E-02 |
| AK152437 | 0.93 | 3.24E-02 |
| Pigf | 0.92 | 1.03E-02 |
| Cntd1 | 0.92 | 1.28E-02 |
| Mfap3l | 0.92 | 1.21E-02 |
| Ptpn4 | 0.92 | 2.04E-02 |
| Slfn3 | 0.92 | 3.76E-02 |
| Slc26a2 | 0.91 | 3.36E-02 |
| Prune2 | 0.91 | 7.29E-03 |
| AK030906 | 0.90 | 4.64E-02 |
| Socs4 | 0.90 | 4.30E-02 |
| Ythdc2 | 0.90 | 2.26E-02 |
| Cfl2 | 0.89 | 8.11E-03 |
| Egln3 | 0.87 | 1.05E-02 |
| Arntl | 0.87 | 3.45E-02 |
| C5ar1 | 0.86 | 4.67E-02 |
| Zfp809 | 0.85 | 1.87E-02 |
| Chml | 0.85 | 3.07E-02 |
| Zbed6 | 0.85 | 2.63E-02 |
| Zfp189 | 0.84 | 3.98E-02 |
| Txndc12 | 0.84 | 1.82E-02 |
| Tpmt | 0.84 | 4.58E-02 |
| Mal | 0.83 | 2.45E-02 |
| AK219268 | 0.83 | 1.86E-02 |
| Zfp420 | 0.83 | 4.88E-02 |
| Ctnna3 | 0.83 | 2.20E-02 |
| Sccpdh | 0.82 | 1.47E-02 |
| Emc2 | 0.82 | 1.52E-02 |
| Ubr5 | 0.82 | 1.46E-02 |
| Mmrn1 | 0.82 | 3.22E-02 |
| Prox1 | 0.82 | 2.51E-02 |
| Spna1 | 0.81 | 4.88E-02 |
| Pde3a | 0.81 | 2.06E-02 |
| Ccl9 | 0.81 | 4.58E-02 |
| Fam161b | 0.81 | 2.87E-02 |
| Klk1b22 | 0.80 | 3.98E-02 |
| Hsph1 | 0.80 | 1.87E-02 |
| Grm1 | 0.80 | 2.09E-02 |
| Kpna1 | 0.79 | 1.96E-02 |
| Rragd | 0.79 | 1.87E-02 |
| Epdr1 | 0.78 | 2.61E-02 |
| Dsc2 | 0.77 | 2.36E-02 |
| 4732471D19Rik | 0.77 | 3.80E-02 |
| Pggt1b | 0.77 | 4.00E-02 |
| Svep1 | 0.77 | 2.97E-02 |
| Fyttd1 | 0.77 | 2.36E-02 |
| Hectd2 | 0.76 | 3.79E-02 |
| Tmtc3 | 0.76 | 3.62E-02 |
| Olfr1033 | 0.76 | 4.97E-02 |
| Fem1c | 0.76 | 4.05E-02 |
| Pde1c | 0.76 | 2.50E-02 |
| Tspan12 | 0.76 | 2.72E-02 |
| 1500017E21Rik | 0.76 | 3.26E-02 |
| Zfp62 | 0.76 | 2.99E-02 |
| Rab21 | 0.75 | 2.77E-02 |
| Dynll1 | 0.75 | 3.10E-02 |
| Ppip5k2 | 0.75 | 2.68E-02 |
| Mtm1 | 0.74 | 4.28E-02 |
| Rgs2 | 0.74 | 3.45E-02 |
| Pla2g5 | 0.74 | 4.24E-02 |
| AK207499 | 0.74 | 3.41E-02 |
| Inpp4b | 0.74 | 4.92E-02 |
| Hmcn1 | 0.74 | 3.10E-02 |
| Uba3 | 0.74 | 3.14E-02 |
| Zfyve16 | 0.73 | 4.22E-02 |
| Nhlrc1 | 0.73 | 4.85E-02 |
| Nbeal1 | 0.73 | 4.10E-02 |
| Fsd1l | 0.72 | 3.40E-02 |
| Zfp617 | 0.72 | 4.55E-02 |
| Pcmtd1 | 0.71 | 3.52E-02 |
| Atrnl1 | 0.71 | 3.82E-02 |
| Nppb | 0.71 | 3.55E-02 |
| Lnpep | 0.71 | 4.18E-02 |
| Ankrd13c | 0.71 | 4.97E-02 |
| 2810474O19Rik | 0.70 | 3.97E-02 |
| AK213609 | 0.70 | 4.91E-02 |
| Cpxm2 | 0.70 | 4.05E-02 |
| Pdpr | 0.70 | 4.96E-02 |
| Slc4a4 | 0.70 | 4.53E-02 |
| Prkaa2 | 0.70 | 3.81E-02 |
| Btaf1 | 0.69 | 4.34E-02 |
| Sh3kbp1 | 0.69 | 4.08E-02 |
| Dmxl2 | 0.69 | 4.39E-02 |
| Slc3a1 | 0.69 | 4.25E-02 |
| Rock2 | 0.68 | 4.12E-02 |
| Lrrc3b | 0.68 | 4.75E-02 |
| AK220016 | 0.68 | 4.32E-02 |
| Nebl | 0.68 | 4.67E-02 |
| Actc1 | 0.67 | 4.36E-02 |
| Eif5 | 0.67 | 4.59E-02 |
| Stk39 | 0.67 | 4.74E-02 |
| Akap6 | 0.66 | 4.81E-02 |
| Prepl | 0.66 | 4.84E-02 |
| Ehd2 | -0.67 | 4.63E-02 |
| Tmem161a | -0.67 | 4.78E-02 |
| Gm12824 | -0.67 | 4.60E-02 |
| Atn1 | -0.67 | 4.54E-02 |
| Scrib | -0.68 | 4.89E-02 |
| Rabac1 | -0.68 | 4.53E-02 |
| Gltscr2 | -0.68 | 4.69E-02 |
| Ormdl3 | -0.68 | 4.80E-02 |
| Gigyf1 | -0.68 | 4.57E-02 |
| Ppp1r37 | -0.68 | 4.35E-02 |
| Adamts7 | -0.69 | 4.41E-02 |
| Tmem82 | -0.69 | 4.78E-02 |
| Gga1 | -0.69 | 4.30E-02 |
| Kcng2 | -0.69 | 3.94E-02 |
| Cdc42bpg | -0.69 | 4.48E-02 |
| Zfp740 | -0.69 | 4.24E-02 |
| Dctn1 | -0.69 | 3.84E-02 |
| Phldb1 | -0.69 | 3.85E-02 |
| Atxn7l3 | -0.69 | 4.65E-02 |
| N4bp3 | -0.70 | 4.91E-02 |
| Cxxc5 | -0.70 | 4.95E-02 |
| Arf3 | -0.70 | 3.94E-02 |
| Ltbr | -0.70 | 4.26E-02 |
| Pcdhga1 | -0.70 | 3.83E-02 |
| Lrch4 | -0.70 | 4.79E-02 |
| Dmpk | -0.70 | 3.56E-02 |
| Lrg1 | -0.70 | 4.57E-02 |
| Pcdhga4 | -0.70 | 3.73E-02 |
| Igsf8 | -0.70 | 4.04E-02 |
| Jag2 | -0.70 | 3.80E-02 |
| Pcdhga7 | -0.70 | 3.68E-02 |
| Pcdhgb8 | -0.70 | 3.67E-02 |
| Fam109a | -0.71 | 4.77E-02 |
| Pcdhga10 | -0.71 | 3.62E-02 |
| D630003M21Rik | -0.71 | 4.50E-02 |
| Pcdhga6 | -0.71 | 3.61E-02 |
| Sardh | -0.71 | 4.73E-02 |
| Pcdhgb6 | -0.71 | 3.60E-02 |
| Pcdhgb7 | -0.71 | 3.55E-02 |
| 2310003H01Rik | -0.71 | 3.89E-02 |
| Vwa1 | -0.71 | 4.21E-02 |
| Cul9 | -0.71 | 3.80E-02 |
| Cited2 | -0.71 | 4.65E-02 |
| Sun2 | -0.71 | 3.37E-02 |
| Pcdhga2 | -0.71 | 3.42E-02 |
| Ykt6 | -0.72 | 3.52E-02 |
| Med16 | -0.72 | 3.53E-02 |
| Pcdhgb2 | -0.72 | 3.39E-02 |
| Pcdhgb4 | -0.72 | 3.38E-02 |
| AI464131 | -0.72 | 4.28E-02 |
| Cdc42ep1 | -0.72 | 3.39E-02 |
| Pcdhga12 | -0.72 | 3.28E-02 |
| Rbm42 | -0.72 | 3.76E-02 |
| Pcdhga5 | -0.72 | 3.27E-02 |
| Rhbdl3 | -0.72 | 4.69E-02 |
| Pcdhga11 | -0.72 | 3.20E-02 |
| Scarf1 | -0.72 | 3.55E-02 |
| 6430527G18Rik | -0.73 | 3.63E-02 |
| Obsl1 | -0.73 | 3.02E-02 |
| Gnb2 | -0.73 | 3.07E-02 |
| Pcdhgb1 | -0.73 | 3.12E-02 |
| Pcdhgb5 | -0.73 | 3.11E-02 |
| Acsf3 | -0.73 | 3.51E-02 |
| Hcfc1r1 | -0.73 | 3.22E-02 |
| Tsen34 | -0.73 | 3.47E-02 |
| Megf8 | -0.73 | 3.03E-02 |
| Pcdhga8 | -0.73 | 3.07E-02 |
| Rgl2 | -0.73 | 3.14E-02 |
| Pcdhga3 | -0.73 | 3.03E-02 |
| Pvrl2 | -0.73 | 3.96E-02 |
| Kbtbd5 | -0.73 | 3.62E-02 |
| Gsn | -0.73 | 2.74E-02 |
| Pcdhgc5 | -0.73 | 2.96E-02 |
| Pcdhga9 | -0.73 | 2.96E-02 |
| Smcr7 | -0.73 | 3.52E-02 |
| Hspa12b | -0.73 | 3.01E-02 |
| Lypla2 | -0.74 | 3.70E-02 |
| Pcdha4-g | -0.74 | 2.90E-02 |
| Cdk9 | -0.74 | 3.49E-02 |
| Wnk4 | -0.74 | 4.02E-02 |
| Dyrk1b | -0.74 | 3.69E-02 |
| Pcdhgc4 | -0.74 | 2.84E-02 |
| Bcl9l | -0.74 | 3.24E-02 |
| Zfp787 | -0.74 | 3.85E-02 |
| Tfeb | -0.74 | 3.23E-02 |
| Mllt6 | -0.74 | 2.66E-02 |
| Ptms | -0.75 | 2.71E-02 |
| Dapk3 | -0.75 | 3.61E-02 |
| Cyth1 | -0.75 | 2.72E-02 |
| Sh3tc1 | -0.75 | 3.25E-02 |
| Phf1 | -0.75 | 3.01E-02 |
| Trp53i13 | -0.75 | 4.16E-02 |
| Sod3 | -0.75 | 2.68E-02 |
| Gpx4 | -0.76 | 2.39E-02 |
| Fam195a | -0.76 | 2.68E-02 |
| Phactr1 | -0.76 | 3.35E-02 |
| Slc39a3 | -0.76 | 2.85E-02 |
| Ntn1 | -0.76 | 2.39E-02 |
| Cdkn2d | -0.76 | 4.57E-02 |
| Dohh | -0.76 | 2.70E-02 |
| Rhbdd3 | -0.76 | 2.94E-02 |
| Inha | -0.76 | 4.13E-02 |
| Gltscr1 | -0.76 | 3.13E-02 |
| Ssc5d | -0.76 | 4.19E-02 |
| Tead3 | -0.76 | 3.19E-02 |
| Arf5 | -0.77 | 2.47E-02 |
| Rusc2 | -0.77 | 2.39E-02 |
| AK179066 | -0.77 | 4.01E-02 |
| Tssc4 | -0.77 | 3.65E-02 |
| Nfatc4 | -0.77 | 4.80E-02 |
| Zfp358 | -0.77 | 2.48E-02 |
| Slc25a34 | -0.77 | 2.14E-02 |
| Camk2b | -0.77 | 3.88E-02 |
| Zdhhc18 | -0.77 | 2.42E-02 |
| C1qtnf1 | -0.77 | 2.42E-02 |
| Cldn5 | -0.78 | 2.81E-02 |
| Gpr137 | -0.78 | 2.70E-02 |
| Fam43a | -0.78 | 2.39E-02 |
| Zbtb7b | -0.78 | 2.77E-02 |
| Chmp6 | -0.78 | 2.97E-02 |
| Notch4 | -0.78 | 2.09E-02 |
| Dact3 | -0.79 | 3.12E-02 |
| Adprhl2 | -0.79 | 3.04E-02 |
| Fam131a | -0.79 | 2.79E-02 |
| Get4 | -0.79 | 2.24E-02 |
| C330006K01Rik | -0.79 | 3.10E-02 |
| Adora1 | -0.79 | 2.80E-02 |
| Wisp2 | -0.79 | 2.78E-02 |
| Prr13 | -0.79 | 3.02E-02 |
| Ddr1 | -0.79 | 2.15E-02 |
| Agpat1 | -0.79 | 1.97E-02 |
| Inmt | -0.80 | 3.40E-02 |
| Tcap | -0.80 | 1.68E-02 |
| Sdc3 | -0.80 | 1.74E-02 |
| Kif26a | -0.80 | 1.96E-02 |
| Lemd2 | -0.80 | 1.95E-02 |
| Bcl9 | -0.80 | 2.34E-02 |
| Chst7 | -0.80 | 4.88E-02 |
| Abtb1 | -0.80 | 2.14E-02 |
| Mnt | -0.80 | 2.46E-02 |
| Pex6 | -0.81 | 1.79E-02 |
| Pim1 | -0.81 | 2.90E-02 |
| Csf2ra | -0.81 | 3.28E-02 |
| 3110056O03Rik | -0.81 | 2.10E-02 |
| Tjap1 | -0.81 | 1.85E-02 |
| Cbx6 | -0.81 | 1.64E-02 |
| Podn | -0.82 | 1.91E-02 |
| Ptk7 | -0.82 | 4.21E-02 |
| Il3ra | -0.82 | 3.88E-02 |
| Ptp4a3 | -0.82 | 1.45E-02 |
| Mir208b | -0.82 | 1.69E-02 |
| Foxo4 | -0.82 | 1.63E-02 |
| Kcnk3 | -0.83 | 1.34E-02 |
| Tbx3 | -0.83 | 2.10E-02 |
| Paqr7 | -0.83 | 2.24E-02 |
| Agfg2 | -0.83 | 2.43E-02 |
| Myh7b | -0.83 | 1.28E-02 |
| Relb | -0.84 | 2.36E-02 |
| Atp5d | -0.84 | 1.23E-02 |
| Megf6 | -0.84 | 3.03E-02 |
| Hic1 | -0.84 | 1.92E-02 |
| 3110082I17Rik | -0.84 | 3.81E-02 |
| Mlst8 | -0.84 | 2.05E-02 |
| AK039023 | -0.84 | 1.66E-02 |
| Dmwd | -0.84 | 1.33E-02 |
| Pex14 | -0.85 | 1.91E-02 |
| Tbxa2r | -0.85 | 3.54E-02 |
| B4galt2 | -0.85 | 3.59E-02 |
| Ddit4 | -0.85 | 1.32E-02 |
| Chpf | -0.85 | 1.43E-02 |
| C1qtnf6 | -0.85 | 1.78E-02 |
| Unc5b | -0.85 | 1.25E-02 |
| Poll | -0.85 | 2.44E-02 |
| C130074G19Rik | -0.85 | 1.31E-02 |
| Zfp865 | -0.85 | 1.69E-02 |
| Tcf3 | -0.86 | 1.40E-02 |
| Rhbdl1 | -0.86 | 3.16E-02 |
| Kcne1 | -0.86 | 1.62E-02 |
| Hmcn2 | -0.86 | 1.68E-02 |
| Sox18 | -0.86 | 1.10E-02 |
| AK196542 | -0.86 | 1.62E-02 |
| Map3k10 | -0.86 | 1.60E-02 |
| Mid1ip1 | -0.86 | 1.04E-02 |
| Rn45s | -0.87 | 9.06E-03 |
| AK205023 | -0.87 | 2.24E-02 |
| Kcp | -0.88 | 2.13E-02 |
| Vasp | -0.88 | 1.54E-02 |
| Fam19a5 | -0.88 | 2.91E-02 |
| Cdo1 | -0.88 | 3.37E-02 |
| Mapk8ip1 | -0.88 | 2.34E-02 |
| Zfp219 | -0.88 | 1.05E-02 |
| 6030429G01Rik | -0.89 | 2.19E-02 |
| Cited4 | -0.89 | 1.61E-02 |
| Irf2bp1 | -0.89 | 1.14E-02 |
| Ppil1 | -0.89 | 1.08E-02 |
| Gm13375 | -0.89 | 1.83E-02 |
| Tfpt | -0.89 | 4.39E-02 |
| Zfp771 | -0.90 | 1.25E-02 |
| Rnf126 | -0.90 | 1.16E-02 |
| Hip1r | -0.90 | 9.96E-03 |
| Cby1 | -0.90 | 1.98E-02 |
| Exoc3l | -0.90 | 1.62E-02 |
| Mib2 | -0.90 | 8.46E-03 |
| Tbx2 | -0.90 | 1.47E-02 |
| Grik5 | -0.90 | 1.43E-02 |
| Prrt4 | -0.90 | 2.30E-02 |
| Aldh16a1 | -0.91 | 1.12E-02 |
| Lrfn4 | -0.91 | 1.19E-02 |
| Lfng | -0.91 | 1.62E-02 |
| Fam117a | -0.91 | 2.12E-02 |
| Tmub1 | -0.91 | 2.09E-02 |
| Sbk2 | -0.91 | 2.27E-02 |
| Ephb3 | -0.92 | 9.35E-03 |
| Mfsd12 | -0.92 | 2.52E-02 |
| Isoc2a | -0.92 | 1.29E-02 |
| Art5 | -0.92 | 1.18E-02 |
| Zfp428 | -0.93 | 2.02E-02 |
| Cops6 | -0.93 | 6.68E-03 |
| Cirbp | -0.93 | 8.58E-03 |
| mmu-mir-805 | -0.93 | 9.83E-03 |
| Col16a1 | -0.93 | 1.36E-02 |
| AK035229 | -0.93 | 1.64E-02 |
| Sema6b | -0.94 | 9.11E-03 |
| Gtpbp5 | -0.94 | 9.46E-03 |
| Trib3 | -0.94 | 3.60E-02 |
| Gramd1b | -0.94 | 1.04E-02 |
| Pitpnm1 | -0.94 | 6.57E-03 |
| Eln | -0.94 | 6.19E-03 |
| Trabd | -0.95 | 5.99E-03 |
| Kbtbd13 | -0.95 | 4.28E-02 |
| Hcn4 | -0.95 | 2.16E-02 |
| Tmem132a | -0.95 | 7.50E-03 |
| Ppfia4 | -0.95 | 3.05E-02 |
| Ccdc61 | -0.96 | 2.81E-02 |
| Junb | -0.96 | 1.15E-02 |
| Plekhh3 | -0.96 | 7.29E-03 |
| Pamr1 | -0.96 | 2.13E-02 |
| Ptpru | -0.96 | 1.12E-02 |
| Tbc1d2 | -0.96 | 4.12E-02 |
| 9930021D14Rik | -0.97 | 1.09E-02 |
| Frs3 | -0.97 | 2.30E-02 |
| Ccdc85b | -0.97 | 5.74E-03 |
| AK192600 | -0.97 | 4.44E-02 |
| Klhdc8b | -0.98 | 6.44E-03 |
| Engase | -0.98 | 1.62E-02 |
| Nfkbil1 | -0.98 | 2.27E-02 |
| Rasip1 | -0.98 | 3.92E-03 |
| E2f1 | -0.99 | 3.53E-02 |
| Fbn2 | -0.99 | 3.60E-02 |
| Fam181b | -1.00 | 4.14E-02 |
| 6030419C18Rik | -1.00 | 7.31E-03 |
| Mier2 | -1.00 | 6.18E-03 |
| Pim3 | -1.01 | 2.85E-03 |
| Ccdc9 | -1.01 | 5.10E-03 |
| Irx2 | -1.01 | 7.18E-03 |
| Cpt1c | -1.02 | 1.95E-02 |
| Itga2b | -1.03 | 4.04E-02 |
| Gm1337 | -1.03 | 4.49E-02 |
| Phgdh | -1.03 | 4.14E-02 |
| Ano8 | -1.03 | 3.31E-03 |
| C1qtnf4 | -1.04 | 3.74E-02 |
| Cadm4 | -1.04 | 5.07E-03 |
| Gadd45b | -1.04 | 1.59E-02 |
| Mamstr | -1.05 | 1.98E-02 |
| Acbd4 | -1.05 | 2.87E-03 |
| Gadd45g | -1.05 | 1.00E-02 |
| Casq1 | -1.05 | 2.29E-03 |
| Arhgap33 | -1.06 | 3.62E-02 |
| Gpr162 | -1.06 | 2.01E-02 |
| Lrrn2 | -1.06 | 8.53E-03 |
| Dqx1 | -1.06 | 4.36E-02 |
| AK178359 | -1.07 | 5.47E-03 |
| Scara3 | -1.07 | 1.05E-02 |
| Zfp579 | -1.08 | 4.11E-03 |
| Gm11747 | -1.08 | 6.55E-03 |
| AK213749 | -1.09 | 4.03E-03 |
| Irx5 | -1.09 | 6.72E-03 |
| Rrad | -1.09 | 1.24E-03 |
| AK185715 | -1.09 | 4.34E-02 |
| Nrgn | -1.10 | 1.69E-02 |
| Car3 | -1.10 | 4.60E-03 |
| AK016007 | -1.10 | 2.84E-02 |
| Ache | -1.11 | 4.45E-03 |
| Lrrc55 | -1.11 | 3.95E-02 |
| Numbl | -1.13 | 3.48E-03 |
| Foxo6 | -1.14 | 2.24E-02 |
| Shisa4 | -1.14 | 2.61E-02 |
| Clec3b | -1.15 | 1.13E-03 |
| Abca4 | -1.15 | 1.80E-03 |
| Prrt2 | -1.18 | 3.62E-03 |
| AK040202 | -1.18 | 1.64E-02 |
| Ramp1 | -1.18 | 3.26E-03 |
| Irx1 | -1.20 | 3.75E-03 |
| F2rl3 | -1.20 | 2.23E-02 |
| Slc35e4 | -1.21 | 2.52E-03 |
| Sox10 | -1.21 | 1.73E-02 |
| Bhlhe41 | -1.22 | 1.29E-03 |
| Wnt9b | -1.22 | 1.63E-02 |
| Sema6c | -1.23 | 2.02E-03 |
| AK213404 | -1.24 | 2.86E-03 |
| Col12a1 | -1.24 | 6.27E-03 |
| Stard10 | -1.25 | 3.61E-04 |
| Lzts2 | -1.26 | 2.47E-04 |
| Aspdh | -1.26 | 1.40E-02 |
| Pdlim4 | -1.27 | 1.57E-03 |
| Tmem238 | -1.27 | 2.41E-02 |
| Slc52a3 | -1.27 | 4.46E-02 |
| Ier5l | -1.28 | 1.58E-03 |
| Nrtn | -1.29 | 3.53E-04 |
| AK148461 | -1.30 | 2.15E-02 |
| Islr2 | -1.32 | 4.35E-02 |
| Tfap4 | -1.33 | 1.01E-02 |
| S1pr4 | -1.34 | 1.40E-02 |
| Stk32b | -1.34 | 1.68E-02 |
| Cntfr | -1.36 | 1.95E-03 |
| Fbxl19 | -1.36 | 1.12E-03 |
| Mxd4 | -1.37 | 9.88E-05 |
| Angptl7 | -1.39 | 1.62E-04 |
| Olfr39 | -1.39 | 1.39E-02 |
| Snai3 | -1.39 | 1.12E-03 |
| Zfp503 | -1.41 | 9.87E-03 |
| Cacna1h | -1.41 | 6.73E-04 |
| AK190918 | -1.41 | 2.22E-03 |
| Kirrel3 | -1.42 | 5.76E-03 |
| Clcf1 | -1.42 | 1.85E-03 |
| Sox4 | -1.43 | 5.07E-05 |
| Sfrp2 | -1.43 | 4.19E-03 |
| Tmem158 | -1.44 | 1.50E-02 |
| Bbc3 | -1.45 | 2.57E-03 |
| Lect1 | -1.45 | 2.64E-03 |
| AK007459 | -1.46 | 9.79E-05 |
| Tnni1 | -1.46 | 2.67E-02 |
| Clec2l | -1.47 | 4.08E-02 |
| Clcn1 | -1.48 | 2.03E-04 |
| Fam171a2 | -1.51 | 1.02E-04 |
| Cacna1g | -1.52 | 1.76E-05 |
| Gm9934 | -1.55 | 3.97E-03 |
| Irx3 | -1.57 | 4.90E-05 |
| Izumo1 | -1.60 | 2.32E-02 |
| Coch | -1.60 | 2.95E-02 |
| Drd2 | -1.61 | 2.91E-05 |
| Matn4 | -1.61 | 2.47E-02 |
| Sema4f | -1.61 | 4.85E-02 |
| 6430598A04Rik | -1.62 | 4.87E-03 |
| Shox2 | -1.65 | 3.54E-02 |
| Cidec | -1.65 | 1.59E-03 |
| Col8a2 | -1.66 | 4.60E-03 |
| Camk1g | -1.70 | 2.07E-02 |
| Gm6498 | -1.73 | 6.91E-03 |
| LOC319574 | -1.76 | 2.21E-02 |
| 2410018M08Rik | -1.78 | 3.44E-05 |
| Hamp | -1.80 | 5.29E-03 |
| Ypel4 | -1.80 | 2.21E-02 |
| Col11a2 | -1.81 | 9.29E-05 |
| Olfr873 | -1.84 | 3.34E-02 |
| Comp | -1.84 | 2.46E-06 |
| Uts2r | -1.87 | 1.62E-02 |
| Plin1 | -1.88 | 5.92E-04 |
| Mir5109 | -1.90 | 1.71E-03 |
| Troap | -1.93 | 4.55E-02 |
| Cyp2e1 | -1.94 | 7.22E-06 |
| Epn3 | -1.95 | 1.61E-06 |
| Dkk3 | -1.96 | 4.32E-08 |
| Cntn2 | -1.97 | 3.17E-08 |
| Gpr27 | -1.98 | 1.16E-05 |
| Cacna2d2 | -1.99 | 3.90E-07 |
| Sfrp5 | -2.00 | 2.27E-05 |
| Fgf12 | -2.01 | 2.12E-03 |
| Cfd | -2.04 | 8.50E-07 |
| Ifltd1 | -2.10 | 1.88E-05 |
| Gata5 | -2.12 | 1.56E-02 |
| 4930413G21Rik | -2.15 | 2.15E-04 |
| Gm1078 | -2.19 | 1.14E-08 |
| Gm12522 | -2.24 | 4.90E-03 |
| Sln | -2.25 | 6.26E-09 |
| Oprd1 | -2.25 | 1.63E-03 |
| Lars2 | -2.33 | 2.33E-11 |
| Ppp1r1b | -2.41 | 1.71E-05 |
| Mir466d | -2.44 | 4.91E-05 |
| Pnmt | -2.48 | 5.34E-03 |
| Cpne5 | -2.48 | 4.65E-09 |
| Prss35 | -2.51 | 5.78E-04 |
| AK155692 | -2.52 | 3.47E-10 |
| Epyc | -2.54 | 1.89E-02 |
| Myl4 | -2.55 | 1.35E-12 |
| Chad | -2.68 | 1.12E-05 |
| Unc80 | -2.77 | 7.55E-05 |
| AK144265 | -2.78 | 4.26E-14 |
| 1700073E17Rik | -2.83 | 4.61E-04 |
| Myl7 | -2.84 | 1.55E-14 |
| Mybphl | -2.86 | 1.11E-11 |
| Fzd10 | -2.90 | 1.71E-02 |
| Fmod | -2.93 | 1.44E-13 |
| Gcgr | -2.97 | 7.76E-06 |
| Bpifb4 | -3.04 | 1.02E-10 |
| Vat1l | -3.19 | 2.27E-02 |
| Cilp2 | -3.21 | 3.17E-08 |
| Gpr83 | -3.24 | 4.63E-02 |
| Tssk6 | -3.24 | 4.63E-02 |
| 4833424O15Rik | -3.26 | 1.82E-02 |
| Nfasc | -3.32 | 3.99E-05 |
| AK039372 | -3.86 | 2.48E-02 |
| Mir1943 | -3.90 | 2.18E-02 |
| Ucma | -3.98 | 1.69E-02 |
| 1700003D09Rik | -4.04 | 4.16E-04 |
| Clec3a | -4.54 | 1.63E-03 |
| Rxfp3 | -4.82 | 1.42E-02 |
| BC049631 | -5.09 | 5.24E-03 |
| Gjd3 | -5.19 | 3.42E-03 |
| Sftpb | -6.59 | 1.94E-07 |
| Scgb1a1 | -Inf | 2.65E-13 |
| Vsnl1 | -Inf | 1.12E-06 |
| Sftpc | -Inf | 3.98E-03 |
| Sec14l3 | -Inf | 5.50E-03 |
| AK006943 | -Inf | 1.46E-02 |
| Sftpa1 | -Inf | 1.46E-02 |
| Foxq1 | -Inf | 2.39E-02 |
| Rasgrf1 | -Inf | 3.93E-02 |
| Spatc1 | -Inf | 4.64E-02 |

## Supplemental Table 6 – differential regulated proteins MHC^719/+^ vs wildtype mice

| Proteome analysis, differential regulated proteins in the LV of MHC^719/+^ vs wildtype mice | | | |
| --- | --- | --- | --- |
| protein_accession | **Gene symbol** | **log2FC** | **pval** |
| P09541 | Myl4 | 2.50 | 1.57E-11 |
| P05125 | Nppa | 2.24 | 5.80E-06 |
| Q9QVP4 | Myl7 | 2.07 | 2.19E-07 |
| P05977 | Myl1 | 1.91 | 3.66E-06 |
| Q9DB29 | Iah1 | 1.67 | 1.04E-04 |
| Q62000 | Ogn | 1.44 | 1.08E-13 |
| Q99MQ4 | Aspn | 1.33 | 2.62E-09 |
| Q8CIZ8 | Vwf | 1.31 | 9.46E-03 |
| P20152;P03995;P46660;P08551;P08553 | Vim | 1.29 | 0.00E+00 |
| P28654 | Dcn | 1.27 | 1.16E-11 |
| Q62009;CON__Q2KJC7 | Postn | 1.16 | 3.63E-10 |
| Q9WVH9 | Fbln5 | 1.16 | 4.64E-08 |
| Q9CRB6 | Tppp3 | 1.15 | 2.84E-04 |
| Q91W90 | Txndc5 | 1.11 | 4.44E-04 |
| Q00915 | Rbp1 | 1.11 | 1.18E-03 |
| Q62188 | Dpysl3 | 1.07 | 9.89E-13 |
| Q9JK53 | Prelp | 1.06 | 9.45E-11 |
| O89053 | Coro1a | 1.03 | 5.29E-03 |
| Q9CX80 | Cygb | 1.01 | 7.38E-04 |
| P51885 | Lum | 1.00 | 1.27E-07 |
| Q9QZJ6 | Mfap5 | 0.99 | 3.41E-03 |
| Q61206 | Pafah1b2 | 0.99 | 1.02E-04 |
| Q06890 | Clu | 0.91 | 2.77E-09 |
| Q9D8Y0;Q9D4J1 | Efhd2 | 0.89 | 9.16E-05 |
| P46412;P21765 | Gpx3 | 0.89 | 6.36E-04 |
| Q62523 | Zyx | 0.88 | 2.82E-07 |
| Q9R0P9 | Uchl1 | 0.87 | 5.80E-06 |
| Q02819 | Nucb1 | 0.87 | 2.80E-04 |
| Q9CQ89 | Cuta | 0.87 | 1.10E-03 |
| P24549;O35945 | Aldh1a1 | 0.84 | 8.49E-06 |
| P28653 | Bgn | 0.83 | 5.36E-04 |
| Q8K4G1 | Ltbp4 | 0.83 | 3.72E-03 |
| Q62465 | Vat1 | 0.81 | 6.70E-03 |
| O70318 | Epb41l2 | 0.79 | 6.83E-03 |
| Q9R0P3 | Esd | 0.79 | 1.12E-07 |
| Q8BFW7 | Lpp | 0.77 | 2.27E-04 |
| Q9WVA4;Q9R1Q8 | Tagln2 | 0.76 | 1.20E-14 |
| Q9CWF2 | Tubb2b | 0.75 | 9.24E-06 |
| Q91Z83 | Myh7 | 0.74 | 2.32E-09 |
| P50543 | S100a11 | 0.74 | 9.31E-03 |
| Q04447 | Ckb | 0.74 | 5.91E-14 |
| P28798 | Grn | 0.73 | 8.87E-04 |
| Q01149 | Col1a2 | 0.73 | 6.19E-03 |
| P10126 | Eef1a1 | 0.73 | 8.83E-08 |
| Q9QZ57 | Hspb3 | 0.73 | 1.14E-03 |
| O08553 | Dpysl2 | 0.72 | 0.00E+00 |
| P08074 | Cbr2 | 0.72 | 7.91E-03 |
| Q99KC8 | Vwa5a | 0.72 | 1.15E-04 |
| P29788 | Vtn | 0.71 | 3.26E-04 |
| P07901 | Hsp90aa1 | 0.70 | 3.48E-12 |
| P09103 | P4hb | 0.70 | 0.00E+00 |
| Q7TPR4 | Actn1 | 0.70 | 1.18E-10 |
| Q00896;P07758 | Serpina1c;Serpina1a | 0.70 | 0.00E+00 |
| P11087 | Col1a1 | 0.70 | 1.35E-03 |
| P68510 | Ywhah | 0.69 | 2.14E-08 |
| O55131;Q8C650 | Sept7 | 0.69 | 6.96E-09 |
| Q9JII6 | Akr1a1 | 0.69 | 4.61E-10 |
| P10107 | Anxa1 | 0.69 | 2.55E-06 |
| Q91VJ2 | Prkcdbp | 0.68 | 1.43E-03 |
| Q61937 | Npm1 | 0.68 | 1.73E-03 |
| Q9QZZ6 | Dpt | 0.67 | 3.11E-03 |
| Q9DCN2 | Cyb5r3 | 0.67 | 3.72E-11 |
| Q9EQK5 | Mvp | 0.67 | 2.55E-05 |
| Q8BZF8 | Pgm5 | 0.67 | 4.44E-16 |
| P07356;REV__Q8BND4 | Anxa2 | 0.67 | 0.00E+00 |
| P68254 | Ywhaq | 0.66 | 1.80E-03 |
| P06909 | Cfh | 0.65 | 2.21E-05 |
| Q9D379 | Ephx1 | 0.65 | 9.02E-04 |
| Q8BWB1 | Synpo2l | 0.65 | 1.95E-10 |
| P07759 | Serpina3k | 0.65 | 0.00E+00 |
| P97447 | Fhl1 | 0.65 | 2.57E-06 |
| Q9D7X3 | Dusp3 | 0.65 | 8.85E-05 |
| P22599;Q00898 | Serpina1b | 0.64 | 3.17E-10 |
| Q6IRU2 | Tpm4 | 0.64 | 3.00E-07 |
| P97429 | Anxa4 | 0.63 | 3.23E-05 |
| P57780 | Actn4 | 0.63 | 0.00E+00 |
| Q99JB2 | Stoml2 | 0.63 | 6.05E-03 |
| O88322 | Nid2 | 0.62 | 2.31E-08 |
| Q9DB73 | Cyb5r1 | 0.60 | 4.94E-03 |
| P42208 | Sept2 | 0.60 | 1.43E-05 |
| Q9CQE8 | NA | 0.60 | 5.24E-03 |
| Q8BMK4 | Ckap4 | 0.60 | 3.19E-07 |
| Q9Z1Q5 | Clic1 | 0.59 | 2.51E-04 |
| P21981 | Tgm2 | 0.59 | 0.00E+00 |
| Q91X72 | Hpx | 0.59 | 1.11E-14 |
| P01868;P01869 | Ighg1 | -0.60 | 2.53E-03 |
| Q9D1G3 | Hhatl | -0.60 | 9.45E-09 |
| Q02013 | Aqp1 | -0.67 | 3.04E-05 |
| P23242 | Gja1 | -0.71 | 6.38E-07 |
| Q8R2G4 | Art3 | -0.75 | 6.44E-07 |
| O70622 | Rtn2 | -0.90 | 3.98E-06 |
| P61014 | Pln | -0.91 | 1.56E-04 |
| Q99PR8 | Hspb2 | 0.58 | 4.18E-05 |
| P61027 | Rab10 | 0.58 | 9.37E-03 |
| P24527 | Lta4h | 0.58 | 1.94E-07 |
| Q9JMH6 | Txnrd1 | 0.58 | 6.16E-04 |
| P13020;CON__Q3SX14 | Gsn | 0.58 | 2.46E-11 |
| P48036 | Anxa5 | 0.57 | 9.30E-14 |
| P63260 | Actg1 | 0.57 | 1.63E-10 |
| Q3U0V1 | Khsrp | 0.56 | 1.04E-02 |
| Q9R0P5 | Dstn | 0.56 | 1.52E-07 |
| Q99P72 | Rtn4 | 0.56 | 5.24E-04 |
| P21619 | Lmnb2 | 0.55 | 1.62E-03 |
| P40124 | Cap1 | 0.54 | 7.53E-06 |
| P48678 | Lmna | 0.54 | 0.00E+00 |
| Q8VHX6 | Flnc | 0.53 | 0.00E+00 |
| O70209 | Pdlim3 | 0.53 | 6.77E-04 |
| P32261;CON__P41361 | Serpinc1 | 0.53 | 8.66E-05 |
| Q07076 | Anxa7 | 0.53 | 6.98E-07 |
| P14602 | Hspb1 | 0.52 | 1.41E-09 |
| Q9Z1Z0 | Uso1 | 0.52 | 2.05E-03 |
| Q9CQV8;O70456 | Ywhab | 0.52 | 1.55E-04 |
| O35206 | Col15a1 | 0.52 | 1.52E-09 |
| Q9DBJ1 | Pgam1 | 0.52 | 5.62E-05 |
| Q99KK7 | Dpp3 | 0.51 | 7.12E-03 |
| O88456;Q9D7J7 | Capns1 | 0.51 | 1.09E-03 |
| P05213 | Tuba1b | 0.51 | 0.00E+00 |
| P99024 | Tubb5 | 0.51 | 6.44E-05 |
| Q8C1B7;Q9R1T4 | Sept11 | 0.50 | 3.83E-05 |
| Q9WUA3 | Pfkp | 0.50 | 3.13E-03 |
| Q80XN0 | Bdh1 | 0.50 | 1.39E-03 |
| Q3UPL0 | Sec31a | 0.50 | 9.03E-03 |
| Q8BTM8 | Flna | 0.50 | 2.22E-16 |
| Q9CZS1;Q9JHW9 | Aldh1b1 | 0.49 | 2.90E-05 |
| P16045 | Lgals1 | 0.49 | 1.06E-05 |
| P63254 | Crip1 | 0.49 | 3.76E-03 |
| P18760 | Cfl1 | 0.49 | 3.34E-07 |
| Q99PT1 | Arhgdia | 0.49 | 2.57E-05 |
| Q61247 | Serpinf2 | 0.49 | 5.09E-03 |
| Q99KJ8 | Dctn2 | 0.48 | 1.48E-05 |
| P42669 | Pura | 0.48 | 8.60E-03 |
| Q3URD3 | Slmap | 0.48 | 8.94E-06 |
| P20918;CON__P06868 | Plg | 0.48 | 2.58E-09 |
| P08226 | Apoe | 0.48 | 1.83E-03 |
| P49312 | Hnrnpa1 | 0.48 | 1.36E-04 |
| O70492 | Snx3 | 0.47 | 3.89E-03 |
| Q9QXC1 | Fetub | 0.47 | 8.99E-03 |
| P06801 | Me1 | 0.47 | 3.12E-05 |
| P40142 | Tkt | 0.47 | 9.85E-05 |
| P14211 | Calr | 0.47 | 5.98E-08 |
| Q9D1M0 | Sec13 | 0.47 | 1.41E-03 |
| Q6P069 | Sri | 0.46 | 1.06E-03 |
| O08997 | Atox1 | 0.46 | 4.07E-03 |
| P01872 | Ighm | 0.46 | 2.14E-05 |
| Q60605;Q8CI43 | Myl6 | 0.45 | 1.20E-04 |
| Q9QUI0 | Rhoa | 0.45 | 5.18E-04 |
| P28665;P28666 | Mug1 | 0.45 | 0.00E+00 |
| Q99JY9;Q641P0 | Actr3 | 0.45 | 3.00E-04 |
| Q9D1A2 | Cndp2 | 0.45 | 4.70E-05 |
| P11276 | Fn1 | 0.45 | 5.80E-09 |
| P29699 | Ahsg | 0.45 | 4.27E-05 |
| P06728 | Apoa4 | 0.44 | 5.70E-09 |
| Q60854 | Serpinb6 | 0.44 | 1.98E-04 |
| P34022 | Ranbp1 | 0.44 | 1.01E-02 |
| Q9Z2X1 | Hnrnpf | 0.44 | 8.99E-03 |
| O08638 | Myh11 | 0.44 | 2.31E-05 |
| Q61702;CON__Q0VCM5 | Itih1 | 0.43 | 9.77E-03 |
| P63101 | Ywhaz | 0.43 | 5.94E-07 |
| P27546 | Map4 | 0.43 | 9.19E-07 |
| P26039 | Tln1 | 0.43 | 0.00E+00 |
| P37804 | Tagln | 0.43 | 5.23E-05 |
| P17742 | Ppia | 0.43 | 1.34E-06 |
| Q9JMC3 | Dnaja4 | 0.43 | 1.72E-04 |
| P24472 | Gsta4 | 0.43 | 8.98E-05 |
| Q8VCM7 | Fgg | 0.42 | 5.33E-07 |
| Q3UTJ2 | Sorbs2 | 0.42 | 2.68E-08 |
| Q64669 | Nqo1 | 0.42 | 3.42E-04 |
| P01027;CON__Q2UVX4 | C3 | 0.42 | 0.00E+00 |
| P68372;Q9D6F9;CON__ENSEMBL:ENSBTAP00000025008 | Tubb4b;Tubb4a | 0.41 | 2.91E-14 |
| Q8VDD5 | Myh9 | 0.41 | 6.32E-13 |
| O35639 | Anxa3 | 0.41 | 5.63E-05 |
| P48774 | Gstm5 | 0.41 | 6.15E-03 |
| P18242 | Ctsd | 0.41 | 2.42E-04 |
| O08677;CON__P01045-1;CON__Q2KJ62;CON__P01044-1 | Kng1 | 0.41 | 1.88E-06 |
| O08529 | Capn2 | 0.40 | 1.63E-03 |
| P27773 | Pdia3 | 0.40 | 2.64E-09 |
| P97927 | Lama4 | 0.39 | 6.25E-05 |
| P31001 | Des | 0.39 | 4.44E-16 |
| Q91ZJ5 | Ugp2 | 0.39 | 2.45E-08 |
| P35385 | Hspb7 | 0.39 | 7.14E-03 |
| Q9CWJ9 | Atic | 0.39 | 1.03E-04 |
| P84078;P61205;Q8BSL7 | Arf1;Arf3;Arf2 | 0.39 | 5.44E-05 |
| Q99L47 | St13 | 0.39 | 2.60E-04 |
| O70373 | Xirp1 | 0.39 | 3.00E-06 |
| Q9Z0N1;Q9Z0N2 | Eif2s3x;Eif2s3y | 0.39 | 7.17E-03 |
| Q99JI6 | Rap1b | 0.38 | 1.65E-03 |
| Q99PL5 | Rrbp1 | 0.38 | 9.74E-03 |
| Q5XJY5 | Arcn1 | 0.38 | 7.53E-03 |
| P19324 | Serpinh1 | 0.38 | 3.46E-04 |
| Q9DCM2 | Gstk1 | -0.39 | 4.58E-03 |
| E9Q401;E9PZQ0 | Ryr2 | -0.39 | 0.00E+00 |
| Q9ET78 | Jph2 | -0.40 | 3.22E-04 |
| Q5SW19 | Cluh | -0.40 | 9.01E-03 |
| Q8BH86 | NA | -0.40 | 1.99E-05 |
| Q9CXZ1 | Ndufs4 | -0.43 | 9.84E-06 |
| P02089 | Hbb-b2 | -0.43 | 1.11E-04 |
| P11152 | Lpl | -0.46 | 2.63E-05 |
| Q6P3A8 | Bckdhb | -0.48 | 5.74E-06 |
| Q9JKF1 | Iqgap1 | 1.01 | 1.77E-02 |
| Q9D783 | Klhl40 | 0.91 | 1.65E-02 |
| Q9EST5 | Anp32b | 0.86 | 3.11E-02 |
| O35691 | Pnn | 0.85 | 1.19E-02 |
| P01837 | NA | 0.71 | 2.67E-02 |
| Q61599 | Arhgdib | 0.68 | 2.47E-02 |
| Q9DBR7 | Ppp1r12a | 0.68 | 4.19E-02 |
| Q61879 | Myh10 | 0.60 | 1.56E-02 |
| P68373 | Tuba1c | 0.59 | 1.33E-02 |
| P61164 | Actr1a | 0.57 | 1.25E-02 |
| Q61553 | Fscn1 | 0.57 | 2.00E-02 |
| O54962 | Banf1 | 0.52 | 3.34E-02 |
| P08030 | Aprt | 0.50 | 3.61E-02 |
| P43024 | Cox6a1 | 0.49 | 4.58E-02 |
| Q60902 | Eps15l1 | 0.49 | 2.30E-02 |
| Q99LD8 | Ddah2 | 0.49 | 1.14E-02 |
| Q9WTM5 | Ruvbl2 | 0.49 | 1.63E-02 |
| P01865;P01863;P84751 | Igh-1a;Ighg | 0.49 | 1.43E-02 |
| P70362 | Ufd1l | 0.48 | 3.49E-02 |
| Q9JJU8 | Sh3bgrl | 0.48 | 1.83E-02 |
| Q9CQ65 | Mtap | 0.47 | 2.04E-02 |
| Q62048 | Pea15 | 0.47 | 1.24E-02 |
| P56376 | Acyp1 | 0.47 | 1.34E-02 |
| P23198 | Cbx3 | 0.47 | 1.39E-02 |
| P61961 | Ufm1 | 0.47 | 1.48E-02 |
| Q9WUU7 | Ctsz | 0.47 | 2.84E-02 |
| P61750 | Arf4 | 0.47 | 4.27E-02 |
| Q9EP69 | Sacm1l | 0.46 | 3.81E-02 |
| A6X935 | Itih4 | 0.46 | 1.55E-02 |
| Q9DAW9 | Cnn3 | 0.45 | 1.53E-02 |
| P50516 | Atp6v1a | 0.45 | 2.86E-02 |
| Q9JM76 | Arpc3 | 0.45 | 4.00E-02 |
| P08003 | Pdia4 | 0.44 | 4.37E-02 |
| P01887 | B2m | 0.44 | 2.21E-02 |
| Q8BH61 | F13a1 | 0.43 | 2.14E-02 |
| Q9D0M5 | Dynll2 | 0.42 | 1.26E-02 |
| Q66JS6;Q3UGC7 | Eif3j2;Eif3j1 | 0.41 | 2.21E-02 |
| P47963 | Rpl13 | 0.41 | 1.42E-02 |
| O35887 | Calu | 0.40 | 1.33E-02 |
| Q921T2 | Tor1aip1 | 0.39 | 3.89E-02 |
| P51125 | Cast | 0.39 | 1.74E-02 |
| O88746 | Tom1 | 0.39 | 1.50E-02 |
| Q9D832 | Dnajb4 | 0.38 | 1.33E-02 |
| Q8VCW8 | Acsf2 | 0.38 | 1.87E-02 |
| P31428 | Dpep1 | 0.38 | 2.22E-02 |
| Q9Z204 | Hnrnpc | 0.38 | 3.78E-02 |
| P27659 | Rpl3 | 0.38 | 4.18E-02 |
| P07724;CON__P02768-1 | Alb | 0.37 | 0.00E+00 |
| P21614 | Gc | 0.37 | 4.58E-06 |
| P23927 | Cryab | 0.37 | 6.02E-05 |
| P62880 | Gnb2 | 0.37 | 3.91E-05 |
| Q99KB8 | Hagh | 0.37 | 1.29E-02 |
| Q8K0E8;CON__P02676 | Fgb | 0.37 | 3.52E-05 |
| Q9QZ06 | Tollip | 0.37 | 3.65E-02 |
| Q80XB4 | Nrap | 0.37 | 2.03E-04 |
| Q1XH17 | Trim72 | 0.37 | 6.42E-08 |
| Q61147;CON__ENSEMBL:ENSBTAP00000031900 | Cp | 0.36 | 2.27E-06 |
| Q64010 | Crk | 0.36 | 2.66E-02 |
| Q9CR16 | Ppid | 0.36 | 2.78E-02 |
| P80318 | Cct3 | 0.36 | 3.92E-06 |
| Q9EQH3 | Vps35 | 0.36 | 1.62E-04 |
| P26041 | Msn | 0.35 | 1.03E-07 |
| Q68FD5 | Cltc | 0.35 | 3.90E-12 |
| O55222 | Ilk | 0.35 | 1.08E-03 |
| P47753 | Capza1 | 0.35 | 2.57E-02 |
| Q9R0X4;Q32MW3 | Acot9;Acot10 | 0.35 | 8.31E-04 |
| Q05816 | Fabp5 | 0.35 | 2.57E-03 |
| P15532 | Nme1 | 0.34 | 4.11E-02 |
| Q921I1;CON__Q29443;CON__Q0IIK2 | Tf | 0.34 | 7.11E-15 |
| Q6PHZ2 | Camk2d | 0.34 | 1.82E-04 |
| O88587 | Comt | 0.34 | 3.91E-03 |
| Q9Z1Q9 | Vars | 0.34 | 3.85E-02 |
| P70333 | Hnrnph2 | 0.34 | 2.89E-03 |
| Q8BG05 | Hnrnpa3 | 0.34 | 1.85E-02 |
| Q6ZQ38 | Cand1 | 0.34 | 2.77E-02 |
| O88544 | Cops4 | 0.34 | 1.68E-02 |
| Q9QYB1 | Clic4 | 0.34 | 1.46E-03 |
| Q9CVB6 | Arpc2 | 0.34 | 4.56E-02 |
| Q8VIJ6 | Sfpq | 0.34 | 6.19E-03 |
| P00493 | Hprt1 | 0.34 | 2.93E-02 |
| P63001;P60764;Q05144 | Rac1;Rac3;Rac2 | 0.33 | 3.38E-03 |
| P50580 | Pa2g4 | 0.33 | 4.13E-02 |
| Q01339 | Apoh | 0.33 | 1.85E-04 |
| P34884 | Mif | 0.33 | 1.01E-02 |
| P19221;CON__P00735 | F2 | 0.32 | 1.70E-02 |
| P08113 | Hsp90b1 | 0.32 | 1.47E-07 |
| Q60864 | Stip1 | 0.32 | 2.89E-05 |
| Q9Z2U0;Q9CWH6 | Psma7 | 0.32 | 6.11E-03 |
| Q61554 | Fbn1 | 0.31 | 3.83E-09 |
| P48722 | Hspa4l | 0.31 | 2.89E-02 |
| Q8VDJ3 | Hdlbp | 0.31 | 1.59E-02 |
| Q8CIE6 | Copa | 0.31 | 3.99E-02 |
| O35226 | Psmd4 | 0.31 | 3.46E-02 |
| Q9DBG3 | Ap2b1 | 0.31 | 3.51E-02 |
| Q8K4Z3 | Apoa1bp | 0.30 | 7.26E-03 |
| Q91VI7 | Rnh1 | 0.30 | 4.57E-05 |
| P17710 | Hk1 | 0.30 | 2.23E-09 |
| Q61838;Q6GQT1 | A2m | 0.30 | 2.61E-09 |
| Q9DCD0 | Pgd | 0.30 | 3.52E-02 |
| O88342 | Wdr1 | 0.30 | 1.40E-05 |
| Q921F2 | Tardbp | 0.30 | 4.09E-03 |
| Q04857 | Col6a1 | 0.30 | 2.61E-03 |
| Q8R001 | Mapre2 | 0.30 | 1.43E-02 |
| P10605 | Ctsb | 0.30 | 2.82E-02 |
| P60766;Q8R527 | Cdc42 | 0.29 | 2.75E-02 |
| Q02788 | Col6a2 | 0.29 | 1.46E-02 |
| P60843 | Eif4a1 | 0.29 | 1.28E-04 |
| O70435 | Psma3 | 0.29 | 4.05E-02 |
| Q99JR5 | Tinagl1 | 0.29 | 4.67E-02 |
| P29341 | Pabpc1 | 0.29 | 4.55E-02 |
| Q61768;P28738;P33175 | Kif5b | 0.29 | 2.03E-03 |
| Q8CDN6 | Txnl1 | 0.28 | 2.96E-03 |
| Q91Z53 | Grhpr | 0.28 | 7.19E-03 |
| P07309 | Ttr | 0.27 | 3.83E-02 |
| P59999 | Arpc4 | 0.27 | 4.72E-02 |
| Q9CYR0 | Ssbp1 | 0.27 | 4.26E-02 |
| Q00623;CON__P15497 | Apoa1 | 0.27 | 5.43E-05 |
| P10493 | Nid1 | 0.27 | 1.19E-06 |
| P11983 | Tcp1 | 0.27 | 1.85E-03 |
| Q64105 | Spr | 0.27 | 9.40E-04 |
| P68037 | Ube2l3 | 0.26 | 2.47E-02 |
| Q61316 | Hspa4 | 0.26 | 1.65E-05 |
| E9PV24;CON__P02672 | Fga | 0.26 | 2.60E-03 |
| P61982 | Ywhag | 0.26 | 1.43E-02 |
| P54728 | Rad23b | 0.26 | 4.68E-02 |
| Q9Z2U1 | Psma5 | 0.25 | 2.00E-02 |
| P62259 | Ywhae | 0.25 | 2.65E-04 |
| Q9CPV4 | Glod4 | 0.25 | 4.85E-03 |
| P60335;P57724 | Pcbp1 | 0.25 | 5.80E-03 |
| P61979 | Hnrnpk | 0.25 | 7.97E-04 |
| P80315 | Cct4 | 0.25 | 1.83E-02 |
| O08788 | Dctn1 | 0.25 | 2.76E-02 |
| Q60994 | Adipoq | 0.25 | 3.35E-02 |
| Q01730 | Rsu1 | 0.25 | 3.41E-02 |
| Q9D1X0 | Nol3 | 0.25 | 3.62E-02 |
| Q9D0E1 | Hnrnpm | 0.25 | 5.56E-03 |
| P50247 | Ahcy | 0.25 | 1.61E-02 |
| Q91YE8 | Synpo2 | 0.25 | 1.02E-02 |
| Q9R1P0 | Psma4 | 0.25 | 2.74E-02 |
| P63017 | Hspa8 | 0.24 | 1.26E-06 |
| Q9Z2W0 | Dnpep | 0.24 | 8.32E-03 |
| P80316 | Cct5 | 0.24 | 1.72E-02 |
| Q9QYJ0 | Dnaja2 | 0.24 | 9.10E-03 |
| Q7TQI3 | Otub1 | 0.24 | 3.24E-02 |
| Q9D819 | Ppa1 | 0.23 | 1.06E-02 |
| Q02053;P31254 | Uba1 | 0.23 | 5.37E-05 |
| Q9CPY7 | Lap3 | 0.23 | 2.20E-03 |
| Q3TXS7 | Psmd1 | 0.23 | 3.07E-02 |
| P10649;O35660 | Gstm1 | 0.23 | 5.34E-03 |
| Q5EBG6 | Hspb6 | 0.23 | 1.85E-02 |
| P28271 | Aco1 | 0.23 | 1.58E-02 |
| Q9JK92 | Hspb8 | 0.22 | 4.81E-02 |
| A2AAJ9 | Obscn | 0.22 | 1.38E-12 |
| Q61598 | Gdi2 | 0.22 | 2.13E-03 |
| P80314 | Cct2 | 0.22 | 1.65E-02 |
| P80313 | Cct7 | 0.22 | 2.37E-02 |
| Q99LX0 | Park7 | 0.22 | 6.06E-03 |
| P11499 | Hsp90ab1 | 0.21 | 2.58E-06 |
| P24270 | Cat | 0.21 | 9.16E-03 |
| Q71LX4 | Tln2 | 0.21 | 3.24E-06 |
| P14206 | Rpsa | 0.21 | 1.86E-02 |
| P35700 | Prdx1 | 0.20 | 5.66E-04 |
| Q8CIB5 | Fermt2 | 0.20 | 1.55E-02 |
| Q9WTI7 | Myo1c | 0.20 | 2.84E-03 |
| P50462 | Csrp3 | 0.20 | 3.73E-02 |
| P14824 | Anxa6 | 0.20 | 5.72E-05 |
| Q9D6Y9 | Gbe1 | 0.20 | 3.88E-02 |
| O70400 | Pdlim1 | 0.20 | 3.34E-02 |
| Q61207 | Psap | 0.20 | 3.21E-02 |
| P16858;Q64467 | Gapdh | 0.20 | 6.80E-04 |
| Q78ZA7 | Nap1l4 | 0.19 | 3.91E-02 |
| P80317;Q61390 | Cct6a | 0.19 | 3.74E-02 |
| Q3UM45 | Ppp1r7 | 0.19 | 4.80E-02 |
| Q99LF4 | Rtcb | 0.19 | 2.32E-02 |
| P45591 | Cfl2 | 0.19 | 2.15E-02 |
| O88569 | Hnrnpa2b1 | 0.19 | 7.02E-03 |
| Q8CGC7 | Eprs | 0.19 | 1.23E-02 |
| Q9CYT6 | Cap2 | 0.19 | 4.22E-02 |
| Q8VEK3 | Hnrnpu | 0.19 | 1.59E-02 |
| P14733 | Lmnb1 | 0.18 | 4.17E-02 |
| Q01853 | Vcp | 0.18 | 3.08E-04 |
| Q8BG32 | Psmd11 | 0.18 | 1.76E-02 |
| Q8K370 | Acad10 | 0.18 | 2.69E-02 |
| P29758 | Oat | 0.18 | 1.97E-02 |
| Q8VBT1 | Txlnb | 0.18 | 4.26E-02 |
| Q9EQP2 | Ehd4 | 0.18 | 4.40E-03 |
| Q05793 | Hspg2 | 0.18 | 1.29E-06 |
| P16546 | Sptan1 | 0.17 | 1.08E-08 |
| P15626 | Gstm2 | 0.17 | 3.05E-02 |
| Q9QXS1 | Plec | 0.17 | 1.26E-09 |
| Q8CGK3 | Lonp1 | 0.16 | 2.75E-02 |
| Q61292 | Lamb2 | 0.16 | 7.40E-03 |
| P70296;Q8VIN1 | Pebp1 | 0.15 | 4.19E-02 |
| Q64727 | Vcl | 0.14 | 1.88E-03 |
| Q62261 | Sptbn1 | 0.13 | 3.02E-04 |
| P62631 | Eef1a2 | 0.12 | 2.34E-02 |
| Q9JKS4 | Ldb3 | 0.11 | 8.26E-03 |
| P58252 | Eef2 | 0.10 | 4.25E-02 |
| Q62234 | Myom1 | -0.06 | 7.89E-03 |
| P08249 | Mdh2 | -0.09 | 4.84E-02 |
| Q9JI91 | Actn2 | -0.10 | 1.87E-04 |
| Q6PB66 | Lrpprc | -0.11 | 7.38E-03 |
| P63038 | Hspd1 | -0.11 | 9.95E-03 |
| P45376 | Akr1b1 | -0.11 | 1.76E-02 |
| P38647 | Hspa9 | -0.11 | 3.98E-02 |
| P11531 | Dmd | -0.12 | 1.51E-03 |
| A2ASS6;REV__Q8K1T1 | Ttn | -0.12 | 0.00E+00 |
| Q0II04 | Nebl | -0.12 | 2.37E-02 |
| P05202 | Got2 | -0.12 | 7.07E-03 |
| Q91WD5 | Ndufs2 | -0.12 | 1.42E-02 |
| P70670;Q60817 | Naca | -0.13 | 1.31E-04 |
| Q07417 | Acads | -0.13 | 1.05E-02 |
| Q91ZA3 | Pcca | -0.14 | 3.88E-03 |
| Q8JZQ2;Q920A7 | Afg3l2 | -0.14 | 4.51E-02 |
| Q9Z0X1 | Aifm1 | -0.14 | 8.11E-03 |
| Q8JZN5 | Acad9 | -0.14 | 2.36E-02 |
| Q60675;P19137 | Lama2 | -0.15 | 1.04E-05 |
| P67778 | Phb | -0.15 | 4.54E-02 |
| Q9D6J6 | Ndufv2 | -0.15 | 3.19E-02 |
| Q02566 | Myh6 | -0.15 | 2.22E-15 |
| P21550 | Eno3 | -0.15 | 4.97E-03 |
| Q9QYG0 | Ndrg2 | -0.15 | 1.28E-02 |
| Q9CQ62 | Decr1 | -0.15 | 1.22E-02 |
| Q9DB77 | Uqcrc2 | -0.15 | 5.03E-03 |
| O55126 | Gbas | -0.15 | 1.58E-02 |
| Q8CAQ8 | Immt | -0.15 | 2.02E-04 |
| Q7TQ48 | Srl | -0.15 | 4.00E-04 |
| O35855 | Bcat2 | -0.15 | 4.12E-02 |
| Q8BFR5 | Tufm | -0.16 | 1.42E-03 |
| Q99LC3 | Ndufa10 | -0.16 | 4.24E-02 |
| Q9CQA3 | Sdhb | -0.16 | 3.14E-03 |
| Q91YT0 | Ndufv1 | -0.16 | 4.99E-04 |
| Q8K2B3 | Sdha | -0.16 | 3.38E-05 |
| Q9D0K2 | Oxct1 | -0.17 | 2.39E-03 |
| Q91VD9 | Ndufs1 | -0.17 | 7.13E-07 |
| O70468 | Mybpc3 | -0.17 | 2.02E-10 |
| P99029 | Prdx5 | -0.17 | 8.68E-03 |
| P19783 | Cox4i1 | -0.17 | 9.81E-03 |
| Q99J39 | Mlycd | -0.18 | 2.47E-02 |
| P11404 | Fabp3 | -0.18 | 4.62E-03 |
| P56480 | Atp5b | -0.18 | 4.56E-06 |
| P62897;CON__P62894;P00015 | Cycs | -0.18 | 2.21E-02 |
| P07310 | Ckm | -0.18 | 9.50E-05 |
| P54071 | Idh2 | -0.18 | 3.37E-05 |
| Q9DCS9 | Ndufb10 | -0.18 | 1.22E-02 |
| Q9CQZ5 | Ndufa6 | -0.19 | 1.56E-02 |
| Q3ULD5 | Mccc2 | -0.19 | 1.34E-02 |
| Q9EQ20 | Aldh6a1 | -0.19 | 1.73E-04 |
| Q9CZU6 | Cs | -0.19 | 9.54E-05 |
| Q60936 | Adck3 | -0.19 | 2.97E-03 |
| Q9ERS2 | Ndufa13 | -0.19 | 2.89E-02 |
| Q9JJW5 | Myoz2 | -0.19 | 2.21E-04 |
| Q8R164 | Bphl | -0.20 | 4.89E-02 |
| Q9WUR2;Q78JN3 | Eci2 | -0.20 | 2.55E-02 |
| Q8C0M9 | Asrgl1 | -0.20 | 4.26E-02 |
| P00405 | Mtco2 | -0.20 | 3.59E-02 |
| Q9CR62 | Slc25a11 | -0.20 | 2.20E-03 |
| Q921G7 | Etfdh | -0.20 | 1.54E-05 |
| Q6P8J7;P30275 | Ckmt2 | -0.20 | 1.69E-04 |
| P09542 | Myl3 | -0.20 | 6.36E-04 |
| P45952 | Acadm | -0.20 | 1.31E-05 |
| Q99L13 | Hibadh | -0.20 | 1.08E-02 |
| P16332 | Mut | -0.20 | 4.38E-03 |
| Q9CQC7 | Ndufb4 | -0.20 | 2.40E-02 |
| O35459 | Ech1 | -0.21 | 1.29E-03 |
| Q9D0M3 | Cyc1 | -0.21 | 1.43E-03 |
| Q8K3J1 | Ndufs8 | -0.21 | 3.03E-03 |
| O35129 | Phb2 | -0.21 | 3.11E-03 |
| P03911 | Mtnd4 | -0.21 | 3.53E-02 |
| P40630 | Tfam | -0.22 | 3.38E-02 |
| Q9D6J5 | Ndufb8 | -0.22 | 3.18E-03 |
| P56135 | Atp5j2 | -0.22 | 4.58E-02 |
| Q8BH95 | Echs1 | -0.22 | 3.24E-04 |
| Q9CQQ7 | Atp5f1 | -0.22 | 3.19E-04 |
| Q91VM9 | Ppa2 | -0.22 | 3.23E-03 |
| P49813;Q9JKK7 | Tmod1 | -0.22 | 1.11E-03 |
| P51667;Q62082 | Myl2 | -0.22 | 2.74E-04 |
| Q9CQ75 | Ndufa2 | -0.22 | 4.82E-02 |
| Q03265 | Atp5a1 | -0.23 | 9.46E-09 |
| O09161 | Casq2 | -0.23 | 2.43E-04 |
| P05201 | Got1 | -0.23 | 1.05E-07 |
| Q9QXX4 | Slc25a13 | -0.23 | 1.93E-05 |
| Q60759 | Gcdh | -0.24 | 2.06E-02 |
| Q9CPP6 | Ndufa5 | -0.24 | 1.28E-02 |
| Q9DCT2 | Ndufs3 | -0.24 | 6.26E-04 |
| Q9CR68 | Uqcrfs1 | -0.24 | 1.51E-05 |
| P48962 | Slc25a4 | -0.24 | 1.68E-07 |
| P62082 | Rps7 | -0.24 | 4.08E-02 |
| P97807 | Fh | -0.24 | 1.28E-06 |
| P48787 | Tnni3 | -0.24 | 1.92E-04 |
| O70548 | Tcap | -0.24 | 4.66E-02 |
| Q9D023 | Mpc2 | -0.24 | 3.59E-02 |
| Q99LY9 | Ndufs5 | -0.24 | 1.18E-02 |
| Q8VEM8 | Slc25a3 | -0.24 | 2.88E-04 |
| Q9CR21 | Ndufab1 | -0.25 | 3.78E-02 |
| P14152 | Mdh1 | -0.25 | 3.58E-05 |
| Q8BH59 | Slc25a12 | -0.25 | 7.80E-09 |
| P97450 | Atp5j | -0.25 | 6.37E-04 |
| Q3TLP5 | Echdc2 | -0.25 | 2.84E-02 |
| O08532 | Cacna2d1 | -0.25 | 3.64E-02 |
| Q99JY0 | Hadhb | -0.26 | 4.19E-08 |
| Q91VR2 | Atp5c1 | -0.26 | 2.59E-04 |
| Q9D855 | Uqcrb | -0.26 | 2.20E-04 |
| P50752 | Tnnt2 | -0.26 | 8.75E-06 |
| P70414 | Slc8a1 | -0.26 | 1.30E-02 |
| Q9DCX2 | Atp5h | -0.26 | 6.56E-04 |
| O55143 | Atp2a2 | -0.26 | 6.66E-16 |
| Q8QZT1 | Acat1 | -0.26 | 5.13E-07 |
| Q9DC69 | Ndufa9 | -0.26 | 3.65E-07 |
| Q99KQ4 | Nampt | -0.26 | 2.11E-04 |
| O35857 | Timm44 | -0.27 | 5.11E-04 |
| Q9D8B4 | Ndufa11 | -0.27 | 3.39E-02 |
| Q6PIE5;Q6PIC6;Q9Z1W8 | Atp1a2;Atp1a3 | -0.27 | 8.22E-03 |
| Q9Z2Z6 | Slc25a20 | -0.28 | 9.93E-05 |
| Q9DB20 | Atp5o | -0.28 | 1.48E-03 |
| P16125 | Ldhb | -0.28 | 2.72E-06 |
| P0DN34 | NA | -0.28 | 4.15E-02 |
| Q99NB1 | Acss1 | -0.28 | 1.48E-07 |
| Q9CZ13 | Uqcrc1 | -0.28 | 3.69E-09 |
| Q9CQH3 | Ndufb5 | -0.28 | 1.00E-02 |
| Q922B1 | Macrod1 | -0.29 | 9.06E-03 |
| Q4VAE3 | Tmem65 | -0.30 | 2.13E-02 |
| Q99LC5 | Etfa | -0.30 | 7.70E-08 |
| P52825 | Cpt2 | -0.30 | 1.94E-09 |
| Q8VD26 | Tmem143 | -0.30 | 4.07E-02 |
| Q9CPU0 | Glo1 | -0.30 | 2.77E-04 |
| Q9DCW4 | Etfb | -0.30 | 6.57E-07 |
| Q9Z2I9 | Sucla2 | -0.30 | 2.06E-09 |
| Q64152 | Btf3 | -0.31 | 3.74E-02 |
| P03921 | Mtnd5 | -0.31 | 1.17E-02 |
| P41216;Q8JZR0 | Acsl1 | -0.31 | 2.00E-15 |
| Q3UIU2 | Ndufb6 | -0.31 | 2.58E-02 |
| Q9CPU4 | Mgst3 | -0.31 | 2.88E-02 |
| Q8BK30 | Ndufv3 | -0.32 | 1.17E-02 |
| P50544 | Acadvl | -0.32 | 2.22E-16 |
| Q9CRB8 | Mtfp1 | -0.33 | 6.66E-03 |
| P50136 | Bckdha | -0.33 | 2.38E-04 |
| P18572 | Bsg | -0.33 | 6.60E-05 |
| Q65CL1 | Ctnna3 | -0.33 | 5.39E-05 |
| Q61941 | Nnt | -0.33 | 0.00E+00 |
| P14094 | Atp1b1 | -0.34 | 8.16E-05 |
| Q62351 | Tfrc | -0.34 | 2.71E-02 |
| Q8R1I1 | Uqcr10 | -0.34 | 5.24E-03 |
| Q9CXJ4 | Abcb8 | -0.35 | 1.03E-04 |
| Q8VDN2;Q64436 | Atp1a1 | -0.35 | 1.33E-15 |
| P47934 | Crat | -0.36 | 3.92E-10 |
| P42125 | Eci1 | -0.36 | 4.93E-09 |
| Q9QYR9 | Acot2 | -0.36 | 1.96E-06 |
| P51637 | Cav3 | -0.37 | 1.22E-02 |
| P03888 | Mtnd1 | -0.37 | 4.14E-02 |
| Q8BMS1 | Hadha | -0.37 | 0.00E+00 |
| Q8BWT1 | Acaa2 | -0.38 | 2.44E-15 |
| P53395 | Dbt | -0.38 | 1.96E-06 |
| Q8BYM8 | Cars2 | -0.47 | 4.44E-02 |
| Q9D2N4;O70585 | Dtna | -0.57 | 4.79E-02 |
| Q8BGY7 | Fam210a | -0.65 | 1.35E-02 |
| Q9D1J3 | Sarnp | -0.72 | 2.46E-02 |
| Q9CXR1 | Dhrs7 | -0.74 | 2.19E-02 |
| Q9CQI3;Q9ERL7 | Gmfb;Gmfg | -0.87 | 1.32E-02 |

## Supplemental Table 7 – HIF1a target genes in transcriptome analysis

| Upregulated potential HIF1a target genes in the transcriptome analysis | | |
| --- | --- | --- |
| gene symbol | **log_2_fc** | **pval** |
| Ubxn10 | 1,21 | 1,52E-03 |
| Tfrc | 0,99 | 3,85E-03 |
| Cenpf | 1,02 | 7,66E-03 |
| Egln3 | 0,87 | 1,05E-02 |
| Emc2 | 0,82 | 1,52E-02 |
| Pde3a | 0,81 | 2,06E-02 |
| Rab21 | 0,75 | 2,77E-02 |
| Uba3 | 0,74 | 3,14E-02 |
| Ovol1 | 0,97 | 3,32E-02 |
| Arntl | 0,87 | 3,45E-02 |
| Prkaa2 | 0,70 | 3,81E-02 |
| Klk1b22 | 0,80 | 3,98E-02 |
| Pggt1b | 0,77 | 4,00E-02 |
| Ccl9 | 0,81 | 4,58E-02 |

## Supplemental Table 8 – HIF1a target genes in proteome analysis

| Upregulated potential HIF1a target proteins in the transcriptome analysis | | | |
| --- | --- | --- | --- |
| protein_accession | **gene symbol** | **log_2_fc** | **pval** |
|  |  |  |  |
| P20152;P03995;P46660;P08551;P08553 | Vim | 1,286558338 | 0 |
| O08553 | Dpysl2 | 0,724744536 | 0 |
| P26039 | Tln1 | 0,431862354 | 0 |
| Q9JII6 | Akr1a1 | 0,68941303 | 4,613E-10 |
| P17710 | Hk1 | 0,302140406 | 2,2317E-09 |
| Q61147 | Cp | 0,364560883 | 2,26666E-06 |
| O88342 | Wdr1 | 0,300107321 | 1,3981E-05 |
| Q60864 | Stip1 | 0,316502348 | 2,88627E-05 |
| P62880 | Gnb2 | 0,372791119 | 3,914E-05 |
| Q02053;P31254 | Uba1 | 0,234644774 | 5,36791E-05 |
| Q9CRB6 | Tppp3 | 1,148121707 | 0,000284464 |
| Q62261 | Sptbn1 | 0,128995916 | 0,000302211 |
| P19324 | Serpinh1 | 0,379038465 | 0,000346448 |
| P16858;Q64467 | Gapdh | 0,195155543 | 0,000679866 |
| P63001;P60764;Q05144 | Rac1* | 0,334695232 | 0,003376031 |
| Q8K4G1 | Ltbp4 | 0,826201934 | 0,00372026 |
| Q9Z2U0;Q9CWH6 | Psma7 | 0,315063598 | 0,006107014 |
| Q3UPL0 | Sec31a | 0,499653571 | 0,009032947 |
| P61027 | Rab10 | 0,581370251 | 0,00936544 |
| Q61702 | Itih1 | 0,43321048 | 0,009773081 |
| P34884 | Mif | 0,328639655 | 0,010113102 |
| O35887 | Calu | 0,399230141 | 0,013325538 |
| Q8R001 | Mapre2 | 0,296927628 | 0,014275056 |
| Q9WTM5 | Ruvbl2 | 0,485371021 | 0,01626198 |
| P51125 | Cast | 0,387505336 | 0,017386356 |
| Q8BG05 | Hnrnpa3 | 0,338381083 | 0,018516296 |
| Q6ZQ38 | Cand1 | 0,33650574 | 0,027705364 |
| P50462 | Csrp3 | 0,202838109 | 0,037298673 |
| Q9D6Y9 | Gbe1 | 0,200278824 | 0,038840798 |
| P29341 | Pabpc1 | 0,288355171 | 0,045492104 |
| Q9CVB6 | Arpc2 | 0,335594566 | 0,045590996 |
| * not distinguashle to Rac2 and Rac3 | | | |

## Supplemental Table 9 - Litter Distribution

| **α-MHC^719/+^/cHIF1aKO** **Mice and α-MHC^719/+^ HCM Mice litter size. male/ female ratio and genotype distribution** | | | | | | |
| --- | --- | --- | --- | --- | --- | --- |
| **Mouse Model** | | | **Distribution of Gender at Birth (%)** | | **Distribution of Genotype at Birth (%)** | |
| **α-MHC^719/+^/ cHIF1aKO Mice** | **Breeding Mother** | **Litter Size** | **% male offspring** | **% female**  **offspring** | **Genotype:**  **WT/cHIF1aKO** | **Genotype:**  **α-MHC^719/+^/cHIF1aKO** |
|  | α-MHC^719/+^/cHIF1aKO mother | 6.75±1.47 | 53.8±18.5 | 46.2±18.5 | 57.1±8.6 | 42.9±8.6 |
|  | WT/cHIF1aKO mother | 4.5±0.43 | 55±7.1 | 0.45±7.1 | 48.6±12.1 | 51.4±12.1 |
| **α-MHC^719/+^**  **HCM Mice** | **Breeding Mother** | **Litter Size** | **% male offspring** | **% female**  **offspring** | **Genotype:**  **WT** | **Genotype:**  **α-MHC^719/+^** |
|  | α-MHC^719/+^ mother | 4.82±0.52 | 58.4±6.8* | 41.6±6.8* | 64.4±33.6 | 35.6±33.6 |
|  | WT mother | 3.31±0.7 | 58.2±11.6 | 42±11.6 | 59.9±21.7 | 40.1±21.7 |
| Values ±standard deviation *: p= 0.029 in one-sample t-test compared to a 50% distribution | | | | | | |

## Supplemental Table 10 – Enriched mechanistic pathways in α-MHC^719/+^ vs WT mice

| **Enriched mechanistic pathways in α-MHC^719/+^ vs WT mice** | | | | | |
| --- | --- | --- | --- | --- | --- |
| **Hypoxia** | | | | | |
| **downregulated genes** |  |  |  |  |  |
| **Pathway ID** | **Name** | **expected Hits** | **Hits** | **enrichment** | **adj. p-value** |
| GO:0001666 | response to hypoxia | 1.00 | 9 | 9.04 | 8.27E-05 |
| GO:0070482 | response to oxygen levels | 1.43 | 9 | 6.30 | 1.02E-03 |
|  |  |  |  |  |  |
| **Oxidative stress** | | | | | |
| **upregulated genes** |  |  |  |  |  |
| GO:1903427 | negative regulation of reactive oxygen species biosynthetic process | 0.07 | 2 | 28.16 | 3.75E-02 |
| WP1496_r90720 | Oxidative Damage | 0.08 | 2 | 25.41 | 3.23E-02 |
|  |  |  |  |  |  |
| **General stress pathways** | | | | | |
| **upregulated genes** |  |  |  |  |  |
| GO:0006950 | response to stress | 5.43 | 25 | 4.60 | 3.86E-08 |
| GO:0033554 | cellular response to stress | 2.57 | 16 | 6.21 | 9.47E-07 |
| GO:0080135 | regulation of cellular response to stress | 1.29 | 7 | 5.43 | 8.79E-03 |
| **downregulated genes** |  |  |  |  |  |
| GO:0080135 | regulation of cellular response to stress | 3.67 | 14 | 3.81 | 1.40E-03 |
| GO:0033554 | cellular response to stress | 7.33 | 30 | 4.09 | 2.59E-08 |
| GO:0006950 | response to stress | 15.47 | 49 | 3.17 | 4.12E-10 |
| GO:0032872 | regulation of stress-activated MAPK cascade | 1.27 | 8 | 6.30 | 2.48E-03 |
| GO:0070302 | regulation of stress-activated protein kinase signaling cascade | 1.29 | 8 | 6.22 | 2.67E-03 |
| GO:0032874 | positive regulation of stress-activated MAPK cascade | 0.93 | 6 | 6.45 | 1.29E-02 |
| GO:0070304 | positive regulation of stress-activated protein kinase signaling cascade | 0.94 | 6 | 6.38 | 1.36E-02 |
| GO:0032873 | negative regulation of stress-activated MAPK cascade | 0.25 | 3 | 12.19 | 4.77E-02 |
|  |  |  |  |  |  |
| **Inflammatory/Immune system** | | | | | |
| **upregulated genes** |  |  |  |  |  |
| GO:0071345 | cellular response to cytokine stimulus info | 2.84 | 14 | 4.93 | 7.91E-05 |
| GO:0032691 | negative regulation of interleukin-1 beta production info | 1.96 | 11 | 5.62 | 2.92E-04 |
| GO:0032692 | negative regulation of interleukin-1 production info | 0.02 | 2 | 104.17 | 4.91E-03 |
| GO:0002699 | positive regulation of immune effector process info | 0.85 | 6 | 7.02 | 6.68E-03 |
| GO:0050778 | positive regulation of immune response info | 0.14 | 3 | 20.83 | 1.07E-02 |
| GO:0002684 | positive regulation of immune system process info | 0.03 | 2 | 65.11 | 1.08E-02 |
| GO:0002696 | positive regulation of leukocyte activation info | 0.16 | 3 | 18.60 | 1.36E-02 |
| GO:0002687 | positive regulation of leukocyte migration info | 0.04 | 2 | 54.83 | 1.41E-02 |
| GO:2000403 | positive regulation of lymphocyte migration info | 0.04 | 2 | 49.61 | 1.62E-02 |
| GO:0060907 | positive regulation of macrophage cytokine production info | 0.18 | 3 | 16.98 | 1.64E-02 |
| GO:0061081 | positive regulation of myeloid leukocyte cytokine production involved in immune response info | 0.04 | 2 | 47.35 | 1.70E-02 |
| GO:2000406 | positive regulation of T cell migration info | 0.06 | 2 | 34.72 | 2.76E-02 |
| GO:0002718 | regulation of cytokine production involved in immune response info | 1.24 | 6 | 4.85 | 2.86E-02 |
| GO:0002739 | regulation of cytokine secretion involved in immune response info | 0.50 | 4 | 8.04 | 2.88E-02 |
| GO:0002682 | regulation of immune system process info | 0.07 | 2 | 30.64 | 3.28E-02 |
| GO:0032651 | regulation of interleukin-1 beta production info | 1.30 | 6 | 4.60 | 3.43E-02 |
| GO:0050706 | regulation of interleukin-1 beta secretion info | 0.07 | 2 | 26.71 | 4.06E-02 |
| GO:0032652 | regulation of interleukin-1 production info | 0.08 | 2 | 26.04 | 4.19E-02 |
| GO:0032675 | regulation of interleukin-6 production info | 0.28 | 3 | 10.85 | 4.25E-02 |
| GO:1901623 | regulation of lymphocyte chemotaxis info | 0.59 | 4 | 6.74 | 4.60E-02 |
| GO:0050863 | regulation of T cell activation info | 0.08 | 2 | 24.23 | 4.62E-02 |
| GO:0070232 | regulation of T cell apoptotic process info | 0.30 | 3 | 10.08 | 4.96E-02 |
| **downregulated genes** |  |  |  |  |  |
| GO:0045321 | leukocyte activation | 2.64 | 9 | 3.41 | 3.68E-02 |
|  |  |  |  |  |  |
| **Cell death** | | | | | |
| **upregulated genes** |  |  |  |  |  |
| GO:0006915 | apoptotic process | 1.25 | 7 | 5.60 | 7.79E-03 |
| GO:0008219 | cell death | 1.41 | 7 | 4.97 | 1.35E-02 |
| GO:0043066 | negative regulation of apoptotic process | 1.77 | 13 | 7.36 | 3.17E-06 |
| GO:0060548 | negative regulation of cell death | 2.02 | 14 | 6.94 | 1.99E-06 |
| GO:0043069 | negative regulation of programmed cell death | 1.80 | 13 | 7.23 | 3.81E-06 |
| GO:0012501 | programmed cell death | 1.33 | 7 | 5.25 | 1.06E-02 |
| GO:0042981 | regulation of apoptotic process | 2.92 | 17 | 5.82 | 8.32E-07 |
| GO:2001233 | regulation of apoptotic signaling pathway | 0.79 | 5 | 6.35 | 2.36E-02 |
| GO:0010941 | regulation of cell death | 3.26 | 17 | 5.22 | 3.33E-06 |
| GO:0043067 | regulation of programmed cell death | 2.96 | 17 | 5.73 | 1.00E-06 |
| GO:0070232 | regulation of T cell apoptotic process | 0.08 | 2 | 24.23 | 4.62E-02 |
| **downregulated genes** |  |  |  |  |  |
| GO:0006915 | apoptotic process | 3.56 | 22 | 6.18 | 5.27E-09 |
| GO:0060561 | apoptotic signaling pathway | 1.43 | 8 | 5.60 | 4.74E-03 |
| GO:0097190 | cell death | 4.01 | 25 | 6.23 | 2.39E-10 |
| GO:0008219 | epithelial cell apoptotic process | 0.15 | 3 | 19.59 | 1.75E-02 |
| GO:1904019 | epithelial cell apoptotic process | 0.15 | 3 | 19.59 | 1.75E-02 |
| GO:0097193 | intrinsic apoptotic signaling pathway | 0.83 | 6 | 7.22 | 8.09E-03 |
| GO:0072332 | intrinsic apoptotic signaling pathway in response to DNA damage | 0.42 | 5 | 11.87 | 3.55E-03 |
| GO:0008630 | negative regulation of apoptotic process | 5.03 | 19 | 3.78 | 9.09E-05 |
| GO:0043066 | negative regulation of apoptotic signaling pathway | 1.25 | 6 | 4.81 | 4.08E-02 |
| GO:2001234 | negative regulation of cell death | 5.75 | 24 | 4.18 | 9.63E-07 |
| GO:0060548 | negative regulation of programmed cell death | 5.12 | 21 | 4.10 | 9.14E-06 |
| GO:0043069 | positive regulation of apoptotic process | 3.51 | 18 | 5.13 | 3.35E-06 |
| GO:0043065 | positive regulation of apoptotic signaling pathway | 1.00 | 9 | 9.04 | 8.27E-05 |
| GO:2001235 | positive regulation of cell death | 3.87 | 21 | 5.43 | 1.19E-07 |
| GO:0010942 | positive regulation of cysteine-type endopeptidase activity involved in apoptotic signaling pathway | 0.05 | 2 | 40.63 | 3.55E-02 |
| GO:2001269 | positive regulation of mitochondrial outer membrane permeabilization involved in apoptotic signaling pathway | 0.05 | 2 | 36.57 | 4.07E-02 |
| GO:1901030 | positive regulation of programmed cell death | 3.53 | 18 | 5.09 | 3.72E-06 |
| GO:0043068 | programmed cell death | 3.80 | 23 | 6.05 | 2.84E-09 |
| GO:0012501 | regulation of apoptotic process | 8.32 | 35 | 4.21 | 4.22E-10 |
| GO:0042981 | regulation of apoptotic signaling pathway | 2.24 | 14 | 6.24 | 1.03E-05 |
| GO:2001233 | regulation of cell death | 9.28 | 41 | 4.42 | 1.45E-12 |
| GO:0010941 | regulation of cysteine-type endopeptidase activity involved in apoptotic process | 1.12 | 6 | 5.35 | 2.73E-02 |
| GO:0043281 | regulation of neuron apoptotic process | 1.45 | 7 | 4.81 | 2.09E-02 |
| GO:0043523 | regulation of neuron apoptotic process | 1.45 | 7 | 4.81 | 2.09E-02 |
| GO:0043067 | regulation of programmed cell death | 8.44 | 37 | 4.38 | 3.54E-11 |
|  |  |  |  |  |  |
| **Cardiac/Heart/Circulation** | | | | | |
| **upregulated genes** |  |  |  |  |  |
| GO:0048514 | blood vessel morphogenesis | 0.21 | 3 | 14.21 | 2.44E-02 |
| GO:0061337 | cardiac conduction | 0.08 | 2 | 24.80 | 4.50E-02 |
| GO:0086042 | cardiac muscle cell-cardiac muscle cell adhesion | 0.01 | 2 | 148.82 | 2.75E-03 |
| GO:0060047 | heart contraction | 0.03 | 2 | 65.11 | 1.08E-02 |
| GO:0003015 | heart process | 0.04 | 2 | 47.35 | 1.70E-02 |
| GO:1903523 | negative regulation of blood circulation | 0.08 | 2 | 24.80 | 4.50E-02 |
| GO:0060452 | positive regulation of cardiac muscle contraction | 0.02 | 2 | 80.13 | 7.79E-03 |
| GO:0010613 | positive regulation of cardiac muscle hypertrophy | 0.07 | 2 | 26.71 | 4.06E-02 |
| GO:0045823 | positive regulation of heart contraction | 0.08 | 2 | 24.23 | 4.62E-02 |
| GO:0060421 | positive regulation of heart growth | 0.09 | 2 | 23.15 | 4.97E-02 |
| GO:0045765 | regulation of angiogenesis | 0.59 | 4 | 6.76 | 4.56E-02 |
| GO:0008217 | regulation of blood pressure | 0.34 | 5 | 14.55 | 1.20E-03 |
| GO:0086004 | regulation of cardiac muscle cell contraction | 0.07 | 3 | 44.65 | 1.78E-03 |
| GO:0055117 | regulation of cardiac muscle contraction | 0.13 | 5 | 38.87 | 1.83E-05 |
| GO:0010611 | regulation of cardiac muscle hypertrophy | 0.14 | 3 | 22.01 | 9.27E-03 |
| GO:0061050 | regulation of cell growth involved in cardiac muscle cell development | 0.05 | 2 | 37.21 | 2.52E-02 |
| GO:0008016 | regulation of heart contraction | 0.33 | 5 | 15.14 | 1.02E-03 |
| GO:0060420 | regulation of heart growth | 0.16 | 3 | 18.60 | 1.36E-02 |
| GO:0034103 | regulation of tissue remodeling | 0.16 | 3 | 18.38 | 1.40E-02 |
| GO:0098911 | regulation of ventricular cardiac muscle cell action potential | 0.03 | 2 | 74.41 | 8.62E-03 |
| WP1560_r106855 | MicroRNAs in Cardiomyocyte Hypertrophy | 0.16 | 3 | 19.06 | 1.97E-02 |
| **downregulated genes** |  |  |  |  |  |
| GO:0003205 | cardiac chamber development | 0.05 | 2 | 36.57 | 4.07E-02 |
| GO:0061337 | cardiac conduction | 0.23 | 3 | 13.06 | 4.11E-02 |
| GO:0086001 | cardiac muscle cell action potential | 0.18 | 3 | 17.14 | 2.33E-02 |
| GO:0060411 | cardiac septum morphogenesis | 0.45 | 4 | 8.81 | 3.18E-02 |
| GO:0086065 | cell communication involved in cardiac conduction | 0.06 | 2 | 33.24 | 4.59E-02 |
| GO:0061371 | determination of heart left/right asymmetry | 0.06 | 2 | 33.24 | 4.59E-02 |
| GO:0060971 | embryonic heart tube left/right pattern formation | 0.03 | 2 | 73.13 | 1.68E-02 |
| GO:0003143 | embryonic heart tube morphogenesis | 0.38 | 4 | 10.45 | 1.93E-02 |
| GO:0007507 | heart development | 1.42 | 8 | 5.63 | 4.70E-03 |
| GO:1903522 | regulation of blood circulation | 1.29 | 12 | 9.34 | 1.52E-06 |
| GO:0008016 | regulation of heart contraction | 0.94 | 10 | 10.63 | 6.88E-06 |
| GO:2000826 | regulation of heart morphogenesis | 0.22 | 3 | 13.71 | 3.75E-02 |
| GO:0002027 | regulation of heart rate | 0.50 | 5 | 9.94 | 6.72E-03 |
| GO:0086091 | regulation of heart rate by cardiac conduction | 0.18 | 3 | 16.62 | 2.52E-02 |
|  |  |  |  |  |  |
|  |  |  |  |  |  |
| **mTOR pathways** | | | | | |
| **upregulated genes** |  |  |  |  |  |
| GO:0032006 | regulation of TOR signaling | 0.19 | 4 | 21.26 | 1.70E-03 |
| GO:0032007 | negative regulation of TOR signaling | 0.08 | 3 | 35.51 | 3.07E-03 |
| **downregulated genes** |  |  |  |  |  |
| WP2841_r94308 | Focal Adhesion-PI3K-Akt-mTOR-signaling pathway | 1.76 | 9 | 5.11 | 5.42E-03 |
|  |  |  |  |  |  |
| **Wnt pathway** | | | | | |
| **upregulated genes** |  |  |  |  |  |
| GO:0060828 | regulation of canonical Wnt signaling pathway | 0.45 | 5 | 11.23 | 3.19E-03 |
| GO:0030177 | positive regulation of Wnt signaling pathway | 0.26 | 4 | 15.55 | 4.32E-03 |
| GO:0030111 | regulation of Wnt signaling pathway | 0.58 | 5 | 8.65 | 8.34E-03 |
| GO:0090263 | positive regulation of canonical Wnt signaling pathway | 0.20 | 3 | 15.32 | 2.06E-02 |
| **downregulated genes** |  |  |  |  |  |
| GO:0060070 | canonical Wnt signaling pathway | 0.43 | 6 | 13.89 | 4.38E-04 |
| GO:0090090 | negative regulation of canonical Wnt signaling pathway | 0.69 | 9 | 13.06 | 6.03E-06 |
| GO:2000051 | negative regulation of non-canonical Wnt signaling pathway | 0.05 | 2 | 40.63 | 3.55E-02 |
| GO:0030178 | negative regulation of Wnt signaling pathway | 0.86 | 12 | 13.89 | 2.93E-08 |
| GO:0035567 | non-canonical Wnt signaling pathway | 0.19 | 5 | 26.12 | 1.69E-04 |
| GO:0060828 | regulation of canonical Wnt signaling pathway | 1.27 | 12 | 9.46 | 1.35E-06 |
| GO:0030111 | regulation of Wnt signaling pathway | 1.65 | 15 | 9.11 | 4.29E-08 |
| GO:0016055 | Wnt signaling pathway | 1.36 | 13 | 9.55 | 3.50E-07 |
| GO:0060071 | Wnt signaling pathway. planar cell polarity pathway | 0.14 | 4 | 29.25 | 9.45E-04 |
| GO:0017147 | Wnt-protein binding | 0.17 | 4 | 23.59 | 9.22E-04 |
| KEGG:4310 | Wnt signaling pathway | 0.86 | 7 | 8.15 | 4.02E-03 |
| WP539_r71716 | Wnt Signaling Pathway NetPath | 0.59 | 5 | 8.46 | 9.85E-03 |

# References:

1 Liberzon, A. *et al.* Molecular signatures database (MSigDB) 3.0. *Bioinformatics* **27**, 1739-1740, doi:10.1093/bioinformatics/btr260 (2011).

2 Matys, V. *et al.* TRANSFAC and its module TRANSCompel: transcriptional gene regulation in eukaryotes. *Nucleic Acids Res* **34**, D108-110, doi:10.1093/nar/gkj143 (2006).

3 Matys, V. *et al.* TRANSFAC: transcriptional regulation, from patterns to profiles. *Nucleic Acids Res* **31**, 374-378, doi:10.1093/nar/gkg108 (2003).

4 Han, H. *et al.* TRRUST v2: an expanded reference database of human and mouse transcriptional regulatory interactions. *Nucleic Acids Res* **46**, D380-D386, doi:10.1093/nar/gkx1013 (2018).

5 Semenza, G. L. Hypoxia-inducible factor 1: oxygen homeostasis and disease pathophysiology. *Trends Mol Med* **7**, 345-350, doi:10.1016/s1471-4914(01)02090-1 (2001).

6 Pujato, M., Kieken, F., Skiles, A. A., Tapinos, N. & Fiser, A. Prediction of DNA binding motifs from 3D models of transcription factors; identifying TLX3 regulated genes. *Nucleic Acids Res* **42**, 13500-13512, doi:10.1093/nar/gku1228 (2014).
